# Author Correction: Multiple hominin dispersals into Southwest Asia over the past 400,000 years

**DOI:** 10.1038/s41586-021-04289-2

**Published:** 2022-01-10

**Authors:** Huw S. Groucutt, Tom S. White, Eleanor M. L. Scerri, Eric Andrieux, Richard Clark-Wilson, Paul S. Breeze, Simon J. Armitage, Mathew Stewart, Nick Drake, Julien Louys, Gilbert J. Price, Mathieu Duval, Ash Parton, Ian Candy, W. Christopher Carleton, Ceri Shipton, Richard P. Jennings, Muhammad Zahir, James Blinkhorn, Simon Blockley, Abdulaziz Al-Omari, Abdullah M. Alsharekh, Michael D. Petraglia

**Affiliations:** 1 Extreme Events Research Group, Max Planck Institutes for Chemical Ecology, the Science of Human History, and Biogeochemistry, Jena, Germany; 2 Department of Archaeology, Max Planck Institute for the Science of Human History, Jena, Germany; 3 Institute of Prehistoric Archaeology, University of Cologne, Cologne, Germany; 4 Department of Life Sciences, Natural History Museum, London, UK; 5 Pan-African Evolution Research Group, Max Planck Institute for the Science of Human History, Jena, Germany; 6 Department of Classics and Archaeology, University of Malta, Msida, Malta; 7 Department of Archaeology, Durham University, Durham, UK; 8 Centre for Quaternary Research, Department of Geography, Royal Holloway University of London, Egham, UK; 9 Department of Geography and Environmental Science, University of Reading, Reading, UK; 10 Department of Geography, King’s College London, London, UK; 11 SFF Centre for Early Sapiens Behaviour (SapienCE), University of Bergen, Bergen, Norway; 12 Australian Research Centre for Human Evolution, Griffith University, Brisbane, Queensland Australia; 13 College of Asia and the Pacific, The Australian National University, Canberra, Australia Capital Territory Australia; 14 School of Earth and Environmental Sciences, University of Queensland, Brisbane, Australia Capital Territory Australia; 15 Geochronology and Geology, Centro Nacional de Investigación sobre le Evolución Humana, Paseo de Atapuerca, Burgos, Spain; 16 Human Origins and Palaeoenvironments Research Group, School of Social Sciences, Oxford Brookes University, Oxford, UK; 17 Mansfield College, University of Oxford, Oxford, UK; 18 Institute of Archaeology, University College London, London, UK; 19 Centre of Excellence for Australian Biodiversity and Heritage, Australian National University, Canberra, Australia Capital Territory Australia; 20 School of Biological and Environmental Sciences, Liverpool John Moores University, Liverpool, UK; 21 Department of Archaeology, Hazara University, Mansehra, Pakistan; 22 Heritage Commission, Ministry of Culture, Riyadh, Saudi Arabia; 23 Department of Archaeology, College of Tourism and Archaeology, King Saud University, Riyadh, Saudi Arabia; 24 Human Origins Program, National Museum of Natural History, Smithsonian Institution, Washington, USA; 25 School of Social Science, University of Queensland, St Lucia, Queensland, Australia

**Keywords:** Archaeology, Archaeology

Correction to: *Nature* 10.1038/s41586-021-03863-y Published online 1 September 2021

In the version of this Article initially published, the department in affiliation 15 appeared incorrectly as “Geochronology and Geology, Centro Nacional de Investigación sobre le Evolución Humana, Paseo de Atapuerca, Burgos, Spain.” This has now been corrected to read “Geochronology and Geology, Centro Nacional de Investigación sobre la Evolución Humana (CENIEH), Paseo de Atapuerca, Burgos, Spain.”

Further, there was a duplication error in the Late Pleistocene lithic data and principal components analysis reported in the Supplementary Information initially published online. The error had no significant effect on the original results. A revised analysis with detailed description of the error and links to corrected and uncorrected data, scripts and replication instructions are available in the Supplementary Information accompanying this amendment, while the revised Supplementary Information appears alongside the main article. The original Article has been corrected online.

**Supplementary Information** is available in the online version of this Amendment.

## Supplementary information

Supplementary Information This file contains a revised analysis with detailed description of the error and links to corrected and uncorrected data, scripts and replication instructions.

