# Supplementary material for: Multiple hominin dispersals into Southwest Asia over the past 400,000 years
Source: Nature. 2021 Sep 1;597(7876):376–80. doi: 10.1038/s41586-021-03863-y (PMC8443443; doi:10.1038/s41586-021-03863-y)
Supplement: Supplementary file 1 — This file contains Supplementary Methods, Discussion, Tables 1–26, Figs. 1–39 and references [file 41586_2021_3863_MOESM1_ESM.pdf]

nature portfolio

https://doi.org/10.1038/s41586-021-03863-y

Supplementary information

Multiple hominin dispersals into Southwest Asia over the past 400,000 years

In the format provided by the authors and unedited

SUPPLEMENTARY INFORMATION

1. Background and context

Southwest Asia straddles the only terrestrial connection between Africa and Eurasia. Along with its history of profound climatic and environmental variation, the region is of great importance in human evolutionary studies. The palaeoanthropological record of the area, until recently only known in detail from the Levantine woodland zone, has been interpreted in distinct ways, from regional continuity[e.g. 39], through to repeated local extinctions and repopulations[e.g. 40]. Southwest Asia is currently divided between the Palearctic and Saharo-Arabian biomes (see e.g.[4,16]). The region is therefore important for both broad debates, such as the expansion of our species beyond Africa to populate most of the world in the Late Pleistocene[3], but also specific aspects of this such as the ecological context of human evolutionary processes[e.g 41]. A long running debate between northern (via Sinai) and southern (across the Red Sea) routes suggests a different character to the hominin evolutionary process. The northern route emphasises the role of environmental change in leading to the amelioration of the Saharo-Arabian desert belt which allowed human dispersals across this otherwise arid zone[3,7,9,10]. The southern route, in contrast, hypothesises a radical change in human behaviour, such as a maritime adaptation which allowed the arid zone to effectively be bypassed[42]. Southwest Asia contributes to discussions on the character and meaning of material culture variation, such as whether particular innovations were made independently in different areas, or were spread by dispersal/diffusion. Did Levallois technology, for instance, develop in one place and then spread[e.g. 43] or was it the result of repeated convergent evolution[11,44]. Genomic studies have suggested that the main admixture between *Homo sapiens* and Neanderthals, which gives non-African humans today about 2% Neanderthal DNA[45], occurred around 60-50 thousand years ago, and infer that Southwest Asia was the likely region where this occurred[6]. Subsequently, Southwest Asia has been seen as a key origin point for the transition to the Upper Palaeolithic[46,47].

In many fundamental topics of human evolutionary studies, then, Southwest Asia has a key role. A rounded understanding of Southwest Asia requires spatially and temporally representative samples. The current record is, however, biased towards the small woodland zone of the East Mediterranean Levant. The deeply stratified caves and rockshelters of the central Levant have produced significant findings (supplementary table 1), such as the possible very early *Homo sapiens* fossil from Misliya[48 but see 49], the well-known MIS 5 *Homo sapiens* fossils from Shkhul and Qafzeh, and the southernmost known Neanderthal fossils. As well as hominin fossils, there is a rich and abundant archaeological record for the Middle and Late Pleistocene[1,2,12,50]. Recent research in the area has done much to elucidate certain time periods such as the MIS 4/3 late Middle Palaeolithic[15,51]. However, it should be noted that for earlier periods, many uncertainties remain. For example, few Late Acheulean and Acheulo-Yabrudian sites have been excavated according to modern standards. And some key sites, such as Holon and Revadim, have had their chronology questioned ([e.g.1,52]). Likewise, scholars have debated the characteristics of the Levantine archaeological record. For example, some have argued that Levallois technology is common in the Late Acheulean of the area[53,54]. When it comes to the Acheulo-Yabrudian, some have argued that Levallois technology is rare, if not absent ([e.g. 11]), while others have argued that "elements of Levallois technology, if not true Levallois production, are well represented" [54, p.32]. In terms of debates such as the extent to which Levallois technology was invented once and subsequently spread, these are clearly important issues.

Figure 1 in the main text and supplementary table 1 summarise dated archaeological and hominin palaeontological material in Southwest Asia, between 50 and 500 thousand years ago. Figure 1B plots this against modern rainfall and vegetation to show the distribution of palaeoanthropological sites relative to the modern landscape. This clearly illustrates that the majority of knowledge on the area – from sites such as Tabun, Skhul, Qafzeh, Amud, Kebara, Hayonim, etc. – comes from a small area (<2,000 km^2, west of the Sea of Galilee). These sites are, and have been for hundreds of thousands of years, located in the Mediterranean woodland zone fed by winter rainfall tracking across the Atlantic, and featuring primarily Palearctic fauna[4]. Occasional sites from further inland – such as the Syrian oasis sites including Umm el Tlel, and Shanidar Cave in Iraqi Kurdistan – are likewise very much ecologically connected to the north. As Figure 1C shows, winter-rainfall dominated areas like the Levant did not change dramatically in terms of precipitation during past interglacials.

However, most of Southwest Asia, particularly the vast area south of the Levant, Turkey, and the Caspian Sea, belongs to the Saharo-Arabian biome[4]. While currently arid, much of this region was periodically transformed by the incursion of summer, tropical/monsoonal, rainfall during various periods of the Pleistocene (Fig. 1C). Building a rounded understanding of Southwest Asian prehistory requires the elucidation of the archaeological, environmental, and biogeographical records for this vast region. The Arabian Peninsula, for instance, covers 3.2 million km^2, yet until 2011 not a single dated Pleistocene archaeological site had been published[22]. To date, the Pleistocene hominin fossil record of Arabia consists of a single *Homo sapiens* fingerbone[20] and ichnofossil footprints[56], both dating to MIS 5. In recent years important archaeological advances have been made in Arabia, particularly in the far south and east[e.g. 22,23]. These primarily indicated autochthonous, localized characteristics to the record of this region. It was therefore not immediately clear how these sites related to wider regional themes such as population dispersal. To the discoverers they primarily reflected the existence of long-isolated populations living in refugial areas[22,23]. These findings suggested that Arabia could not simply be seen as a dispersal corridor, but that it had its own evolutionary dynamics, and understanding the character of these required sites of multiple time periods from the different parts of Arabia, particularly the interior zone.

While researchers often talk about the 'Levantine corridor', it is also important to recognize that the Levant also saw the repeated emergence of localized material-culture phases. In part this may reflect the relatively muted environmental fluctuations in the woodland zone – compared to regions to the south – meaning that long phases of hominin occupation were seemingly possible. For example, the Acheulo-Yabrudian occurs from around 400 ka to 250 ka[57], and is only known from the woodland zone of the Levant, north of the Dead Sea. Likewise, the early Middle Palaeolithic with its distinctive points (both elongated Levallois points and heavily retouched points) is seemingly a specifically Levantine phenomenon [e.g.11,31]. From this, it is clear that sub-regions will not tell the whole story of Southwest Asian prehistory. In particular, northern Arabia offers the possibility of connecting the records from the Levant and southern Arabia, and allowing broader scale insights into patterns of hominin demographic and behavioural change. Likewise, while hominin fossils are often iconic, their rarity means over-reliance on them should be avoided. For example, the late Middle Pleistocene hominin fossil record of the Levant is sparse and its meaning highly ambiguous (for summaries see[1,2]).

Our aim in this paper is therefore to elucidate processes of hominin behavioural and demographic change by presenting a robust composite archaeological and hydroclimate record for northern Arabia for the first time. This area is between southern Arabia and the Levant, and will therefore allow patterns of late Middle Pleistocene and Late Pleistocene change to be understood at a regional scale.

Before describing our new findings, we outline previous knowledge on Arabian prehistory and its context (see supplementary tables 1 and 2). Traditionally, Arabia has been rather downplayed in accounts of human prehistory. However, in recent years there has been a significant increase in research. It is, though, important to note that most sites are raw material procurement sources and most sites represent single phase occupations rather than sequences through time. As mentioned above, southern Arabian sites from the Late Pleistocene and Holocene have often been emphasised as having distinctive local characteristics. At Jebel Faya, for instance, at least two (A and B) of the three main published assemblages are unlike other known assemblages from elsewhere, in Arabia and beyond[22]. In Saudi Arabia, the largest country in Southwest Asia, a handful of dated Pleistocene archaeological sites are known. These have produced both MIS 5[20,25,28,58,59] and MIS 3[27] assemblages (and in one case, MIS 5 human footprints[56]). On the whole, this record has indicated more similarities with neighbouring areas of Africa and the Levant than the far south and east of Arabia has done. For instance, MIS 5 assemblages in Saudi Arabia are characterised by the kind of *Homo sapiens*-related assemblages dominated by centripetal Levallois technology that are found in neighbouring areas of Africa and the Levant[20].

Earlier time periods are currently poorly understood in Arabia. The oldest claim for human occupation currently comes from the site of Ti's al Ghadah, where bones showing human-modification and a few simple lithics were recovered in late Middle Pleistocene deposits dating to around 300-500 ka[60-62]. The only currently published chronological data for Acheulean sites are the surprisingly young, given its simple technology of large handaxes and cleavers, assemblage from the 206-76 site at Saffaqah in central Arabia[24] and the assemblage associated with sediments probably dating to MIS 9 at An Nasim in the Nefud Desert[29]. An early, MIS 7, Middle Palaeolithic occupation was indicated by a small collection of flakes from ca. 210 ka sediments at Jebel Qattar-1 at Jubbah[28], but the small sample size and rather non-diagnostic character of most pieces limited insights from this material. Hints of MIS 7 occupation have been suggested for the central-Arabian site of Umm al Sha'al, but as discussed further below this is problematic.

This relatively scant record limits the integration of these sites into inter-regional debates. Likewise, whereas chert dominates stone tool assemblages in the Levant, the disparate single-stage archaeological sites previously known from Arabia are made on a variety of lithologies, further hindering comparisons. The available information on dated Pleistocene archaeological sites for northern Arabia is summarised in Supplementary Table 2. In many cases assemblages have very small sample sizes, and chronometric age estimates are often problematic. For instance, at Ti's al Ghadah, U-series/ESR and OSL dates are somewhat incongruous with each other[18,60]. The site of Al Wusta is a rare example of a site where multiple dating methods produced consistent results[20]. The site of Umm al Sha'al demonstrates the complications of dating old and complex sediments and sparse human occupations. At this site, several hundred lithics were found in sediments dated to late MIS 5[59]. Beneath them, five lithics were found in sediments into which deep cracks extended from

the overlying deposits. OSL age estimates associated with these scattered deeper lithics gave estimates of 194 ± 21 ka and 253 ± 27 ka. The authors describe these ~MIS 7 age results – which are inverted and do not overlap at one sigma – as being “problematic with regard to saturation”[59, p.3110]. In this situation it seems problematic to accept that the site indicates an MIS 7 hominin presence. While much can be said from surface sites lacking good chronological control[e.g. 26], good dating, both absolute and relative, is essential to factor Arabian sites into inter-regional debates.

Palaeoenvironmental studies provide context to the emerging archaeological record of Arabia. Detailed studies of the stratigraphic sequences discussed in this paper – which focusses on the archaeological record – are ongoing. Our focus here is on demonstrating the stratigraphic context of the lithic assemblages recovered, in order to date them, and to offer broad characterisations of the landscape and environment during these hominin occupations. It has long been known that Pleistocene lakes had formed in presently hyper-arid northern Arabia[63], although then available radiocarbon techniques significantly underestimated the ages of these deposits. More recent work on palaeolakes[18-20,28,64] has demonstrated repeated humid periods in the Pleistocene, particularly during interglacials. While the palaeolakes of interior Arabia are currently relatively coarsely dated, their chronology is consistent with the high-resolution speleothem records from the far south and east of Arabia[e.g. 21].

Finally, there are few fossil faunal assemblages from Arabia. This is discussed more subsequently in SI 10, but we will, for now, emphasise the salient points. Firstly, the presence of hippos at multiple times in the Nefud Desert indicates the extent of increased environmental humidity. Secondly, the species composition of Arabian faunal assemblages indicates a mix of external influences and local developments (such as the evolution of the local form of oryx). One of the key poles of influence has been Africa, and taxa such as *Syncerus* and *Hippotragus* hint at close connections between Africa and Arabia – as does the current presence of baboons in southwest Arabia. In contrast, the sites of the Levantine woodland zone are consistently dominated by Palearctic fauna such as *Dama mesopotamica*[4]. In Arabia, the presence of *Equus* sp. cf. *E. hemionus* from MIS 3 site of SD-1 in Yemen may hint at Palearctic connections in the last glacial cycle[23]. Palearctic fauna such as *Equus hemionus* and *Panthera gombaszoegensis* are also among the Middle Pleistocene fauna from Ti’s al Ghadah[60], showing that Arabian fauna included Palearctic elements but that these were less prominent than African inputs. From these data the composite nature of the Arabian fossil fauna is clear. Limiting current interpretations are gaps in the fossil record for key marine isotope stages such as MIS 7 and 9.

2. Kam-4 site setting

The site of Khall Amayshan (KAM) 4 was discovered in 2013 as part of the Palaeodeserts Project. It is located at 28.028 N, 39.352 E in the western Nefud Desert, northern Saudi Arabia. The site comprises a series of relict lakebeds situated within an inter-dunal depression, ~5 kilometres into the dune field. While we have previously reported somewhat similar inter-dunal palaeolakes in the area[26] (supplementary figure 1), which seem to preserve more than one phase of lake formation, KAM-4 is unique among known sites in that it contains multiple phases of lake formation that have associated lithic assemblages and several faunal assemblages (see [33] for information on the fossils). The relict lakebeds are situated within the lee of the basin of a large barchan dune and are elevated up to 10 m above the basin floor. Smaller branching dunes extend eastwards at the northern and southern end of the basin, while palaeolake deposits interdigitate with aeolian sands at the eastern periphery of the interdune depression. Our interpretation of the site is that while major dunes either side of the site have remained relatively stable, smaller dunes have moved across the site in a conveyor belt-like fashion, changing the accommodation space for lake formation in each successive wet phase (c.f. Edgell[65] for analogous suggestions on dune movement elsewhere, although of course different process may have occurred in different areas). The subsequent removal of covering sediment has left a remarkable site where rich lithic and fossil assemblages are associated with distinct sedimentary deposits with very little evidence for mixture (discussed in following sections of the SI). Photographs of the site are shown in extended figure 1 and supplementary figures 2-4, and the characteristics of the different lake deposits summarized in supplementary table 3.

The chronometric dating of the KAM-4 deposits using luminescence dating, supplemented by U-series dating for the Northwest Lake, is discussed below in SI 5 and 6. As well as this, the relative chronological sequence based on the superposition of several of the lake beds should be emphasised, and was studied in the field (Supplementary figures 2-5). Central Lake is stratigraphically the oldest deposit. It is overlain by Northeast and Southeast Lake deposits and extends beneath the large barchan dune that borders the west of the basin. In the northern part of the site, the Northwest Lake deposits overlie those of the Northeast Lake. In the south of the site, the Southwest, Southeast, and South Lakes are not delimited by overlying sediments, and while an OSL sample was taken from within the lacustrine sequence of Southwest Lake, the Southeast and South Lake deposits are only chronometrically dated by underlying (hence maximum) ages. Understanding this site requires the integration of OSL age estimates, the relative chronology from sedimentary superposition, and supporting information from the archaeological assemblages associated with the different deposits. We systematically surveyed the entire site walking transects with a team of researchers spaced ca. 1 meter apart, and used a DGPS and total station to record all lithics as well as the topography and geomorphology of the site (Extended Figure 2, Supplementary figures 5, 9-14). All lithics and fossils were collected for subsequent analysis. Sections were opened in each deposit to study the sedimentary sequence and acquire samples for luminescence dating (Supplementary Figure 5). In the Northwest Lake, four 2 x 2 metre trenches were excavated (Supplementary Figures 6-8). These recovered both buried fossils and lithics.

The subsequent parts of this SI give information on the various studies conducted on the KAM-4 deposits and associated archaeological and fossil material from the site. It is worth emphasising at this point the unique kind of site that KAM-4 represents. As we will explore over the coming pages and in the main paper, the site preserves multiple phases of hominin

and animal occupation, over the last 400,000 years. Aside from a few examples such as JQ-1 where a lower level produced 28 lithics[28] and the multi-level site of Jebel Faya over 1,500 km southeast[22], KAM-4 is one of the only sites to preserve multiple phases of occupation in a single locality in Arabia (not including complex surface palimpsests which sometimes exist). This is important as palaeoenvironmental analysis shows the environmental setting of the site during humid phases was broadly similar through time (i.e. a relatively small lake, within an inter-dunal depression, a few kilometres from the edge of the Nefud Desert sand sea). This emphasises changing human technology and behaviour through time, rather than differences between assemblages reflecting pragmatic factors such as different kinds of raw materials. At KAM-4, technological changes have taken place in a setting, which in terms of abiotic/geomorphological processes, has remained largely consistent during phases of lake formation. Many Arabian sites represent raw material procurement localities[24,59,66]. This means that lithics are minimally shaped, and chosen pieces were removed from sites. In the case of KAM-4, by contrast, the lithics represent material that was selected and transported to the lakeshore environments by early humans. It therefore gives a very detailed insight into the kind of technology used by these groups over time. The distribution of key artefact types is shown in Supplementary Figures 9-13, while the technology of these assemblages is discussed below in SI 7.

A key factor is the large size of the KAM-4 site – it extends around 700 metres north to south and 200 metres east to west, covering a total area of around 117,000 m^2. Given the generally low density of the Arabian archaeological record this is a crucial parameter that produces large enough sample sizes for comparative analyses. If lithics had been spread across this area and deeply buried in sediments, the monumental effort to carefully excavate millions of kilograms of sediments would have been prohibitive. Similarly, without extensive geophysical survey, the parameters of different deposits would not have been visible, and even then, excavation would have been difficult as lithics are not evenly distributed in different areas. It is well known from large scale excavations of Middle Palaeolithic sites that there can be significant horizontal variation across a deposit[e.g. 32]
. KAM-4 therefore offers a remarkable opportunity to study different phases of human occupation. As we will explore below, there is little evidence for post-depositional movement of lithics.

To supplement the record of lithics and fossils associated with the different deposits, we carried out excavations in the Northwest Lake (MIS 7) to explore the hypothesised relationship between surface material and buried contexts. This confirmed the association, as we recovered both buried lithics and fossils in the lake deposit that corresponds to their discrete surface distribution. Furthermore, we conducted excavations of MIS 7 and MIS 5 archaeological sites at Jubbah to reinforce the record for these time periods with stratified material. These are discussed below, after KAM-4. Together these deposits and associated assemblages give a composite record of multiple phases of human occupation in the late Middle and Late Pleistocene. In contrast to the fragmentary sequences previously available, they allow the story of the archaeological record of northern Arabia to be told in detail for the first time.

3. KAM-4 Palaeolake Phases

3.1 Central Lake

The earliest phase of lake formation recorded at KAM-4 is a small circular basinal feature, partly buried by the lee face of the large barchan dune on the western side of the basin (Extended figures 1-3; supplementary figures 2,3,5). The relict lakebed surface is capped by highly weathered and indurated iron-stained marls, which erode out at the surface and downslope of the feature. The uppermost lip of the lake surface is elevated ~4 m above the basin floor and interdigitates with the talus slope of the adjacent and overlying Northeast Lake. Preserved lakebeds pinch-out nearer the dune face, while the centre of the basin falls to a depth of ~2 m from the relict lake edge, likely reflecting its morphology prior to desiccation.

A sequence was excavated to a depth of 70 cm to establish the character and succession of lacustrine sedimentation. Central Lake deposits are underlain by pale cream-grey, fine to medium, moderately sorted sands with occasional granulitic inclusions. As discussed below, this unit has been dated to 412 ± 87 ka and is very weakly bedded with root voids and small burrows throughout, which have been infilled with coarser dark brown sand. Near the contact with the overlying lake sediments, they comprise poorly sorted, very fine red-brown sands with occasional marl laminae fragments horizontally embedded within, and numerous dark brown infilled root voids. The sharp contact with the overlying lake sediments is laterally variable and moderately diffuse, due to uneven bedding.

The overlying lake sediments are split into two units. The first comprises of a series of finely laminated and iron-indurated carbonate-rich marls, capped by desiccation cracks. The second unit consists of interdigitated sand and fine-grained carbonate rich marl beds. The marl beds no longer preserve laminations and instead are massive and bioturbated, are increasingly ferrunginous, and contain evidence of manganese staining. Lake sediments become increasingly blocky and friable towards the surface of the sequence, likely reflecting post-depositional weathering. High levels of iron may reflect increased redox within lake waters, possibly resulting from seasonal lake level variations and the stratification of iron oxide in anoxic and/or highly vegetated (i.e. shallow) waters[67], which is typically accelerated by higher lake temperatures, but also can be related to post-depositional diagenesis via repeated incursions of groundwater impacting the sediments.

3.2 Northeastern Lake

This relict lake deposit is a large preserved linear ridge, extending N-S across the basin floor (Supplementary figures 2-5). The west face of the ridge is steeply sloping with inclined beds exposed as a sharp face towards the dune face of the enclosing barchan. The east face of the ridge gently slopes to the west with lakebeds slumping and eroding out downslope towards the basin floor. Two such beds form weakly defined terraces on the western slope and run along the extent of the ridge. Sediments eroded from the southern tip of the ridge are underlain by slope material eroded from the eastern extent of Central Lake, and overlain by the western extent of Southwest Lake. This surface is inclined 20° towards the ridge apex and

comprises weathered and indurated marl bed fragments intermixed with surficial aeolian sand.

The Northeastern Lake represents the second phase of lake formation at KAM-4. Beneath the fine-grained carbonate-rich marl sediments are pale cream-grey medium to coarse sands with weak bedding structures, root voids, and iron staining throughout. These have been dated to 337 ± 39 ka and become more cemented with depth, displaying a sharp contact with the overlying lake sediments, which are dated to 306 ± 47 ka. The lacustrine phase at Northeastern Lake is characterised by interstratified beds of fine-grained carbonate rich marls with iron and manganese staining, though this is much less than at the Central Lake, and occasional sand lenses between bedding planes. The presence of sand lenses suggests phases of lowered water levels at the site. Numerous single rootholes (likely indicative of grasses) occur in association with iron and manganese-rich layers, while there is also a notable variability in the thickness of the marl beds, which are finer and more friable near the onset and termination of lake sedimentation. The surficial bed displays a high degree of vegetative evidence (up to 1 cm root voids) and preserved crayfish burrows. The surface of the upper beds are inclined 19° towards the western bounding dune face. Stratigraphic evidence for the transition from drier/arid to more humid conditions at Northeastern Lake is similar to that described for Central Lake and has been observed elsewhere as a common feature of palaeolake sequences in Arabia[18,19,64].

3.3 Northwestern Lake

This relict lake outcrop is a raised circular feature (similar in form to Southeast Lake), which lies adjacent to the northern extent of the Northeast Lake ridge (Supplementary Figures 2,4,5). The exposed bed features a basinal structure, indicative of its shape prior to desiccation, and is capped by heavily weathered and indurated dark grey marl fragments, which erode out downslope along all sides. Surficial sediments are moderately granulitic and contain large numbers of root voids and crayfish burrow remains. The preserved lakebed is situated at the northern end of the wider basin, and partially overlies talus material from the northern tip of the Northeast Lake ridge, where both relict lake sequences are at their shallowest.

The thickest relict lake sequence at KAM-4 is underlain by reddish-green medium grained sands featuring root voids, iron staining and occasional rhizoliths (Supplementary Figures 5-8). These progress into sandy marls featuring vertically pervasive root voids, becoming more massive with a lower sand component up sequence. Unlike other lake sequences at KAM-4, marl formation at Northwestern Lake is truncated by a thick unit of darker, Fe-stained, calcareous silt-sands featuring rip-up marl fragments (from the underlying marl layer) and occasional small gravel clasts. These are generally loose and friable with occasional marl laminae, and contain lithics and fossils (discussed below). The overlying carbonate-rich marl units are similar to those nearer the base of the sequence but are generally more massive, featuring mollusc/gastropod remains, and with a sharp contact with the underlying unit and a diffuse contact with the marls above. These become increasingly blocky, finely laminated and friable towards the top of the sequence, which comprises heavily weathered carbonate-rich marls, mid grey in colour with sand lenses, manganese staining and numerous pin hole root voids.

Phases of massive marl formation at Northwestern Lake likely reflect a continuous depositional phase (i.e. wetter conditions and a deeper water body), while iron-stained silty-sands featuring evidence for vegetative processes and occasional (e.g. seasonal) drying reflect a lowering of lake waters and a palustrine depositional setting at the site. The presence of lithic material and fossil remains within such sediments in the middle of the section, reflect human and faunal occupation during a phase of lake contraction that was nonetheless capable of supporting ecological communities.

As it preserves the thickest marl beds, has the largest fossil assemblage from the site, and a surface distribution of lithics suggesting that there was a good chance of recovering buried lithics, we selected the Northwest Lake for a series of excavations, consisting of four 2 x 2 metre trenches (Supplementary Figure 5). This helped to clarify the stratigraphic sequence and site formation processes. The lacustrine deposits slope steeply (Supplementary figure 6) over the underlying sand topography. From the surface topography and from the stratigraphy of trenches 1 and 3, it is clear that a sand bar existed on the eastern side of the Northwest Lake. This would have provided the easiest access to the lake for animals, opposite the steep slope on the northwest side. Concentrations of animal remains are focused around this spit of sand, where they were subsequently covered by further lake deposits, and downslope where they have been eroded and scattered westwards across the lake surface. Fossil remains were identified in a consistent position in trenches 1 and 3 (Supplementary Figures 6 and 7), as summarized by Stewart and colleagues[33]. Trenches 2 and 4 on the south side of the Northwest Lake revealed a similar stratigraphy, and here while no fossils were recovered here, six lithics were recovered in the same stratigraphic position as the fossils in the other trenches (Extended Figure 3).

3.4 South, Southwest, and Southeast Lakes

The southerly relict lakebeds within the KAM-4 basin (Supplementary Figures 3 and 5) form a series of circular outcrops raised above the interdunal floor and adjacent with the southern end of Northeastern Lake and eastern extent of Central Lake. The centre of the Southeast lake basin lies ~3 m below its outer edge. Tapering out towards the east, the surface material comprises dark grey weathered and indurated palustrine fragments with a profusion of root voids and crayfish burrows, indicative of shallow water/shoreline sedimentation. These sediments are less well preserved towards the centre of the basin.

The southerly palaeolake sequences at KAM-4 display broadly similar stratigraphic profiles (Extended figure 3), being underlain by pale cream moderately well sorted sands featuring root impressions and iron staining. At South Lake and Southeast Lake these underlying sands progress sharply into weakly laminated carbonate-rich marls, which become more massive up-sequence (although notably browner and bioturbated at Southeast Lake). Ages for the basal sands across all southerly lakes range from 184 ± 14 to 142 ± 13 ka (Extended Fig. 4). For Southwest Lake, the shift from aeolian to lacustrine conditions displays an extended sedimentary transition (c. 70-20 cm), featuring a progression from horizontally bedded sands through a diffuse contact into calcareous, iron-mottled, and bioturbated carbonate-rich silty-sands. These are dated to 143 ± 10 ka. A subsequent shift to full lacustrine conditions is marked by a progression from sandy into massive fine-grained carbonate rich marls, while the surficial capping of heavily indurated marls featuring crayfish burrows and root voids reflect a lowering of lake water levels (possibly marshy conditions) and subsequent termination of the lake.

4. JQ-1 and JSM-1 sites

The sites of Jebel Qattar 1 (JQ-1) and Jebel Umm Sanman 1 (JSM-1) were previously reported by Petraglia and colleagues [28,68], and subsequently included in other papers on lithic assemblages from Jubbah[9,66,69]. At JQ-1, an upper assemblage of lithics was found on a palaesol dated to 75 ± 5 ka, and shallowly buried by sands overlying this palaeosol to the south. A small lower assemblage of lithics was reported by Petraglia et al.[28], in association with an incipient palaeosol and an OSL age of 211 ± 16 ka. Most of the small collection of 28 lithics from the MIS 7 deposit were small flakes, but included a single clear Levallois flake and one probable Levallois flake. The assemblage hinted at a Middle Palaeolithic character, but was too small to be clear.

In 2013 renewed excavations were conducted at JQ-1 to enlarge the sample of lithics, in the form of two large trenches just west of the long strip trench previously excavated (trench 6). The new trenches were adjacent to each other at 28.014552 N, 41.06033 E, labelled trench A (15 x 10 metres) and B (6 x 6 metres). The multiple trenches previously dug across the site show that there is little significant stratigraphic variation in the sediments, which consist of flatly bedded deposits (see[28] for stratigraphic information) and lithics in a consistent stratigraphic position. The stratigraphy and dating of the new trenches follow that reported by Petraglia and colleagues[28] so no further studies of stratigraphy or chronology have been conducted (see extended figure 3 for stratigraphy, and supplementary figure 15 for site photos).

JSM-1 is located at the western end of the Jubbah Basin, just south of a seam-like source of a distinctive yellow raw material – described as argillaceous sandstone/quartzite by Groucutt and colleagues[66]. Petraglia and colleagues[28] reported lithics across the surface at the site. Test excavations rapidly hit bedrock, and OSL dating of these shallow sediments produced inconclusive results of between 140 and 40 ka, and most probably ~100-60 ka, in sediments which were interpreted as showing bioturbation. A small assemblage of 88 artefacts from the surface and the excavations suggested a focus on centripetal Levallois technology.

Renewed excavations were conducted at JSM-1 in 2013, with four new trenches located ~ ten metres west of the earlier ones. None of the new trenches were bottomed, despite reaching depths of between 1.5 and 2.5 metres. We attribute this difference compared to the test trenches to the presence of a palaeo-gully in the area of the new trenches, which subsequently filled with sediment. The trenches produced a consistent stratigraphic sequence (Extended figure 3). In summary, the deposits formed by a combination of colluvial movement from the adjacent jebel and aeolian sands. Trench 3 was sampled for OSL dating, and the following stratigraphic description is based on this trench, but the sequence is the same across the trenches (Supplementary figures 16 and 17).

Context 001A was a loose to moderately compact pale orangey brown gravelly silty sand. The silt formed a thin crust underneath the surface gravel and sand and was probably deposited during a sheetwash event and then sun-baked to form a crust. The sand was aeolian in origin while the gravel and probably the lithics were a locally derived colluvium. Context 001B was a moderately compact pale brownish orange sand with frequent small quartz pebbles and angular pieces of sandstone and occasional lithics. A degree of rodent burrowing was observed in this layer. Context 001C is a moderately compact pale brownish orange

gravelly sand. The gravel is composed of small to medium clasts of sandstone and quartz up to 2.5 cm in maximum dimension. Context 001D is a moderately compact pale brownish orange sand with occasional small pebbles of quartz and sandstone and occasional lithics. Some burrowing was evident in this layer. The orange sand that dominates Unit 001 is likely aeolian in origin. Context 001C may represent a more stable surface in this aeolian sequence on which colluvial gravels and lithics accumulated.

Context 002A was a moderately compact pale yellowish white silty sand with occasional rhizoliths, moderate small to medium clasts of sandstone and quartz some of which were encrusted with carbonate, and lithics. The carbonate crusts and rhizoliths indicate that unit 002 represents wetter conditions than unit 001. Context 002B was a moderately compact pale greyish yellow silty sand with moderate small to medium clasts of sandstone, some small carbonate nodules and occasional lithics. Context 002C was a moderately compact pale brownish grey silty sand with occasional small pebbles and carbonate nodules, but no artefacts.

Unit 003 was a moderately compact pale brownish white gravelly sand with frequent lithics and carbonate crusts on some of the clasts. The gravel is composed of small to large clasts of diverging lithologies including quartz, sandstone and ferruginous sandstone (all of which are available up slope on the jebel). There was considerable horizontal variation in Unit 003, both within and between trenches and the unit was subdivided into separate contexts where it was thicker in the eastern trenches 3 and 6. Where there is a higher density of clasts the sand has been stained orange from iron leaching out of the ferruginous sandstone.

Context 004 was a moderately compact pale yellowish white sand, with moderate small pebbles that gradually decreased in frequency with depth. This context was not bottomed in any of the trenches. This context seems to be archaeologically sterile, with the only lithics found in the very upper part and possibly relating to the overlying sediments.

Three OSL samples from Trench 3 were analysed (see following section). Given the extensive bioturbation evidence in the Petraglia and colleagues[28] samples from the shallow trenches 1 and 2 (<50 cm), we focussed here on taking deeper samples (> 80 cm).
To briefly summarize these results, the deepest sample JSM-OSL12 from Unit 4 most likely dates to ca. 130 ka. JSM-OSL9 and JSM-OSL7 from the overlying units 3A and 2C respectively contain one population of grains dating to around 130 ka and another to around 75 ka. It may therefore be that the sequence records a series of occupations throughout MIS 5, or, alternatively, that the site represents a late MIS 5 (5a) occupation, with mixture of underlying MIS 5e sediments into the deposits. Given these two possibilities, we treat the lithic assemblage as a single MIS 5 assemblage. Future work at the site giving enlarged sample sizes may be able to subdivide different phases of MIS 5. No differences in raw materials, weathering (beyond buried lithics generally being fresh, those on the surface showing some weathering) or technology were observed between the lithics from the different units and the surface. More than 70% of the excavated JSM-1 lithics are from the upper deposits (units 1 and 2), which be assigned to the MIS 5a, with a further are from 26% to unit 3 which seems to represent the interface between the early and late MIS 5 deposits (see OSL section), and only 4% were found in the lower (MIS 5e) unit 4. Our working model is therefore that the JSM-1 assemblage represents either exclusively or at least primarily a late MIS 5 assemblage dating to around 75 ka (described in SI 8).

5. Luminescence dating of KAM-4 and JSM-1

5.1 Luminescence dating methodology

Luminescence dating is a group of dating techniques that allow determination of the time elapsed since sediment was last exposed to sunlight. Luminescence techniques are usually applied to sedimentary quartz (optically stimulated luminescence, OSL) and feldspar (infrared stimulated luminescence, IRSL), and have been applied successfully to a wide range of archaeological and Quaternary deposits[e.g.70-72].

After burial the sediment is exposed to a low level of radiation coming from both cosmic radiation (cosmic rays) and the decay of naturally-occurring radionuclides, principally uranium (U), thorium (Th), rubidium (Rb) and potassium (K). These radioisotopes, which are present in the surrounding sediment, emit alpha particles (α), beta particles (β) and gamma rays (γ). Most mineral crystals contain lattice defects or impurities where electrons become trapped when excited by ionizing radiation. The crystals store energy at a constant rate with the amount of stored energy being related to the time since deposition. Electrons can be removed from traps when a small amount of stimulating energy is subsequently applied, either from sunlight during transport or from an external light or thermal source, and this reduces the trapped electron population, a process referred to as bleaching, zeroing or resetting of the signal. In the case of OSL and IRSL, photons are used to release the electrons from the traps. Once the electrons are evicted, they can be trapped again, or recombine in defects attractive to electrons (luminescence centres). The recombination of the electrons result in the emission of light, termed OSL or IRSL. The intensity of the light signal emitted is proportional to the amount of electrons stored in the defects. This allows for the stored charge to be quantified. It can be used in combination with the data on the total dose rate received while buried (Gy/ka) to calculate the age since the last light or heat exposure following the equation[73]:

Age (ka) = Equivalent dose (D_e) (Gy) / Dose rate (Gy/ka)

The resulting luminescence age is obtained in calendar years prior to measurement – the datum used in this study is 2018.

Feldspar and quartz are routinely used for luminescence dating. Quartz is generally the dosimeter of choice for dating thanks to the stability of its stored charge. Feldspar luminescence bleaches both more slowly and less completely[74-76]. However, feldspar produces brighter signals and has the ability to absorb higher doses of radiation without saturation (thereby extending the useable age range of the technique); in addition, the internal dose rate of potassium-feldspars (K-feldspars) reduces the impact of variations in the external dose rate (e.g. due to uranium mobility) on the final age calculation[77]. A significant barrier to the use of feldspar, however, is the instability of the stored signal measured by standard infrared stimulation at low temperatures (IRSL, 50°C). Known as ‘anomalous fading’[78-79], progressive loss of this signal can cause significant age underestimation[80].

5.2 Sample collection and preparation

At JSM-1, samples were collected from trench 3, approximately ten metres west of the trenches reported by Petraglia and colleagues[28] (Extended Figure 3; Supplementary Figure 17).

At KAM-4, samples were collected from the aeolian sands underlying marls for each lake phase and, where possible, sand rich portions within the overlying marls (Extended Figure 3; Supplementary Figure 17). Samples KAM4-OSL1-6 were taken during the 2013 field season, while samples PD7-14 were collected in 2017. Aeolian sands were sampled by driving opaque plastic tubes into clean section faces, whereas marl samples were taken as consolidated blocks.

Samples coded “KAM4” were measured at the Research Laboratory for Archaeology and the History of Art, University of Oxford, while samples coded “PD” were measured at the Royal Holloway Luminescence Laboratory, Royal Holloway University of London. In both cases, samples were prepared under subdued red-light conditions. The outer, light-exposed portions of each sample were removed and used for environmental dose rate measurements and estimation of the sample’s moisture content. The remaining sediment was treated with hydrochloric acid (1M HCl) and hydrogen peroxide (H_2O_2) to remove carbonate and organic matter respectively. The samples were wet sieved, and where sand-sized (>62.5 μm) material was present, feldspar (KAM4 samples) or quartz (PD and JSM samples) was extracted. Quartz was extracted via density separation at 2.62 and 2.70 g/cm^3, and a subsequent HF acid etch (23M HF for 60 mins, followed by a 10M HCl rinse). Etched material was re-sieved to remove partially dissolved grains. K-feldspars were extracted using density separation at 2.58 g/cm^3. Where marl samples contained negligible sand-sized material, the 4-11 μm fraction was isolated by Stokes settling, and quartz was purified by prolonged (~1 week) immersion in silica-saturated fluorosilicic acid (H_2SiF_6.), followed immersion in 10M HCl overnight.

5.3 Luminescence measurements

Equipment

Luminescence measurements were performed using Risø TL/OSL-DA-15 or TL/OSL-DA-20 instruments, with the former system being fitted with a single-grain OSL attachment[81]. Stimulation of multi-grain aliquots was carried out using a blue (470 nm) light emitting diode (LED) array (nominal power density 33 mW/cm^2) and an infrared (875 nm) LED array (nominal power density 135 mW/cm^2), while single-grains were stimulated using a 10 mW Nd: YVO4 solid-state diode-pumped green laser (532 nm) focussed to yield a nominal power density of 50 W/cm^2 [82]. Quartz luminescence emissions were detected via Hoya U-340 filters (2.5 mm thickness for single-grain measurements or 7.5 mm thickness for multi-grain measurements) while a combination of 2 mm Schott BG-39 and 3 mm Schott BG-3 filters or Schott BG-39 and Corning 7-59 filters were used to detect feldspar signals. Luminescence signals were measured using an Electron Tubes Ltd 9235QB photomultiplier tube. All irradiations were performed using a 1.48 GBq $^{90}Sr/^{90}Y$ beta source, calibrated relative to the National physical Laboratory, Teddington ^{60}Co gamma-source (Hotspot 800) [83]. For single-

grain measurements, the effects of source heterogeneity[84] were circumvented by applying a grain position correction to each equivalent dose following Armitage and colleagues[22]. Position corrected equivalent doses were used in all single-grain age calculations.

5.4 Equivalent dose determination

For quartz, equivalent dose (D_e) determinations were carried out using single-aliquot regenerative-dose protocol (SAR[85,86]; supplementary table 4). Regeneration doses were chosen to bracket the expected palaeodose. A number of additional regeneration points were also included to monitor the quality of the data generated: (1) a zero dose point to measure the recuperation; (2) a repeat measurement of the initial regeneration dose to calculate the recycling ratio[85]; (3) a second repeat of the initial regeneration dose followed by a room temperature IR bleach and subsequent OSL measurement to calculate the IR depletion ratio[87]. Optimal preheating conditions were determined using dose recovery preheat plateau tests[86]. Several preheating regimes yielded measured: given dose ratios 2σ of unity for all samples, demonstrating the overall robustness of the SAR method. We adopted a combination of 260 °C for 10 s for preheat 1 (PH1, the preheat before measurement of the natural or regenerated luminescence intensity) and 220 °C for 10 s for PH2 (the preheat before measurement of the test dose luminescence intensity) for single-grain measurements and a 240 °C, 10 s PH1 followed by a 160 °C, 0 s PH2 for single aliquots measurements (Supplementary Figure 18). The dose response curves were fitted with a saturating exponential, or saturating exponential plus linear function, chosen for each individual curve on the basis of goodness of fit. Most of the quartz grains and the aliquots measured are dominated by the fast OSL component, for which the SAR procedure was developed[85]. The D_e values for grains/aliquots were calculated by projecting the sensitivity-corrected natural luminescence intensity (L_n/T_n) onto the dose response curve, with an uncertainty term determined using a 100 iteration Monte Carlo simulation. Curve fitting, D_e determination and Monte Carlo simulation were performed using version 4.31.9 of the Luminescence Analyst software[88].

Multigrain aliquots of individual grains were rejected where: (1) the natural signal from the aliquot/grain could not be distinguished from the background signal (determined using Luminescence Analyst “sig. >3 sigma above BG” rejection criterion); (2) the recycling ratio differed from unity by >20 %; (3) the sensitivity-corrected zero dose luminescence intensity was >5 % of the natural luminescence intensity; 4) the IR-depletion ratio was more than two standard errors below unity (quartz only); (5) the uncertainty on De exceeded 30 %; (6) the natural luminescence signal (L_n/T_n) intercepts the dose response curve at a point where growth has ceased. In addition, grains from samples coded “JSM” were rejected where D_e was consistent with zero at two standard errors. This additional criterion followed established practice at the site[68] and was added to exclude modern grains incorporated during sampling.Details of accepted and rejected aliquots and grains are presented in supplementary table 5. For single grain datasets only 1.0 to 1.6 % of the measured grains had acceptable luminescence characteristics (see supplementary Table 5).

Feldspars were measured using post-infrared, infrared stimulated luminescence (pIRIR) procedures (Table supplementary table 4). These measurement procedures are intended to remove the fading component of the signal via a high temperature IR stimulation, prior to one or more elevated-temperature IR measurement(s). The pIRIR signals can then be used to calculate the age of the sample[89-91]. Samples with codes beginning ‘KAM4’ were measured using the $pIRIR_{290}$ measurement sequence, while PD samples were measured using the $pIRIR_{225}$ measurement sequence (Table S1). Net $pIRIR_{225}$ and $pIRIR_{290}$ signals were

calculated by subtracting a mean background (calculated from the signal observed during the last 50 s of stimulation) from the total signal emitted over the first 2 s[92]. For the PD samples, fading rate was measured following Huntley and Lamothe[77] and Auclair and colleague[93] using Analyst 4.31.9[88], yielding a g-value 0.87 ± 1.01 % ($pIRIR_{225}$). This g-value is similar to reported values for quartz and may indicate the absence of fading[94-96]. Nonetheless, K-feldspar ages were corrected for fading, though the low fading rate means that this correction is small.

Although they exhibit limited fading, the $pIRIR_{225}$ and $pIRIR_{290}$ signals are reduced to zero much more slowly than quartz OSL or lower temperature (e.g. IR_{50}) K-feldspar emissions. Consequently, it is possible that these signals may yield age overestimates due to the presence of a "residual" or "unbleached" signal in K-feldspar at the point of burial. To test this possibility, K-feldspar from a modern dune sample (PD19) taken from KAM-4 were measured using the $pIRIR_{225}$ and $pIRIR_{290}$ measurement procedures detailed in Supplementary Table 4. This analysis yielded residual doses of 1±0.2 Gy for $pIRIR_{225}$ and 2.5±0.6 Gy for $pIRIR_{290}$ (1σ, n=6 in both cases). Since PD19 is believed to be analogous to the material incorporated into ancient samples from KAM-4 and elsewhere in the Nefud Desert at the time of burial, pIRIR ages in this study were not corrected for the presence of an unbleached residual dose.

5.5 Burial dose determination (D_b)

The statistical models most frequently used to extract a single burial dose (D_b) from the distribution of measured D_e values are the central age model (CAM[74,97]), the Minimum Age Model (MAM[98]), and the Finite Mixture Model (FMM[99]). The different models use different assumptions about the dataset so it is crucial to choose the correct one. CAM is designed for well-bleached samples which have experienced no post-depositional mixing. This model represents the ideal case, and calculates D_b from a weighted mean of the measured D_es. The MAM, as applied to single grain datasets, is used when the sample contains both fully and partially bleached grains. MAM fits a truncated normal distribution to log D_e values, with the truncation point giving D_b. Finally, FMM was developed to identify separate, fully bleached populations of grains within a single-grain dataset. This model is used when the sample is a mixture of different grain population, for example when mixing occurred after burial.

After the analysis of the degree of skewness, the kurtosis and the overdispersion of the D_e distributions following Bailey and Arnold[100], CAM was considered the most appropriate statistical model to use with our single aliquot datasets. Because of the presence of low-dose (presumably intrusive[28]) grains within the single-grain datasets the FMM was used to identify the discrete grain populations for the JSM samples. The FMM was fitted by maximum likelihood using overdispersion values of 10-15%. The optimal fits were determined from the maximum log-likelihood values and the Bayesian information criterion[101].

5.6 Dose rate determination

The total dose rate of a sample is a combination of alpha, beta, gamma radiation, and a cosmic ray component.

Beta dose rates on the JSM and PD samples were measured using a Risø GM-25-5 low-level beta counting system[102], using MgO and Volkagem loess standards[103]. External beta dose rates for the KAM4 samples were calculated from the concentrations of potassium, thorium,

and uranium measured via ICP-MS analysis (Activation Laboratories, Ltd., Canada) using the conversion factors of Adamiec and Aitken[104]. The alpha doses for these samples were calculated for a spherical grain (average 217.5 μm diameter) using uranium and thorium alpha attenuation factors from Brennan and colleagues[105].

Gamma dose rates for PD samples were measured in the field using an EG&G Ortec digiDart-LF gamma-spectrometer, while gamma dose rates for JSM and five of the KAM4-OSL prefixed samples were measured using an Inspector 1000 gamma spectrometer. In all cases, the data were analysed using the threshold technique[106,107]. KAM4-OSL6 was collected from a thin sediment layer (7 cm deep) bracketed by consolidated carbonate-rich sediments, preventing the use of a gamma spectrometer. The gamma dose rate for this sample was modelled assuming an infinite planar geometry[80] comprising three layers (the sampled sediment, upper and lower marls) each of which were assumed to be homogenous with respect to gamma dose. Gamma dose rates were calculated using ICP-MS radioisotope concentration determinations performed on representative samples of each layer. The marls were assumed to have a density of 1.36 g.cm^{-3}, while a density of 1.74 g.cm^{-3} was used for the sediment. The fractional dose rates for potassium, thorium, and uranium from each sediment layer[80] were then integrated for all horizontal planes of the tube sample, and an average gamma dose rate was calculated by volume scaling. The final gamma dose rate was assigned a relative standard error of 5% in order to be consistent with the treatment of gamma spectrometer measurements. K-feldspars also have an internal dose rate. This dose rate was calculated assuming grains contain 12.5 ± 0.5% potassium[108] and 400 ± 100 ppm rubidium-87[109]. For potassium, dose rates were calculated using absorption factors from Brennan[110] and the conversion factors of Adamiec and Aitken[104]. The absorbed dose due to rubidium was taken from Readhead[111].

Dose rates were corrected for: (i) alpha efficiency (0.15 ± 0.05 for sand-sized feldspars[112] and 0.038 ± 0.002 for 4 – 11 μm quartz[113]); (ii) attenuation due to grain size for quartz[114] and for feldspars[110]); (iii) loss of beta dosed material due to HF etching[114] and (iv) moisture content[80]. A mean burial water content of 5 ± 2.5 % was assumed for all samples. The 2σ uncertainty on water content encompasses completely dry conditions (0%) and saturation for 25% of the burial period (10%), representing the full range of reasonable mean water content scenarios for these samples.

Cosmic dose rates were calculated using site location and present-day burial depths[115]. For the PD samples taken from sands underlying marl (all samples apart from PD10 and PD14), the overburden consists of between 25 – 30 cm of sand containing 5% water by mass (1.74 g/cm^3), with the remainder of the overburden depth being carbonate material containing 5% water by mass with assumed bulk density of 1.36 g/cm^3. The latter bulk density is based on measurements of carbonate material at Al Wusta[20]. For samples from the carbonate material, an overburden density of 1.36 g/cm^3 was assumed throughout the overburden. Using these overburden densities and the present-day burial depth, cosmic rays contribute between 18 to 38% of the total environmental dose rate. Increases and decreases in the overburden due to sediment deposition (e.g. lake sedimentation, movement of dunes) and exhumation linked with erosional processes is common, and may cause important variations of cosmic dose rate received by the sample over time[e.g 116,117].The dynamics of western Nefud dunes is poorly understood. Sparse vegetation and a thick lag of coarse sand protects dunes from deflation, limiting their mobility[26] and leading several studies to classify Nefud dunes as inactive[65,118,119]. Nevertheless, many palaeolake deposits have a morphology which implies topographic constraint by sand bodies which are no longer present and some are encroached upon by dune

slipfaces, suggesting that some dune migration has occurred since lake desiccation. The sensitivity of our ages to episodic burial under migrating dunes was tested by calculating cosmic dose rates with and without an additional 15 m depth of sand. The additional overburden reduced the calculated age by ~13 ± 3 %, with the majority of recalculated ages (10 out of 15 samples) remaining within the 1σ uncertainties of the age calculated using present-day burial depth. Given the relatively low sensitivity of our ages to changes in overburden density, and in the absence of compelling evidence that the sampled sediments were buried under migrating dunes for an appreciable period of time, present day overburden has been used to calculate cosmic dose rates.

5.7 RESULTS

Supplementary Tables 6 and 7 present dose rate and age data, while D_e distributions are presented in supplementary figures 20a-c.

Most samples have overdispersion values (the relative spread of equivalent doses after measurement uncertainties are excluded) which are higher than that expected for well-bleached, unmixed sediments (~20 % for quartz). The material measured from all sites is assumed to be aeolian in origin, and conventionally in a dryland environment this material would be assumed to be well-bleached prior to deposition, suggesting that partial bleaching is not the cause of high overdispersion values. At JSM-1, elevated overdispersion caused by both high and low dose grains may be explained by bioturbation. Burrows were identified during sampling on the JSM-1 site, and bioturbation may be common in the unconsolidated sands underlying lake deposits throughout the Nefud Desert, since evidence of burrowing is commonly observed. The presence of high dose grains in the dated layers may be due to the spatial variability of beta emitters such as zircons and K-feldspars[120], which alone may be responsible for overdispersion values as high as ~25%[121].

Overdispersion in the single-grain feldspar datasets is also high (up to 74%). In addition to the factors described above, feldspar overdispersion could also be caused by variation in internal dose rate and fading rate, though the latter should be low due to the use of pIRIR measurement procedures. Feldspar was not abundant in the KAM4 samples, so it was only possible to measure 10-15 aliquots per sample Supplementary Table 5).

5.8 INTERPRETATION

Jebel Umm Sanman (JSM-1)

JSM-OSL7 has two D_e components, yielding ages of 77.6 ± 6.7 ka (67 % of grains) and 136.1 ± 14.2 ka (33 % of grains). JSM-OSL9 has also two D_e components, corresponding to burial ages of 71.5 ± 6.4 ka (43 % of grains) and 123.7 ± 10.2 ka (54 % of grains). JSM-OSL12 has a clear dominant component containing 90% of grains and yielding an age of 129.9 ± 9.5 ka. The remaining 10 % of measured grains belong to a component giving an age of 48.5 ± 7.6 ka. These grains are potentially intrusive, and are not considered further. The data for JSM-OSL7 and 9 are both interpreted as indicating the mixture of two sediment bodies with ages of ~75 ka and ~130 ka. JSM-OSL12 is interpreted as containing only grains from the older sediment body, and the layer from which OSL12 was extracted may represent the source of older material in OSL7 and 9. Consequently, we interpret OSL12 to have an age of 129.9 ± 9.5 ka, while OSL7 and 9 were probably deposited around ~75 ka, but were mixed with the older underlying sediment subsequent to deposition, probably via bioturbation.

Khall Amayshan 4 (KAM-4)

The marls associated with the Central Lake are heavily iron indurated, implying that they are the oldest deposit in the KAM-4 basin, as does their partial covering by younger dated deposits, discussed below. The sample taken from the sand underlying Central Lake (KAM4-OSL1) yields an age of 412 ± 87 ka, with the large uncertainty term reflecting relatively high overdispersion (55%). The 1σ age range of 326 – 499 ka precludes definitive attribution to a MIS, but is centred on MIS 11, while the overlying Northeast Lake is dated to MIS 9 (see below). Furthermore, this sample was taken from sands underling the marl, and is therefore a maximum age. Using these lines of evidence, we infer that the marl overlying KAM4-OSL1 was deposited during an MIS 11 humid phase. Samples for the Northeast Lake come from a sand wedge within the marl deposit (KAM4-OSL3, 306 ± 47 ka) and from the underlying sand (KAM4-OSL2, 337 ± 39 ka), indicating that this lake formed during MIS 9.

The existence of lakes at KAM 4 during MIS 11 and MIS 9 is consistent with data from elsewhere in the Nefud Desert. Notably, Rosenberg and colleagues[18] report three directly dated sites (their Sites 10.2, 16.1 and 13.5) along with another 11 sites with maximum ages obtained by dating sands underlying palaeolake sediments, which imply humid phases during MIS 9 and MIS 11. However, the use of dates obtained from sands underlying a palaeolake to infer the age of the lake itself is potentially misleading. In some instances, dates obtained for sands underlying and overlying a marl are in good agreement, suggesting that humidity occurred relatively quickly after deposition of the former[20]. However, this is not always the case, and some sands underlying lake marls are substantially older (>100 ka) than the lake sediments themselves[56].

Two samples (KAM4-OSL6 and PD13) from the sands underlying Northwest Lake yielded different ages (80.4 ± 20.6 ka and 141 ± 13 ka respectively). Four ages were calculated for the lake sediments themselves. Three sub-samples of PD14 were measured, yielding ages of 194 ± 13 ka (60-90 μm quartz), 210 ± 16 ka (4-11 μm quartz) and 192 ± 20 ka, (60-90 μm K-feldspar fading corrected $pIRIR_{225}$). KAM4-OSL5 yielded an age of 189.8 ± 36.2 ka (fading corrected $pIRIR_{290}$). Since all ages from the Northwest Lake sediments are in good agreement, despite the range of minerals, grain sizes and measurement techniques used, we are confident that this lake formed at ~195 ka (MIS 7). This age is also consistent with a U-series estimate discussed in the subsequent section. However, the ~195 ka age for Northwest Lake is considerably older than those for the underlying sands (80.4 ± 20.6 ka and 141 ± 13 ka). This discrepancy may relate to:

(1) The introduction of younger grains into the unconsolidated sands from which samples PD13 and KAM4-OSL6 were taken, possibly due to bioturbation. However, care was taken to avoid visible burrows/roots during sampling, and PD13 was taken from the base of an excavation some way from the margins of the lake deposit, making bioturbation by burrowing unlikely.
(2) Problems with the dose rate determination, leading to an overestimation of the dose rate and therefore an underestimation of the age.

Irrespective of the cause, we regard the ages for samples PD14 and KAM4-OSL5 as more accurate than those for PD13 and KAM4-OSL6, and argue that the Northwest Lake formed at

~195 ka during MIS 7. Importantly, this is consistent with U-series dating of a fossil from Northwest Lake, discussed below.

Seven ages were produced from deposits associated with the South, Southeast and Southwest lakes, which appear to be broadly coeval on geomorphological grounds. KAM4-OSL4, PD11b and PD12, from sands underlying the Southeast Lake, yielded ages of 237 ± 22, 154 ± 11, and 149 ± 9 ka respectively, implying a maximum age of ~150 ka. A sample directly from wetland sediments from the Southwest Lake (PD10) yielded an age of 143 ± 10 ka (PD10), while the sand underlying this lake yielded an age of 184 ± 14 ka (PD9). Two samples from the sand underlying the South Lake gave ages of 168 ± 12 (PD7) and 142 ± 13 ka (PD8).

The single direct date on lacustrine material from the Southwest Lake yielded a 1σ age range of 133 – 153 ka which mainly falls within late MIS 6, though the upper boundary of the age lies close to the MIS 6/5e transition. The attribution of the Southwest Lake to MIS 6 would provide the first directly dated evidence of humidity in the western Nefud Desert during this period. Conversely, if the lake is of MIS 5e age then this is consistent with early MIS 5 ages from Alathar[56], KAM1, and Site 16.5[18]. It is important to note that all of the ages for underlying sands from the South and Southeast Lakes are either indistinguishable from this direct age (PD11b, PD12, PD7 and PD8) or older (KAM4-OSL4 and PD9), making the dataset stratigraphically consistent. As only maximum (underlying) ages for the Southeast and South lakes are available, the lakes could possibly date to a subsequent period in the Late Pleistocene. As discussed above, the lithic assemblages suggest an MIS 5 age for Southeast Lake and MIS 3 age for South Lake. This can be tested further by future direct dating of the marl layers for these phases.

6. KAM-4 U-series dating

We used U-series geochronology to date a fossil tooth (Taxon: Bovidae, specimen number: KAM16/85) excavated from Unit 3 at Northwest Lake of KAM-4 (Supplementary Figure 5 and extended Figure 3). We previously described fossils from the site[33]. Direct dating of teeth (and bone) using this method relies on the fact that fresh tissues contain little or no uranium (U). Following burial, teeth exhibit open system behaviour and U may be taken-up via apatites that scavenge U but exclude Th. Radioactive decay of ^{238}U (via intermediate nuclides ^{234}U and ^{234}Th) produces ^{230}Th; the U-series age is then calculated by measuring the ratio of the daughter ^{230}Th to the parent ^{238}U. With a half-life of ca. 75 ka for ^{230}Th, the dating technique has an application range of ca. 500 kyr (e.g., see further details in Grün[122]). Because the starting concentration of U is effectively zero, in ideal situations, U-series methods commonly produce minimum age constraints for the fossils being dated. Where U is taken-up rapidly following burial and during fossilisation, the resulting age will be close to the true age of the specimen[123]. In contrast, if the U uptake occurs much later, then the apparent U-series age will be significantly younger than the true age of the fossil. However, because of the open system nature of teeth, U (which is highly soluble relative to Th) may in some cases be preferentially leached back into the burial environment thus effectively skewing the ratio of the daughter and parent isotopes. This will mean that the resulting age is not only unreliable, but in many cases, can lead to substantial overestimation of a fossil's true age[124]. There are many methods to test the reliability of a given U-series date of a tooth such as ^{230}Th age and U concentration profiling through the fossil to assess U uptake/loss behaviours, isotope ratio evolution plots, or comparison of U-series data to dates from independent geochronological techniques[e.g.122,125-128]. In this case, we use the latter two approaches including comparison of the U-series age results to independent data from OSL (as previously described).

We produced two U-series ages for KAM16/85, targeting enamel and dentine, respectively (Supplementary Table 8). Both of these dental tissues commonly experience different behaviours during and after initial U uptake and so it is necessary to measure them separately. Powdered samples of both enamel and dentine were drilled from the tooth at Griffith University, Nathan, Australia. The powders were then chemically processed prior to being isotopically measured using a Nu Plasma HR multi-collector inductively coupled plasma mass spectrometer at The University of Queensland, Brisbane, Australia. We followed the sample preparation and measurement protocols as described in Zhou and colleagues[129].

Both dated components of the KAM16/85 tooth contain relatively high concentrations of U (ca. 4 ppm for enamel and ca. 18 ppm for dentine). An isotopic evolution plot shows that the dentine exhibits strong excess ^{230}Th relative to U (supplementary figure 21). This demonstrates that U has been lost from the system and thus a U-series age could not be calculated. In contrast, the enamel shows no obvious anomalies on the evolution plot (supplementary figure 21), and therefore appears to be reliable and yields a finite age of 204.8 ± 1.5 ka (2-σ uncertainties; Supplementary Table 8). Significantly, this date is remarkably consistent with luminescence burial ages of ~200 ka for the lake sediments of

KAM-4 NW Lake (extended Figure 3). This suggests that the tooth experienced early uptake of U and, although a minimum age, may be close to the true age of the deposit.

We also considered the possibility of electron spin resonance dating (ESR) of the tooth, a method that in combination with U-series, can provide even more reliable estimates of a true age of a fossil[130]. However, the U leaching observed in the dentine effectively precludes the use of the US model defined by Grün and colleages[131]. Additionally, such high concentration of U in the enamel (>2 ppm) is known to typically induce a massive internal dose rate overestimation. Together, these features make the tooth unsuitable for the technique (see[132]). This is an issue that has been frequently observed at localities in the western Nefud Desert, such as Ti's al Ghadah[60] and Alathar[56].

7. KAM-4 lithic assemblages

Lithic artefacts are found across the KAM-4 site (Extended figures 2-8, supplementary figures 9-13, 22, 23, 25, 28-34). As outlined below, their spatial patterning in association with distinct dated sediment deposits, as well as consistent differences in technology and weathering, indicate that multiple distinct assemblages of different ages are present at the site. These assemblages are given lettered names, from Assemblage A to Assemblage E. These assemblages are summarised in supplementary table 9. An example of how the lithics can be divided into assemblages which display little mixing is the distribution of handaxes (Supplementary figure 9). These are only found only found in Assemblages A and B, associated with the Central and Northeast Lakes. The single exception was a handaxe found in the middle of the Southeast Lake, probably as the result of being recently moved. It is quite possible that occasional lithics represent such movements, but on the whole the different assemblages appear discreet. In the same regard, bioturbation, landscape scavenging, wind transport, etc. can introduce out of context lithics into stratified archaeological contexts.

The lithic assemblages have varied spatial patterning (Figure 1), in part reflecting the age of the associated deposits. For example, only a relatively small area of Central Lake deposits is preserved and there is little indication of the original topography of the original lake. Assemblage A lithics are found fairly randomly scatted on the surface. The Northeast Lake is a ridge-like remnant of an obviously once relatively large lake. But here, with Assemblage B, there are in some cases seemingly distinct knapping scatters preserved, such as a single handaxe and multiple flakes at the far northern end of this deposit (Extended Figure 2). The Northwest lake shows a focus of lithics on the eastern and southern margins of the deposit (Assemblage C), and there seems to be more of a preserved topography of the original deposits in this case. For instance, as discussed above, in the eastern part of this lake a sand bar extends into the area of marl deposits. This is a genuine palaeotopographic feature, as excavations either side showed the lake sediments steeply sloping over the top of this sand bar. Fossils were found concentrated around this feature, which shows preserved small-scale features of the topography of the lakeshore. Some bones have moved downslope from the eastern and southern edges which would have been the accessible side of the lake (with a steep dune the other side). A similar pattern characterises the Southeast Lake. There is good topographic preservation here, and unlike the previously described deposits, this one is less steeply deflated around its margins (except on the north). Many of the Assemblage D lithics associated with the Southeast lake lithics are found in relatively tight groups, such as in the northeastern area of the deposit. Finally, Assemblage E lithics are found in a relatively dense scatter below a small remnant lacustrine deposit of the South lake.

Before describing these assemblages in individual detail, two factors are worth mentioning. Firstly, while a detailed refitting programme has not yet been attempted, some refits were rapidly found during analysis of the assemblage (Supplementary figure 25). For instance, a broken preferential Levallois flake and the core to which it refitted were found in Assemblage C. In the dense easternmost scatter of Assemblage D, a concentration of distinctive rhyolite lithics otherwise almost unknown from the site were found. One of these flakes refits to a Levallois core. It is a preparatory flake, made before a Levallois flake was produced (which was not found on the site). These refits indicate good stratigraphic integrity,

as well as on-site stone tool manufacture. For assemblages such as Assemblage B, many of the flakes are consistent with being biface thinning flakes. In other words, all assemblages indicate consistent patterns of behaviour, in which raw materials and partially reduced lithics were brought to the site, where they were further reduced. Many were left on site, and others were removed and transported to elsewhere in the landscape.

The second key factor to emphasise concerns raw material availability and characteristics in the area. More than a decade of research in the Nefud Desert has given us a good understanding of the distribution of lithic raw materials in the landscape, and the kinds of materials commonly found in archaeological sites. The dominant bedrock of the region consists of Palaeozoic sandstone. In certain layers, this ancient sandstone has undergone cementation with iron. Some of these rocks are very iron rich, and can be described as ferruginous quartzite. We have previously described these rocks in details from Jubbah[66]. These rocks are the main kind of stone used by Pleistocene knappers in the region. At KAM-4 this is the most common raw material. Fascinatingly, both different examples of this rock and different ages (leading to different weathering) produce a spectrum from highly iron-rich dark brown quartzite to pale sandstone. The older assemblages at KAM-4 are often quite highly weathered and much of the iron has been leached out, which can produce both pale coloured lithics and often a distinctive banding in the rocks (Supplementary figure 23). This is not however a simple/consistent process, probably because slightly different sources of rock were used, but more a matter of general tendencies. In the case of Southeast and South Lakes, however, this weathering differential is useful as there is clearly less weathering in Assemblage E (from the South Lake). This suggests an age difference between these assemblages, which, as we shall see, both only have underlying (MIS 6) maximum ages, and therefore suggests that the Southeast Lake is older than the South Lake.

The ferruginous quartzite has reasonable properties of conchoidal fracture, but is variable in quality and the extent of fracturing and inclusions. It occurs as broadly rectangular slabs in horizontally bedded exposures. We have found occasional examples of exposures of this rock within the Nefud Desert dunefield. In general, however, it occurs just outside the dunefield, or at elevated points (as at Jubbah). While the exact sources of the ferruginous quartzite that was the preferred lithic material at KAM-4 are unknown, it can be stated that this rock occurs close to the site in beds on the edges of the sandsea. For instance, a large exposure exists at WNEF16_12 (28.078 N, 39.174 N), 18 km west of KAM-4 and one of many such beds in the area. Examples of the rock were collected from here for knapping experiments. And one not very proficient knapper (HSG) was able to rapidly produce Levallois flakes (Supplementary figure 24). When fresh this material is sharp and shiny. With weathering it goes dull, and then eventually iron leaches out to produce a pale colour. Various beds of this rock are found all along the margins of the western Nefud Desert, some just over 10 km from KAM-4 (e.g. at 28.077 N, 39.246 E). Limited exposures have been identified within the sandsea, but they do exist. For example, at WNEF16_26 (28.182 N, 39.444 E) relatively low-quality ferruginous quartzite bedrock occurs. Associated lithics, including a handaxe, appear to have been made on this material. Close to KAM-4, there are ferruginous beds just over 2 km to the northwest of the site (28.044 N, 39.335 E). So, while the precise sources of the KAM-4 ferruginous quartzite/sandstone are currently unclear – and this material often varies considerably within a bed, as much as between them[66] – the key point is that such material was widespread in the close environs of the site.

The second relatively major category of raw materials, although much less common, consists of other forms of quartzite, in a more formal sense (e.g. metaquartzites). These occur in

various shades and consistencies – with orange and yellow forms being the most common (supplementary figure 22). We have not observed these in a clear bedrock setting within the Nefud Desert. They seem to occur in the context of ancient fluvial deposits. This had previously been hinted at the site of KM-2[25], further west in the Nefud, although from the limited bedrock exposure there it was felt possible that they were part of a bedrock conglomerate. Handaxes from KM-2 showed the kind of technology present in the Nefud (supplementary figure 26), but no chronometric age estimates are available for the site. More recently, the site of WNEF16_4 was identified (27.899 N, 39.004 E). While this particular example is located 37 km west of KAM-4, it shows the existence of ancient rivers within the Nefud sandsea. The fluvial deposits here contain cobbles up to football sized and bigger. While mostly sandstone, quartzite clasts are also present.

Supplementary figure 13 shows the distribution of 'exotic raw materials' at KAM-4, showing everything distinct from the ferruginous quartzite/sandstone discussed above. As well as the (meta)quartzite just discussed, this includes small frequencies of other materials. These include rhyolite, chert, and quartz (Supplementary figure 22). Quartz occurs widely, although variably, as small round pebbles in the Palaeozoic sandstone of the area. Chert is rare at KAM-4. Where present it is generally of low quality. Some may be exported from relatively far away, but our study of the area has revealed the presence of 'lacustrine chert' in some of the lakebeds in the sand sea. A very clear example was found at the WNEF16_4 site mentioned above. This lacustrine chert has not yet been described in detail. The only reported site in which it occurs relatively frequently is Al Wusta, on the southern side of the western Nefud[20]. Al Wusta is one of the few sites in the area where there is limited ferruginous quartzite available locally, so it seems that hominins used older lakebeds as a chert source. Informal knapping experiments by HSG suggest that at least for the material found in the Al Wusta basin, it is a challenging material to knap. It has to be struck with considerable force, and has a thick cortex. However, if the inner part of nodules can be accessed, then it can be relatively good quality. Some of the chert lithics, particularly from the Southeast Lake, are clearly forms of this lacustrine chert. Finally, rhyolite lithics (and occasional other examples of igneous material) are from an unknown source. They may come from primary sources in the form of rhyolite dykes on the southern side of the Nefud, or they may also have been collected from fluvial deposits.

In summary, aside from occasional more 'exotic' raw materials (although perhaps still all, or at least mostly, available relatively locally), most of all of the KAM-4 assemblages are made on a similar moderate quality ferruginous quartzite. We can now consider the technological characteristics of the different assemblages. Our aim here is to give an overview of the major features of each of the assemblages.

Assemblage A (Central Lake, MIS 11) is a small assemblage of 42 lithics (Extended figure 4). Although a few older flakes were recovered from Ti's al Ghadah[61], the much larger size of Assemblage A means that it is the oldest meaningful lithic assemblage currently known from Arabia. The key aspect of the assemblage is the ten handaxes present. These have consistent morphological characteristics, as summarised in supplementary tables 10 and 11. They were produced by façonnage shaping of rectangular blocks of ferruginous quartzite. They are small, with an average weight of 207 grams and average length of 114 ±19 mm. They are consistent in their shape characteristics, being elongate (mean length to width ratio 1.73 ± 0.22) and highly refined (mean width to thickness ratio 0.37 ± 0.05), although we recognise the small sample size for this assemblage. Finally, no evidence of core and flake technology

was present in Assemblage A, either as a source of handaxe blanks, or as a separate reduction strategy.

Assemblage B (Northeast Lake, MIS 9) is much larger, and represents the final phase of Acheulean at the site. The sample size of 383 lithics allows a clear characterization of the assemblage. Flakes and other debitage are well represented, and include many clear biface-thinning flakes. The key feature of the assemblage is the presence of 78 handaxes (Extended Figures 5 and 6). This larger sample size allows a detailed description of this assemblage. Three are of quartzite, while the rest of ferruginous quartzite/sandstone. There is once again exclusively *façonnage* reduction of rectangular blocks to produce bifaces, although in Assemblage B there is also a distinct core and flake technology to which we will return below. The Assemblage B handaxes are often quite highly weathered, making aspects such as the precise reading of flake scars problematic. But their shape outlines are clear. Assemblage B handaxes are sometimes plano-convex (i.e. one surface is relatively flat). They have a consistently pointed shape, with minimal working at the proximal end. These are very small handaxes, with a mean length of 92 ± 25 mm (Supplementary table 12). There is no difference in the proportion of the margin worked between assemblages A and B (Mann-Whitney U=358, p=0.637), so the significantly smaller length values for B (Mann-Whitney U = 182, p = 0.006) are unlikely to be explained by increased reduction intensity. Like the assemblage A handaxes, those of assemblage B are also highly refined (mean thickness to breadth ratio 0.4 ± 0.1). This thinness and small size (with a quarter weighing less than 69 grams and being less than 76 mm in length) leaves the possibility that some of these handaxes were not even hand-held tools as they could potentially have been hafted. In the smallest cases these Assemblage B bifaces are not much larger than typical MSA points, yet unlike the latter they are purely made by bifacial façonnage. They seem to represent a process of taking Acheulean biface reduction to its limits. Notably, within assemblage B there is no relationship between the proportion of the margin that has been flaked and the length of the handaxe (R=0.078, df=77, F=0.462, p=0.499), so the smaller ones do not seem be the result of differential reduction intensity.

In inter-regional terms, the Assemblage B bifaces stand out for their small size, high refinement, and mean elongation. Supplementary tables 14 to 17 for instance summarised sites with >25 published biface assemblages from Shipton and Petraglia[133-134]. These show that Assemblage B bifaces are some of the smallest, elongate, and in particular, refined handaxes from the Acheulean world. In terms of comparisons to the neighbouring Levant, Supplementary table 14 summarises mean biface length for Acheulean and Acheulo-Yabrudian sites following[134-146]. This reveals a decline through time, through the temporal facies, from (averages) 148.0 mm in the Early Acheulean, 122.3 mm in the Middle Acheulean, 90.1 mm in the Late Acheulean, and 80.8 mm in the Acheuo-Yabrudian. These values show that the 94.3 mm mean length for KAM-4 assemblage B is actually fairly typical for late Middle Pleistocene bifaces in Southwest Asia; however, their refinement is exceptional[147,p.41].

An interesting feature of Assemblage B is the presence of a core and flake component as well as the bifacial element (Supplementary Figure 28). As far as can be determined, all of the handaxes are made by the *façonnage* reduction of angular clasts, and therefore seems to represent a distinct reduction sequence to the production of small flakes from cores. Given the distribution of these cores, and of Levallois flakes, in Assemblage B in the area of the Northeast Lake that is proximal to the Northwest Lake (Supplementary figures 11 and 2), it is

important to consider whether they are genuinely part of Assemblage B rather than representing mixture with Assemblage B. Given the sparsity or absence of Levallois technology in the Acheulo-Yabrudian assemblages of the Levant (see SI 1), the presence of a Levallois component in Assemblage B offers an important part in the understanding of the assemblage. While several different types of core are found in Assemblage B, the most common type are preferential Levallois cores with centripetal preparation (Supplementary Figure 28).

That the core reduction component of Assemblage B is a genuine part of the assemblage is indicated by several lines of evidence. Firstly, they are considerably more weathered than comparable material from the Northwest Lake. However, as discussed above, weathering of lithics at the site is complex and variable within assemblages. The spatial distribution of cores and flakes may also be relatively proximal to the Northwest Lake, but the distribution of Assemblage B along the Northeast Lake does not closely resemble what we might imagine if a certain part of Assemblage B had somehow been transported from the Northwest Lake. The distribution of cores (supplementary figure 12) and Levallois flakes (supplementary figure 11) in Assemblage B mirrors the distribution of handaxes (supplementary figure 9), of which there is not a trace in Assemblage C, in that the area of greatest density is midway along the Northeast Lake ridge. There are also indications from the shape and size characteristics of cores in Assemblages B and C that they can be distinguished. For instance, Assemblage B cores are typically much larger, with mean and median weights of 138.6 g and 89.2 g, compared to 60.0 and 43.9 g for Assemblage C. This size difference can be expressed in several ways: for example, the mean and median maximum thickness for cores is 29.7 mm and 28.7 mm for Assemblage B and 21.3 and 20.0 mm for Assemblage C. This core and flake element is a relatively small part of Assemblage B, which is dominated by handaxes and associated flakes. Many of the flakes have the clear morphology of biface thinning flakes (Supplementary figure 29). Only three retouched flakes were recovered.

Assemblage C (Northwest Lake, MIS 7) marks a significant technological shift compared to the earlier Assemblages A and B. Not only are no handaxes present, but not even any flakes consistent with bifacial reduction. The assemblage is classically Middle Palaeolithic in its characteristics. Core reduction was primarily Levallois (Extended figure 8, Supplementary Figure 30), mostly of a preferential character, but with a small recurrent centripetal element (supplementary table 19). An interesting feature of Assemblage C is that Levallois flakes are diverse in their scar patterns (supplementary table 24). While centripetal Levallois cores are the most common form of core, only 42.9% of the Levallois flakes have centripetal scar patterns. For instance, bidirectional scar patterns are relatively common at 28.6%. Retouched flakes are not common in the assemblage. Those present are broken side retouched flakes, while one Levallois flake shows either retouch or damage on its margins. Assemblage C represents the first relatively large MIS 7 Middle Palaeolithic assemblage to be described in Arabia (the only other example, JQ-1 is described below).. The rather lesser focus on centripetal reduction compared to MIS 5 assemblages shows it has a somewhat different technological character to this better understood more recent phase.

Assemblage D (Southeast Lake, MIS 5?) is the largest assemblage recovered from KAM-4 (supplementary table 9). With 44 cores, 464 flakes, and 25 retouched flakes it offers a useful comparative sample. On chronological grounds the assemblage dates to either the terminal Middle Pleistocene, or, most likely, the early Late Pleistocene. Most of the raw material for assemblage D consists of ferruginous quartzite, but there is somewhat more diversity than with the earlier assemblages. For instance, of the cores, 17 (39%) consist of materials other

than the dominant ferruginous quartzite/sandstone. These different materials consist of different quartzites, fine grained igneous rocks, rhyolite, and cherts. Likewise, for the general debitage, the 'exotic' raw materials are fairly common at 19.9%. This pattern might be interpreted as indicating fairly common import of non-local cores to the site, which were then often abandoned and ferruginous quartzite, of presumably local origin, flaked instead. As an aside, we point out that non-local raw material offers a useful way to evaluate the integrity of the site. For instance, with Assemblage D a dense scatter in the eastern part of the Southeast lake had multiple rhyolite lithics (e.g. supplementary figure 13). The dense and localised distribution of these, as well as identification of refits (supplementary figure 25), gives an example of surface scatters preserving valuable spatial information, showing that the site has good integrity.

Assemblage D is similar to MIS 5 assemblages known from elsewhere in the region. Beginning with the core technology, Assemblage D is dominated by Levallois cores (Supplementary table 19). More than 2/3 of the cores consist of different forms of centripetal Levallois cores, particularly preferential with centripetal preparation. That said, there is some diversity to the core forms in Assemblage D, such as the presence of five pieces interpreted as cores on flakes. However, as shown with the examples of supplementary figure 31, the main focus of cores is on the production of Levallois flakes by the centripetal preparation of cores. Likewise, 74% of the Levallois flakes have centripetal scar patterns (Supplementary figure 32, Supplementary table 24). An interesting component of Assemblage D is the relatively large (by current Arabian standards) sample of retouched flakes. About half (52%) of these are broken. They all consist of relatively simply retouched flakes, mostly with side retouch and several also having end retouch. The average length of the complete pieces is 59 mm and an average weight of 42.9 g. The retouch is all on the dorsal surface and is virtually all semi-abrupt and continuous (that is, the kind of retouch often described as 'scraper-retouch'). Seemingly 'exotic' raw material is fairly common (32%) in the retouched tools.

Assemblage E (South Lake, MIS 3?), the final assemblage at the site, is found at the far south of KAM-4. It is a fairly small assemblage, but with enough pieces to characterise its basic features (supplementary table 9). Only a maximum (underlying) age is available for Assemblage E, suggesting that it either dates to the late Middle Pleistocene or Late Pleistocene. While by no means definitive, we hypothesise that Assemblage E dates to early MIS 3. This is primarily because of the character of lithics, as described below, but the sedimentary characteristics of the South Lake, i.e. a thin deposit indicating a relatively minor increase in humidity, are consistent with the early MIS 3 wet phase. The Assemblage E cores are diverse (Supplementary table 10; Supplementary figure 33). Levallois forms are the most common, and centripetal forms the most common of those. This, then, is a Middle Palaeolithic assemblage but one of a somewhat ambiguous character compared to the other KAM-4 assemblages, in terms of cores. A clearer signal may come from the Levallois flakes. While a relatively small sample (n=15 complete), the most common dorsal scar pattern on these is unidirectional convergent (47%). Furthermore, there is a tendency for convergent lateral margins in these pieces, which in some cases is achieved by a single supplementary removal in a different direction to the predominant pattern of removals (see examples in supplementary figure 34). In other words, the Levallois flakes for Assemblage E show a focus on producing pointed flakes by unidirectional-convergent preparation, but this is sometimes supplemented by small numbers of other scars creating scar patterns which are technically bidirectional or centripetal. These flakes are quantitatively compared to other Levallois flake samples in SI 10. When it comes to the general flake population (i.e. non-Levallois flakes), unidirectional scar patterns are the most common.

The incongruence between Levallois flakes, which record the aims of knapping during most of the reduction process (and general flakes), and residual core forms has often been noted in the Levantine late Middle Palaeolithic assemblages. So while Assemblage E, which is a small assemblage, currently has a somewhat unclear chronology and technical character, our working model is that it dates to the same time as sites such as ALM-3[27] and SD-1[23], that is early MIS 3, ca. 59-55 ka.

8. JQ-1 and JSM-1 lithic assemblages

8.1 JQ-1

Previous work at Jebel Qattar-1 reported the discovery of a small (n = 28) assemblage of lithics from MIS 7 deposits[28]. The artefacts were found at a consistent stratigraphic position, associated with the OSL estimate of 211 ± 16 ka. Most of the lithics consisted of small and rather generic flakes, often broken, while two flakes were of a Levallois character and indicated a Middle Palaeolithic character for the assemblage.

As with the previous excavations, lithics were found in the new trenches at a very low density and in the form of single artefacts, with no indications of knapping scatters. The lithics all occurred in the same stratigraphic position as previously identified, in the MIS 7 deposit. Most of the lithics recovered are in a very sharp and fresh condition, the partial exception being corrosive weathering on the ferruginous quartzite pieces. Other materials (chert, rhyolite) are in very fresh condition.

A total of 45 new lithics were recovered from trenches A and B. Aside from a single fragment of a broken core, these consist of flakes, chips, and chunks. Most of the lithics are small (mean length = 24 mm, mean weight = 8 g). Combining the new findings with the previously recovered material gives a total assemblage of 73 lithics (examples shown in supplementary figure 35). Nothing indicating a Lower Palaeolithic technology (such as handaxe thinning flakes) was identified. At least five of the flakes can be classified as Levallois flakes (see for example furthest right in second row on supplementary figure 35). In some cases, broken flakes appear to also be Levallois flakes. Other flakes are consistent with being core preparation and shaping flakes, consistent with a Middle Palaeolithic Levallois technology. So while a small assemblage, our new excavations confirm the MIS 7 material from JQ-1 is of a Middle Palaeolithic character, and, coming from a well stratified context, forms a useful partner for the larger MIS 7 assemblage from KAM-4.

8.2 JSM-1

Previous work at JSM-1 described a small lithic assemblage[28] and the distinctive raw material at the site[66]. The small assemblage previously recovered indicated a focus on preferential centripetal Levallois technology. Additional features were hinted at, such as the presence of a small *façonnage* component, but renewed work found no evidence for this, and only one possible biface has been recovered from the surface of the site[28], which may in fact be a minimally flaked core.

Combining the 2013 season material with the sample reported by Petraglia and colleages[28] gives a total assemblage of 715 artefacts (Supplementary table 21). 91.3% of these are from excavations, with the rest collected from the immediate associated surface as erosion of artefact bearing deposits has occurred (Supplementary table 22). The site gives excellent insights into the human occupation of northern Arabia in late MIS 5, representing the largest excavated late MIS 5 assemblage in northern Arabia. Further comparative studies will be

reported in the future, for now we describe the basic technological characteristics of the assemblage. Virtually the entire assemblage consists of the kind of 'argillaceous sandstone' previously described by Groucutt and colleages[66]. Other materials (chert, quartz, ferruginous quartzite) occur in small amounts (for instance, the cores consist of 62 argillaceous sandstone pieces and three quartz. Likewise, for the general debitage, 93% of lithics are of this argillaceous sandstone).

A total of 65 cores have been recovered from JSM-1 (supplementary table 23). These present a consistent picture, in which aside from occasional examples of particular core types (such as two multidirectional cores), reduction was focussed on a consistent approach, emphasising centripetal Levallois reduction. Twenty percent of the cores can be described as tested cores and preforms. These are informative on the kinds of clasts selected for reduction, and highlight the thinness of these pieces, with cortex on both outer surfaces. After centripetal preparatory removals, these cores were primarily reduced in a preferential manner, but with the new sample also revealing a significant recurrent centripetal component. The Levallois cores are of a consistent character, with faceting platforms, cortical bases, and centripetal preparation (see[28] for examples of cores). In most regards the recurrent centripetal and preferential centripetal Levallois cores are similar (e.g. average number of scars >5 mm in length is 15 and 13 respectively, average length is 46 and 54 mm). The recurrent cores are typically somewhat smaller (average 47 g, compared to 77 g) and seemingly somewhat more reduced, which may indicate a tendency towards recurrent exploitation as reduction proceeded, but on the whole the indications are of an interchangeable strategy where the two methods were used depending on the clast morphology and nature of previous removals.

The significantly enlarged Levallois flake population (n=46) offers additional insights into the character of reduction at JSM-1. As shown in extended figure 9, these Levallois flakes show complementary characteristics to the cores. They almost all have facetted striking platforms and are parallel-sided to oval shape in character, with convergent and laminar shapes rare. They are predominantly (83.3%) characterised by centripetal scar patterns. The mean average dimensions of complete JSM-1 Levallois flakes are 37.3 mm technical length, 32.1mm maximum width, 8.9 mm maximum thickness, and 14.0 g weight. They are therefore typically small in size and with regular morpho-technological features.

The general debitage population is dominated by small pieces (average weight = 4.3 g). Of the general debitage, 378 are classed as chips and chunks, and a further 218 as flakes. These flakes have an average weight of 8 g and length of 26 mm. They mostly have plain platforms, while facetted and cortical forms are also common. These flakes are consistent with being preparation flakes for the kind of centripetal Levallois technology indicated by the Levallois cores and flakes described above. Cortex is common, with an average cortical cover of 30%. Supplementary figure 36 shows an example of a group of flakes recovered close together in layer 3C of trench 3, these are typical JSM-1 flake forms.

The sample of retouched flakes from JSM-1 is small, but informative. The eight pieces consist of three retouched points, three side retouched flakes, one end retouched flake and one possible biface (which could also be seen as a core). The small sample of retouched forms precludes detailed comparative studies. In contrast to the general assemblage characteristics, over half of the retouched pieces are on raw materials other than the argillaceous sandstone which dominates the assemblage (one quartz, one ferruginous quartzite, three fine grained igneous). This may indicate these pieces were left at the site as it

was visited for ‘retooling’, the site having basically the character of a workshop, beside a raw material source.

We return to comparative aspects later (using PCA), but for now we emphasise some basic points. Firstly, in terms of what is lacking. Evidence for unidirectional-convergent reduction of the form commonly found with Levantine Neanderthals is lacking, as is the Nubian Levallois technology sometimes emphasised in recent dispersal models[148], and a *façonnage* component, as seen in the early MIS 5 Jebel Faya assemblage[22] is likewise absent at JSM-1 aside from a single possible piece (found on the surface). These features are likewise absent from the Middle Palaeolithic assemblages at KAM-4. In positive terms, the JSM-1 assemblage shares basically technological characteristics with other MIS 5 assemblages both from Arabia, the Levant, and northeast Africa. Examples of mid to late MIS 5 sites with a similar focus on centripetal Levallois technology include JQ-1[28], MDF-61 at Mundafan[58], Al Wusta[20], Qafzeh[14], Panga ya Saidi[149], and Aduma[150]. JSM-1 therefore continues a very clear regional technological trend, and adds a valuable comparative sample to allow quantitative comparisons. JSM-1 at ca. 75 ka also fills a pivotal spatial and temporal context, occurring at the very end of MIS 5, and therefore being younger than sites such as Qafzeh and Al Wusta.

9. Inter-regional lithic comparison

Following on from the general typo-technological descriptions given above, we conducted a comparative analysis of the Middle Palaeolithic assemblages we have reported with material from the surrounding regions. We performed a quantitative comparison of Levallois flakes. We have chosen Levallois flakes for comparison as they allow a highly quantitative comparison, being more common than retouched forms and arguably less ambiguous than residual core forms. Levallois technology is a prominent part of Middle Palaeolithic/Middle Stone Age assemblages in the study area, and Levallois flake morphology can be described using basic and replicable attributes. We used measurements (all in mm where appropriate) – the number of flakes scars over 5 mm in length, flaking length, proximal width, width at midpoint, distal width, thickness at the midpoint, striking platform width and striking platform thickness – which are easy to measure. These measurements also have the benefit that they can be extracted from published illustrations, as we did for the Misliya assemblage. Together, the eight variables selected allow the quantitative description of Levallois flake shape. These different shapes were the result of different methods of core preparation and exploitation.

We used principal components analysis (PCA) to search for structure in two lithic datasets (one for late Middle Pleistocene sites and one for early to middle Late Pleistocene sites). The raw data and R script are available at https://github.com/wccarleton/multipledispersals and are archived with Zenodo (DOI:10.5281/zenodo.5082969). PCA is used to identify major axes of variability in a multidimensional dataset and to reduce the number of dimensions (measured variables) from a large set of correlated ones to a smaller set of new, uncorrelated ones (principal components, or PCs) [151]. The aim of the PCAs was to explore how assemblages C, D and E from KAM-4 and the JSM-1 assemblage were similar/different in morphology to assemblages from elsewhere in Arabia and surrounding regions. The lithic data was directly recorded by the authors in all cases except Misliya Cave, where the detailed publication of the site allowed data to be extracted from the numerous illustrated Levallois flakes (which seem to be representative of the total collection, according to their features such as scar patterns).

As a preliminary step, we determined whether each dataset contained sufficient variation along one or more potential axes to justify running PCAs. This was done with Bartlett's Test for Sphericity[152] and the Kaiser, Meyer, Olkin (KMO) Measure of Sampling Adequacy[153]. Both are standard, simple statistical tests designed to determine whether a multidimensional point cloud is non-spherical, which would indicate that one or more major axes of variation could be uniquely identified. We used relevant functions from the R "psych" package to perform the tests[154]. The results of both tests indicated that the data were sufficiently non-spherical for one or more major axes to be extracted. For the KMO Measure, the standard benchmark is an overall Measure of Sample Adequacy (MSA) greater than 0.5 and the benchmark for Bartlett's Test for Sphericity is a p-value of < 0.05. The overall MSA was 0.73 for the Late Middle Pleistocene dataset and 0.78 for the Late Pleistocene dataset; and the p-value from the sphericity test was far less than 0.05 for both datasets (R reported values of

< $2.2x10^{-16}$). Note that the Late Palaeolithic (LP) dataset contains n = 404 observations and the Late Middle Pleistocene dataset contains n = 92 observations.

Next, we used the built-in R function, "prcomp" [155], to extract the PCs and determine their relative importance in terms of the variation they account for in each dataset. The datasets were first scaled and centred prior to analysis. Two standard decision rules were then used to determine how many PCs we would retain for further analysis. One criterion was that retained PCs should have eigenvalues greater than or equal to 1, and the other was that retained PCs should account for the majority of variation in the data. For the PCA involving the Late Pleistocene assemblages, these criteria meant we focused primarily on the first two PCs. With respect to the PCA involving the late Middle Pleistocene assemblages, the criteria meant we focused on the first three PCs. The PC loadings tables for each dataset are available in supplementary tables 25 and 26. For the Middle Pleistocene sample, PC 1 is driven by a spread of variables, particularly those emphasising width. Given the negative loading of all variables, this PC is prominently being driven by size. PC 2 mostly strongly emphasised length (positive) and distal width (negative), that is, it emphasises a shape pattern contrasting long and convergent flakes from shorter and more parallel sides flakes. For the Late Pleistocene comparison, the first component was again largely driven by size, with all variables loading positively, while PC 2 was most strongly driven by the number of scars and distal width, and finally PC 3 was mostly strongly driven by flaking length (negative) and platform thickness (positive). PC 2 distinguishes convergent flakes with few scars from more parallel sided flakes with more scars. PC 3 distinguishes short flakes with thick platforms from longer flakes with thin platforms.

We then transformed the original measurements into the new PC variables for each observation in both datasets. Once a PC has been extracted, the relevant observations can be reprojected onto the PC - often this process is called "rotation" because it entails rotating the variable space such that the major axes align with the PCs. Thus, instead of a single lithic observation having a length measurement, a width measurement, and so on, it is associated instead with a PC1 measurement, a PC2 measurement, etc. These rotated variables were then used to look for distinctions among assemblages in each of the two datasets with respect to the new PC variables. We used box plots to represent the distributions of a given PC variable for a given assemblage as a simple visual way of displaying the data. Note that the points in these plots (supplementary figures 38 and 39) have been randomly shifted on the horizontal axis to increase their visibility. Their y-axis positions have not been changed, so the vertical spread of the points remains correctly represented.

For the late Middle Pleistocene PCA, the three assemblages largely overlapped for PC1, indicating a range of Levallois flake sizes in these assemblages. However, PC2 presents an interesting pattern in which the assemblages are rather distinct in terms of shape. The AHS assemblage (Omo Kibish, Ethiopia) has the lowest PC2 score, and Misliya the highest, with KAM-4 in between (supplementary figure 38). Given the loadings, the PCA is here distinguishing elongated and pointed Levallois points compared to shorter and more parallel sided flakes. This quantitative distinction matches technological descriptions of the assemblages, where AHS emphasises centripetal Levallois technology[30] and Misliya Cave a focus on unidirectional Levallois point production[31]. For instance, centripetal scar patterns on Levallois flakes occur rarely at Misliya, 43% of the time at KAM-4 assemblage C, and 69% of the time at AHS. Both the Levallois flake shape data (PC2) and general technological considerations of the assemblages suggest that KAM-4 assemblage C, the earliest Middle Palaeolithic assemblage with a good sample size, is more similar to African assemblages

such as AHS than Levantine Early Middle Palaeolithic assemblages are. This may either suggest a developmental sequence reflecting dispersal into Arabia and subsequent development of the distinctive unidirectional and convergent focus of the Levantine early MP, or may alternatively been seen as reflecting the presence of highly structured populations at this time.

For the Late Pleistocene PCA, a larger sample of comparative assemblages is available (supplementary figure 39). PC1, primarily reflecting size, does not reveal any clear differences in time and space. PC 2 reveals an interesting pattern. The assemblages with high scores are those associated with *Homo sapiens* fossils (BNS, Al Wusta; although Qafzeh XIX falls rather centrally, perhaps again indicating the development of specific technologies in the Levantine woodland zone). Low scores for PC2 are seen with the Neanderthal associated assemblages of Kebara and Tor Faraj. This division matches technological descriptions of early to middle Late Pleistocene *Homo sapiens* technologies in the region, but adds a systematic quantitative perspective. The positive values for JSM-1, in particular, and KAM 4 assemblage D orientate these assemblages to MIS 5 *Homo sapiens* assemblages in the sample. The analysis also provide support for other Arabian MIS 5 assemblages belonging to *Homo sapiens*.

The mostly negative (75% below 0 [Supplementary Figure 39]) scores for KAM-4 assemblage E orientate this assemblage to Neanderthal associated assemblages from the Levant. This matches the technological aspects that we have discussed above. For instance, while 74% and 83% of Levallois flakes from KAM-4 assemblage D and JSM-1 have centripetal scar patterns, just 35.7% from KAM-4 assemblage E do. PC3 value are relatively close together for the different assemblages and do not indicate a clear pattern.

While further, more detailed, lithic comparative studies will be conducted in the future. Our PCA of Levallois flakes offers instructive comparative details and offers hypotheses to be tested. For the late Middle Pleistocene it highlights differences in Levallois flakes between East Africa, Arabia, and the Levant. Furthermore, these differences occur when large and relatively simple handaxes were still being made in central Arabia[24]. This patterning suggests at a minimum highly structured hominin populations, and perhaps even different species of hominin in the region. For the Late Pleistocene our analysis supports previously qualitative distinctions between *Homo sapiens* and Neanderthal assemblages in the area, with KAM-4 assemblage D and JSM-1 orientating with the former and KAM-4 assemblage E with the latter.

10. Fauna and biogeography

Much of what we know about the Pleistocene biogeographical history of Southwest Asia comes from the woodland regions of the central Levant, where long running research programs and cave-rich karstic substrates have produced a wealth of well-preserved fossil material[4]. Although some faunal exchange between Africa and the Levant did occur during the Middle and Late Pleistocene, Palaearctic fauna continuously dominated the Levantine fossil record indicating a relatively stable woodland environment during that period[4]. Here, we provide the first dates for fossil fauna recovered from KAM-4 in the western Nefud Desert – a vastly different environment to the Levantine woodlands to the north – greatly adding to our understanding of the past ecology and biogeography of this poorly understood region. Supplementary figure 14 shows the distribution of fossils at KAM-4.

McClure[156] was the first to report on Pleistocene fossils from the Arabian Peninsula when he described remains of hippopotamus, buffalo, equid, among other animals, from the Empty Quarter in the peninsula's southeast. More than a decade later, Thomas and colleagues[157] reported three fossil-bearing palaeolake deposits from the western Nefud Desert, northern Saudi Arabia, which included remains of straight-tusked elephant (cf. *Palaeoloxodon* sp.), hippo, hyena, and fish. Taken together, these fossil assemblages, along with the palaeolake deposits with which they were associated and stable isotope data, provided the first clear evidence of past savanna-like environments in regions of Arabia that are today characterized by hyper-arid deserts.

Since then, a number of important fossil deposits have been discovered in the western Nefud Desert, particularly in the last five years ([e.g. 20,26,33,56,62]). Renewed investigations at the Middle Pleistocene site of Ti's al Ghadah (*c*. 500–300 ka) recovered a wealth of fossil material that, in addition to the fauna previously reported by Thomas and colleagues, included a variety of small rodents, carnivores, reptiles, and water birds[60], as well as the earliest dated evidence for hominins in Arabia in the form of lithic artefacts and butchered bone[61-62]. Newly identified taxa bolstered earlier palaeoenvironmental reconstructions and additional stable isotope analyses of fossil herbivore teeth demonstrated that the Ti's al Ghadah environment was likely characterized by productive grasslands with aridity levels comparable to modern-day East African savannas[61]. The Al Wusta *Homo sapiens* fossil finger bone, dated to *c*. 85 ka and representing the oldest directly dated evidence for humans in Arabia, was found alongside remains of taxa including hippo, cf. *Syncerus*, and ostrich[20]. A Last Interglacial presence is also indicated by recently discovered human footprints at the site of Alathar (*c*. 121–112 ka) where footprints and fossils of elephants, camels, equids, and bovids were also discovered[56].

Most recently, Stewart and colleagues[33] conducted a widespread taxonomic and taphonomic study of nine fossil assemblages from the western Nefud Desert including those from the KAM-1 and KAM-4 palaeolakes. Likewise, the fossil fauna, which included hippo, elephant, *Syncerus* sp., and *Hippotragus* sp., are indicative of well-watered open-grassland environments. However, many of these sites lacked the chronological data needed to elucidate

the palaeoenvironmental and palaeobiogeographical history of the Arabian Peninsula, and the late Middle Pleistocene in particular has remained very poorly understood. The new OSL and U-series dating results presented here provides a chronological framework with which to begin to address these issues.

Perhaps the most notable taxon in the Arabian Pleistocene fossil record is hippo. These are semi-aquatic animals that inhabit rivers, lakes, and mangrove swamps in which they live, socialize, and breed[158]. Their physiological dependence on water and sensitivity to arid conditions makes them excellent indicator species for relative humidity and the presence of large, permanent water bodies. Their presence in the Arabian fossil record is therefore of great interest and remains of hippos have now been recovered from at least four sites in the western Nefud Desert[20,33], as well as further south in the Empty Quarter[156]. Significantly, as hippos would have been unable to persist in the Arabian interior during arid glacial phases, the fossil record implies that they repeatedly dispersed during the pluvial phases of MIS 5 (as at Al Wusta, Site 16.3, KAM-1, and Empty Quarter), MIS 7 (as at KAM 4 NW lake), and as early as MIS 9 (as at KAM NE Lake). The most likely dispersal route appears to have been along palaeohydrological corridors that connected the Nefud Desert to the Levant[10] where hippos persisted well into the Holocene[159.]

Proboscideans are also relatively common in the Arabian fossil deposits but, with the exception of those from Ti's al Ghadah[56,62], are typically represented by very few remains. Unfortunately, the KAM-4 NW lake fossils are too few and fragmentary to provide detailed taxonomic insight[33]. And given the recent discovery of cf. *Palaeoxodon recki* and cf. *Elephas hysudricus* at Shishan Marsh in Jordan, dating to the late Middle Pleistocene[160], the identity of the KAM-4 remains cannot be assumed. Likewise, it remains difficult to speculate as to the maker of the proboscidean footprints found at Alathar, although the environment and size of the footprints is suggestive of a grassland adapted species larger than any of the extant elephants (e.g., *P. recki*) [56]. Interestingly, elephants are seemingly absent from the Levant from around 400 ka onwards[161], and their presence in the western Nefud Desert may have made the region particularly attractive to dispersing hominins. However, until the fossil record is better resolved, it remains unclear whether elephant populations persisted in refugia in southwest Asia during arid phases (e.g., Shishan Marsh), dispersing outwards into regions such as the Nefud Desert during periods of climatic upturn, or undertook numerous dispersals into Arabia from Africa and/or Eurasia.

The large wild cattle *Syncerus* ("*Pelorovis*") has been identified at a number of Late Pleistocene sites in the Nefud Desert including KAM-1, Site 16.3, and Al Wusta[20,33] and the age of these remains makes attribution to *S. antiquus* almost certain. A once widespread species, by the Late Pleistocene *S. antiquus* was mostly restricted to the Maghreb and Egypt, suggesting a biogeographical connection between northern Africa and the Arabian interior at that time[4]. Likewise, remains of African golden wolf (*Canis anthus*) from Ti's al Ghadah[62] and *Hippotragus* sp. from WNEF16_15[33] provide additional links to Africa. Other taxa indicate possible dispersals from Eurasia, including the Asiatic wild ass (cf. *Equus hemionus*) at two late Pleistocene sites in southern Arabia[156], as well as at Ti's al Ghadah[60] at which remains of the Eurasian jaguar (*Panthera* sp. cf. *P. gombaszoegensis*) were also recovered. In addition, a number of endemic taxa have also been recovered including arid-adapted oryx (*Oryx* sp.) and gazelle (*Gazella* sp.) that continue to inhabit the peninsula today[33,60].

Taken together, the fossil, isotopic, and geomorphological data clearly demonstrate that during pluvial periods of the Middle and Late Pleistocene parts of the Arabian Peninsula were

characterized by expansive grasslands and large, perennial water sources. The fossil record comprises a growing composite of African, Eurasian, and endemic taxa including various mammals, reptiles, and birds. Immigrating fauna, and particularly large water-dependent mammals such as hippos, likely utilized the palaeohydrological corridors that connected the Arabian interior to the Levant and Northeast Africa[10]. Hominins, too, would have been able to exploit these corridors including the animals they encountered along them. The emerging fossil record from Arabia, with its diverse influences and features such as repeated connections to African fauna [also indicated by rock art depictions[162]], has a very different character to the faunal record from the woodland Levant, which is dominated by the long-term dominance of Palearctic fauna such as *Dama mesopotamica*.

11. Summary and implications of results

Our findings from KAM-4 and Jubbah – as described in the main text and preceding supplementary sections – have a number of implications for understanding changes in hominin demography and behaviour in both Southwest Asia and adjacent regions, and how these relate to climatic and environmental changes.

For the younger time periods, as represented by Northwest Lake at KAM-4 (ca. 200 ka), JQ-1 (ca. 210 ka), and JSM-1 (ca. 75 ka), chronological resolution is high enough to allow relatively precise attribution to particular temporal/climatic phases. Further attributions remain more hypothetical, such as our suggestion that Southeast Lake at KAM-4 dates to around ca. 55 ka. To our new findings we can of course add previously reported sites from Arabia, such as those dating to around 120 ka[20], those dating to ca. 90-80 ka[20,58], and those dating to ca. 55 ka[23,27.] Our findings are consistent with, and further extend, the temporal patterning of hominin occupations in Arabia. For KAM-4, each phase of hominin occupation is associated with a phase of lake formation, providing evidence for regional climatic amelioration, with increased rainfall. While evaporation would still have been high, this was offset by lakes being groundwater fed and steep sided interdunal basins providing relatively small lake surface areas relative to depth. While these lakes were small, the character of the sediments and fauna associated with them demonstrates that they were perennial and predominantly freshwater (see also[22]).

One key question is to ascertain the regional and global climatic conditions in which the phases of environmental amelioration and associated hominin occupations in Arabia occurred. For the older phases at KAM-4 – particularly Central Lake at ca. 400 ka and, to a lesser extent, Northeast Lake at ca. 300 ka – the large uncertainties associated with the luminescence age estimates allow less precise chronological attributions than for younger periods. However, situating our findings in the context of knowledge on regional Quaternary climatic and environmental dynamics allows us to suggest the most likely ages. Aridity was the dominant characteristic of late Middle Pleistocene and Late Pleistocene Arabia[5,17,21]. While some complexity in how climatic changes leads to resulting environmental change comes from factors such as underlying geology and orographic impacts, the past climate of Arabia in broad terms is increasingly well understood. Climate models suggest that the periodic amelioration of Arabia primarily relates to the intrusion of the African monsoon system[163]. Given that Arabia sits at the interface of different weather systems[164], it is possible that other sources were also involved at times. However, as evidenced by records such as high-resolution southern Arabian speleothems[21], the periods of major environmental amelioration reflect the intrusion of monsoonal precipitation. Since monsoon strength is primarily controlled by boreal summer insolation, the most pronounced periods of regional humidity probably correspond to precessional peaks during global warm periods such as MIS 5, 7, 9 and 11. This has led to a perceived association between humidity and interglacials as traditionally primarily defined in northern hemisphere terms[165]. However, there is also evidence for significantly enhanced precipitation in Arabia outside of interglacials, as indicated by multiple sites dating to MIS 5a,

such as Al Wusta and Mundafan al Buhayrah (MIS 5e represents the MIS 5 interglacial). Furthermore, emerging evidence from MIS 3 suggests that there was a phase of increased precipitation ca. 55 ka[23,27], but this left more ephemeral evidence than humid periods during phases such as MIS 5. It may, for instance, have been defined by a period of extended storms with relatively arid conditions between them[23,166]. Similarly, MIS 6 saw periods humid enough to lead to extensive alluvial fan formation in eastern Arabia[167]. The climatic history of Arabia is therefore relatively complex, with varying levels of aridity and humidity. During 'glacial' periods such as MIS 6 and MIS 3 there were phases of somewhat increased precipitation, yet the major wet periods which led to the formation of significant lakes probably relate primarily to precessional peaks within interglacial periods, as well some substages such as MIS 5a.

Consequently, while it is true that at the temporal resolution available to most archaeological studies, pronounced enhancement of Arabian precipitation occurs during interglacials, the monsoon system is predominantly driven by obliquity-modulated precession. At maximum (positive) precession, higher insolation during the boreal summer leads to an increased north-south and cross-equatorial pressure gradient (XEPG), with a subsequent intensification of low-latitude rainfall systems. As such, while major wet phases in Arabia occur in line with northern hemisphere interglacials such as MIS 5e, less pronounced (although evidently critical) periods of increased rainfall occur during stronger glacial processional peaks.

This regional climatic picture, as summarised in figure 2 in the main paper, brings context to our findings from KAM-4 and Jubbah. From ca. 200 ka onwards it is clear that humid phases are associated with the more pronounced precessional peaks. Although the uncertainty on the luminescence age for Central Lake at KAM-4 is large, long periods of aridity either side of ca. 400 ka, suggest a probable attribution to MIS 11 (Fig. 2). Both Arabian (e.g. speleothem) and wider-regional signals of monsoonal activity, such as the East Mediterranean sapropel record, indicate there was dominant aridity for long periods either side of MIS 11 (Fig. 2). We therefore suggest that taking this regional situation into account, allows a probable period of lake formation and hominin occupation at KAM-4 to be proposed. The dominant pattern at KAM-4 appears to be of lake formation during either interglacial periods or in association with pronounced precessional peaks. The suggestion that Southeast Lake may date to MIS 3, and therefore corresponds to a more muted precessional peak, fits well with the sedimentary characteristics of this small deposit which consists of a thin layer of marl. The Southwest Lake may date to a late MIS 6 substage, but given the uncertainties on the luminescence dates and the fact that the sample was taken underneath the main phase of lake formation shown by a marl deposit, it is not impossible that it dates to MIS 5e.

In archaeological terms, our results highlight high levels of material culture variability within Southwest Asia and that no single area can be used as a proxy for the entire region. This complexity seems to reflect multiple pulses of population dispersal into, and within, the region. This was then followed by variable demographic and behavioural trajectories; from the establishment of relatively long cultural phases (i.e. spanning multiple marine isotope stages) in the central Levantine woodland area, such as the Acheulo-Yabrudian, through to seemingly ephemeral occupations in northern Arabia. Sites such as KAM-4 and the Jubbah localities indicate hominin occupations correlating with occasional humid episodes during periods of enhanced monsoons, seemingly followed by northern Arabian depopulation. Southern and eastern Arabia seems to have followed a pattern somewhere between that of the Levantine woodlands and northern Arabia, with evidence that populations survived long enough to develop distinctive kinds of material culture, but perhaps not across multiple isotope stages (SI 1).

One key aspect of this picture is that northern Arabia seems to present closer demographic connections to Africa than either southern Arabia or the Levantine woodlands do. This pattern is evident in both the material culture and fossil record of the region. Faunal assemblages in the Levantine woodland zone repeatedly show a Palearctic character[4], and material culture phases such as the Acheulo-Yabrudian and Levantine early Middle Palaeolithic with its focus on point production are both absent from further south. Little is known of the Pleistocene fauna of southern and eastern Arabia, but the repeatedly distinctive, localised, character of material culture suggests that crossing the Red Sea at the Bab al Mandeb was not a primarily dispersal route and that instead populations filtered through northern Arabia. In northern Arabia the growing fossil record suggests repeated connections to Africa across a contiguous grassland zone through the southern Levant which formed during repeated humid episodes (discussed in SI 10). To that we can add significant aspects of material culture which we have reported in this paper. The absence of Acheulo-Yabrudian assemblages in northern Arabia, and the southern Levant, suggests that the Late Acheulean in this area relates more to Africa than to areas to the north. Likewise, with the early Middle Palaeolithic at KAM-4 (Assemblage C of the Northwest Lake) both technological features (such as the methods of Levallois surface preparation, see SI 7) and quantitative characteristics in terms of PCA of Levallois flake shape situate the assemblage between the Levantine Early Middle Palaeolithic the early Middle Stone Age in East Africa (SI 9).

With MIS 5, similar assemblages are found across a large area of Northeast Africa and Southwest Asia. One point we emphasise here is that the classic Levantine assemblages from Qafzeh and Skhul date to around 120-90 ka. Our work at JSM-1 shows an assemblage with classic regional MIS 5 characteristics of the area at a rather younger date, of around 75 ka (SI 8 and 9). While there remains much to clarify here, the emerging picture may suggest multiple pulses of dispersal out of Africa within MIS 5, including a terminal MIS 5 one currently only known from Arabia. Our previous work has suggested MIS 5 assemblages in northern Arabia are more similar to East African MSA assemblages than the Levantine assemblages are[168]. However, finally, our findings demonstrate that the Palaeolithic of Arabia cannot simply be seen in terms of a dispersal route from Africa, as KAM-4 Assemblage E – as well as sites elsewhere in Arabia dating to ca. 55 ka and demonstrating a focus on unidirectional flaking of convergent flakes (SI 7 and 9) – is consistent with the southward spread of Neanderthals.

With the caveat that the diverse regional behavioural and demographic trajectories exist, our findings highlight repeated hominin population dispersals from Africa into Southwest Asia, during periods of increased regional precipitation. This has implications for a number of debates in human evolutionary studies. The greater similarities between the material culture of northern Arabian/southern Levant Late Acheulean sites with African Late Acheulean sites than with Levantine woodland zone Acheulo-Yabrudian sites is consistent with pulses of dispersal from Africa in this period. Further research is needed to elucidate the character and extent of these dispersals. When it comes to the final stages of the Middle Pleistocene, our findings contribute to debates on the transition to the Middle Palaeolithic and evidence for very early dispersals of *Homo sapiens* out of Africa. While fossil evidence from the eastern Mediterranean has been argued to demonstrate early *Homo sapiens* dispersals into the area[e.g.48], this evidence is patchy and coarsely dated[49]. It has been argued that genomic evidence indicates dispersals of African populations into Eurasia in this time, the late Middle Pleistocene. For instance, Posth and colleagues[169] suggest a dispersal out of Africa in the 460-219 ka time range. In archaeological terms, Akhilesh and colleagues[170] argued that technological changes at the Indian site of Attirampakkam in the ca. 385 to 172 ka timeframe suggested an early transition

to the Middle Palaeolithic. Together then, recent years have seen a growth of arguments and evidence for significant late Middle Pleistocene behavioural changes and population dispersals. The arguments from palaeontology, genetics, and archaeology for early dispersals provide further information for discussions on interactions between populations and significant changes such as the transition to the Middle Palaeolithic. Our findings add an important component to these debates, with northern Arabian archaeological assemblages being consistent with repeated pulses of hominin dispersal into Southwest Asia.

In MIS 5 there is more secure evidence for the presence of *Homo* sapiens in the Levant and Arabia (SI 1), and perhaps as far east as Southeast Asia[171,172.] These dispersals have been widely discussed in archaeological terms[3,173-177] A key point of the findings we report here, as well as a general increase in knowledge on MIS 5 Arabia, is the occurrence of very late MIS 5 (ca. 75 ka) occupations at JSM-1. Earlier studies had hinted at a late MIS 5 *Homo sapiens* presence in Arabia (SI 1), but with the large excavated assemblage from JSM-1 this is put on more secure footings. This supplements earlier work[196] emphasising multiple waves of dispersal within MIS 5, with sites such as Skhul being up to 50 thousand years older than JSM-1. While multidisciplinary analyses and debates on the fates of MIS 5 dispersals will continue, and evidence remains sparse in the low latitudes, a key point here is that the MIS 5 'early dispersal' model does not mean a single dispersal phase, focussed on MIS 5e, but rather multiple pulses of dispersal associated with at least three humid phases in MIS 5. The presence of spatial variability in MIS 5 material culture – such as contrasting Jebel Faya Assemblage C with assemblages elsewhere in Arabia – matches the expectation of high levels of population structure at this time[178,179]. The possible MIS 3 presence of Neanderthals in Arabia may suggest that they expanded further south than previously thought, and highlights that there is currently little clarity on where the main pulse of admixture between *Homo sapiens* and Neanderthals occurred, beyond probably Southwest Asia broadly.

As a closing note, we emphasise that as well as our results being consistent with repeated pulses of hominin dispersal out of Africa into Southwest Asia, the possibility of movement in the reverse direction should be kept in mind. Given factors such as current uncertainty on the background to the earliest known *Homo sapiens* in Africa[e.g.178], and discussions on the possible involvement of a hominin closely related to *Homo antecessor* as an ancestor of our species[180], currently only known from Eurasia, as a precursor to *Homo sapiens*, building reliable records for the later Quaternary in Southwest Asia is not only important for understanding 'out of Africa' dispersals, but also for 'into Africa' dispersals.

Supplementary acknowledgements

Lithic data for the comparative analysis of Levallois flakes was (SI 9) was collected from a variety of institutions. We thank the following researchers, curators, and institutions for access to their collections: the National Museum of Ethiopia and particularly Sahle Melaku, and Alison Brooks, John Yellen, John Shea, and John Fleagle for agreeing for us to study lithics from their excavations. The Anthropology Department and particularly Donald Henry at the UT, Oklahoma, the Peabody Museum at Harvard and particularly Christian Tryon, the Institute of Archaeology at HUJI and particularly Erella Hovers, and the British Museum (HG).

Supplementary tables

Site(s)	Lat. N	Long. E	Chronometric age estimates	Hominin fossils?	Fauna?	Reference(s)
Wadi Surdud complex (particular SD-1)	15.190	43.428	50-60 ka		x	23
Mundafan (MDF-61)	18.550	45.300	80-90 ka			58
Umm al-Sha'al	24.335	47.160	65-90 ka			59
206-76, Saffaqah	24.344	44.516	180-260 ka			24
Jebel Faya	25.119	55.847	ca. 40-120 ka, 110-140 ka			22, 181
Al Wusta	27.418	39.397	85-95 ka	x	x	20
Ti's al Ghadah	27.433	39.373	300-500 ka?		x	60,61
Alathar	27.520	39.398	110-120 ka		x	56
Al Marrat-3 (ALM-3)	27.733	40.485	50-60 ka			27
Khall Amayshan-1 (KAM-1)	27.961	39.352	100-120 ka		x	25
Jubbah sites (JQ-1, JKF-1, JSM-1)	28.000	40.932	50-100 ka, 210 ka			This paper and 28,66
Khall Amayshan-4 (KAM-4)	28.031	39.353	410 ka, 310 ka, 200 ka, <150 ka		x	This paper and 33
An Nasim	28.273	39.693	Ca. 300 ka			29
Tor Faraj/Sabiha	29.948	35.460	50-70 ka		x	32,182
Rosh Ein Mor	30.850	34.750	50-70 ka (or 200 ka?)		x	183,184
'Ain Difla	30.880	35.530	100-180 ka		x	185
Far'ah II	31.280	34.490	45-70 ka		x	186
Oumm Qatafa	31.700	35.300	190-440 ka	x	x	187
Revadim	31.772	34.816	300-500 ka		x	188,189
Nesher Ramla	31.900	34.920	70-180 ka		x	190
Azraq sites (e.g. SM-1)	32.001	36.560	70-130 ka, > ca. 250-300 ka		x	191
Qesem Cave	32.102	34.987	200-400 ka	x	x	192
Holon	32.110	34.802	180-220 ka		x	193
Emanuel Cave	32.160	35.170	<190 ka		x	194
Kebara (MP)	32.560	34.930	50-70 ka	x	x	15
Skhul	32.670	35.080	100-130 ka	x	x	195,196
Tabun	32.670	34.970	Multiple, ca. 100-500 ka	x	x	197, 198
Qafzeh	32.680	35.300	90-100 ka	x	x	14
Ein Qashish	32.684	35.108	55-70 ka	x	x	51
Misliya	32.741	34.992	150-240 ka	x	x	31, 48, 199
Zuttiyeh	32.856	35.527	90-180 ka?	x	x	200
Amud	32.869	35.499	50-70 ka	x	x	201, 202
Shovakh	32.900	35.499	50-70 ka	x	x	203
Hayonim	32.930	35.220	140-250 ka		x	204,205
Nahal Mahanayeem Outlet (NMO)	33.024	35.628	65-55 ka		x	206, 207

Manot	33.035	35.193	> ca. 55 ka	x		[208]
Quneitra	33.120	35.804	50-60 ka		x	[209]
Naamé	33.730	35.460	80-100 ka		x	[210, 211]
Ksar Akil (MP layers)	33.920	35.620	> 50 ka		x	[212, 213]
Ras el Kelb	33.930	35.600	90-120 ka?	x	x	[214]
Yabrud	33.970	36.620	100-240		x	[215]
Nahr Ibrahim	34.080	35.650	60-110 ka		x	[215, 216]
Douara	34.680	38.399	40-90 ka		x	[217]
Umm El Tlel	35.090	38.610	Multiple layers, MP ca. 40 to at least 80 ka		x	[218, 219]
Hummal	35.320	38.520	Multiple, ca. 50 ka -500 ka	x	x	[220, 221]
Nadaouiyeh Aïn Askfar			Multiple, ca. 50 ka -500 ka			[222]
Dederiyeh	36.399	36.867	50-90 ka	x	x	[223]
Shanidar	36.800	44.200	50-70 ka (older at base?)	x	x	[224]

Supplementary Table 1. List of dated archaeological and hominin palaeontological sites in Southwest Asia between 50 and 500 ka, see supplementary figures 1 and 2 for mapping (sites very close together shown as a single point). The sites are listed from south to north. Selected references are given, with an emphasis on recent papers and those focussing on chronology. Coordinates are approximate in some cases as precise latitudes and longitudes have not been published in all cases. Sites with only a few lithics are not included, nor where ages are very problematic. The site of Alathar has no stone tools or hominin fossils, but has footprints assigned to *Homo* cf. *sapiens*. Where there are a few discreet assemblages at a site, ages are separated by commas, for age ranges (reflecting both chronological uncertainty and repeated occupations) a dash is used to give summary age range.

Assemblage	Phase	MIS	Raw mat.	LCT	Lev.	Microlithic	Main reduction method(s)	Faunal remains	Ref.
KAM 4-A	LP	11	FQ	x			Bifacial		This paper
17.2	LP	11/9	FQ	x			Bifacial		This paper; [18]
An Nasim	LP	9	FQ	x			Bifacial		[29]
KAM 4-B	LP	9	FQ	x	x		Bifacial, Levallois component	cf. *Hippopotamus*	This paper; [33]
JQ-1 lower	MP	7	C, R, FQ		x		Centripetal Levallois?		This paper; [28]
KAM 4-C	MP	7	FQ (X)		x		Levallois, centripetal is most common, but diverse	*Ostrich,* Elephant, *Nesokia, Hippopotamus*, Horse	This paper; [33]
KAM 4-D	MP	5?	FQ (C,R)		x		Centripetal Levallois	*Oryx*	This paper; [33]
KAM-1	MP	Early 5	FQ		x		Centripetal Levallois	*Hippopotamus, Pelorovis,*	[25]
JSM-1	MP	5e/5a	A.S.		x		Centripetal Levallois		This paper [28]
Al Wusta	MP	Late 5	C, Q, FR		x		Centripetal Levallois, multiplat.	*Hippopotamus, Pelorovis, Kobus, Ostrich*	[20]
JQ-1 upper	MP	Late 5	FQ (R,C)		x		Centripetal Levallois, discoidal		[28,68]
JKF-1	MP	5/3	FQ, Q (R,C)		x		Uni. Convergent Levallois		[28]
KAM-4-E	MP	3?			x		Diverse Levallois, unidirectional and centripetal common		This paper; [33]
ALM-3	MP	3	FQ		x		Uni. Convergent Levallois		[27]
Al Rabyah	EP	1	C,Q			x	Single platform/microblade.		[225]

Supplementary Table 2. Summary of information on Palaeolithic assemblages of northern Arabia. This only includes assemblages which are associated with dated sediment formations (i.e. excavated or clearly associated with a particular deposit). LP = Lower Palaeolithic, MP = Middle Palaeolithic, UP = Upper Palaeolithic, EP = Epipalaeolithic. Raw material is the dominant one, if significant numbers of others are shown they are in brackets. FQ = ferruginous quartzite, C = chert, R = rhyolite, Q = quartz, And = Andesite.

Lake	Deposit length	Deposit width	Deposit area (m²)	Max. altitude	Min. altitude	Altitude range	Mean altitude
**Central**	154	48	5,959.0	919.3	909.8	9.4	916.5
**Northeast**	517	83	29,990.3	922.3	911.4	11.0	916.5
**Northwest**	175	85	12,015.2	920.6	913.4	7.2	918.4
**Southwest**	154	8	3,227.2	920.9	919.4	3.5	919.0
**South**	24	8	141.5	919.7	919.1	0.63	919.5
**Southeast**	239	130	23,729.7	920.4	914.6	5.8	917.9

Supplementary Table 3. Data on the different sedimentary deposits at KAM-4.

Step	Quartz single-grain	Quartz multigrain aliquot	Feldspar $pIRIR_{290}$	Feldspar $pIRIR_{225}$
1	Give regenerative dose[1]	Give regenerative dose[1]	Give regenerative dose[1]	Give regenerative dose[1]
2	Preheat 1 (260 ^{0}C for 10 s)	Preheat 1 (240 ^{0}C for 10 s)	Preheat 1 (320 ^{0}C for 60 s)	Preheat 1 (250 ^{0}C for 60 s)
3	IR diode stimulation (50 ^{0}C for 100 s)	--	IR diode stimulation (50 ^{0}C for 200 s)	IR diode stimulation (50 ^{0}C for 200 s)
4	Green laser stimulation (125 °C for 2 s)	Blue diode stimulation (125 ^{0}C for 200 s)	IR diode stimulation (290 ^{0}C for 200 s)	IR diode stimulation (225 ^{0}C for 200 s)
5	Give test dose	Give test dose	Give test dose	Give test dose
6	Preheat 2 (220 ^{0}C for 10 s)	Preheat 2 (160 °C for 10 s)	Preheat 2 (320 ^{0}C for 60 s)	Preheat 2 (250 ^{0}C for 60 s)
7	IR diode stimulation (50 ^{0}C for 100 s)	--	IR diode stimulation (50 ^{0}C for 200 s)	IR diode stimulation (50 ^{0}C for 200 s)
8	Green laser stimulation (125 °C for 2 s)	Blue diode stimulation (125 ^{0}C for 200 s)	IR diode stimulation (290 ^{0}C for 200 s)	IR diode stimulation (225 ^{0}C for 200 s)
9	--	--	IR diode stimulation (325 ^{0}C for 100 s)	IR diode stimulation (325 ^{0}C for 100 s)
10	Return to step 1	Return to step 1	Return to step 1	Return to step 1

[1] In the first cycle, where the natural luminescence intensity is observed, no regenerative dose was administered.

Supplementary Table 4. The Single-Aliquot Regenerative-dose (SAR) protocols used in this study

Sample[a]	Measured (n)	Rejection criterion[b]							Accepted	
		1) Signal intensity	2) Recycling	3) Recuperation	4) IR depletion	5) D_e uncertainty	6) Saturation	7) Zero D_e (JSM only)	n	%
PD7 (Q-DR)	48	0	3	0	17	0	0	N/A	28	58.3
PD12 (Q-DR)	24	0	1	0	9	0	0	N/A	14	58.3
PD7 (Q-DR)	48	0	2	0	21	1	0	N/A	24	50.0
PD8 (Q)	48	0	1	0	19	1	0	N/A	27	56.3
PD9 (Q)	48	0	1	0	19	2	0	N/A	26	54.2
PD10 (Q)	11	0	1	0	0	0	0	N/A	10	90.9
PD11b (Q)	48	0	2	0	17	2	0	N/A	27	56.3
PD12 (Q)	48	0	1	0	12	1	0	N/A	24	50.0
PD13 (Q)	48	0	4	0	16	2	0	N/A	25	52.1
PD14 (Q)	10	0	0	0	0	0	0	N/A	10	100.0
PD14 (Q)	24	0	4	0	0	0	0	N/A	20	83.3
PD14 (KF)	17	0	0	0	0	0	0	N/A	17	100.0
KAM4-OSL1 (KF)	15	2	0	0	N/A	0	4	N/A	9	60.0
KAM4-OSL2 (KF)	14	1	0	0	N/A	0	0	N/A	13	92.9
KAM4-OSL3 (KF)	10	0	0	0	N/A	0	0	N/A	10	100.0
KAM4-OSL4 (KF)	10	0	0	0	N/A	0	0	N/A	10	100.0
KAM4-OSL5 (KF)	10	1	1	0	N/A	0	0	N/A	8	80.0
KAM4-OSL6 (KF)	10	0	1	0	N/A	0	0	N/A	9	90.0
JSM-OSL7 (Q)	6400	6032	149	85	53	0	4	4	73	1.1
JSM-OSL9 (Q)	4400	4141	83	74	21	0	8	2	71	1.6
JSM-OSL12 (Q)	4800	4570	75	81	11	0	5	1	57	1.2

Supplementary Table 5. Number of single aliquots and grains rejected / accepted after application of the rejection criteria and used in the calculation of D_b

[a] Measurements made on quartz (Q) or K-feldspar (KF), DR indicates that the data are for a dose recovery test;

[b] Rejection criteria. Aliquots/grains were rejected where: 1) Signal intensity = The natural signal from the aliquot/grain could not be distinguished from the background signal (determined using Luminescence Analyst "sig. >3 sigma above BG" rejection criterion); 2) Recycling = The recycling ratio differed from unity by >20 %; 3) Recuperation = The sensitivity-corrected zero dose luminescence intensity was >5 % of the natural luminescence intensity; 4) IR depletion = the IR-depletion ratio was more than two standard errors below unity (quartz only); 5) D_e uncertainty = The uncertainty on D_e exceeded 30 %; 6) Saturation = The natural luminescence signal (L_n/T_n) intercepts the dose response curve at a point where growth has ceased. In addition, grains from samples coded "JSM" were rejected where D_e

was consistent with zero at two standard errors (7) Zero D_e). This additional criterion followed established practice at the site[68] and was added to exclude modern grains incorporated during sampling.

Sample	Context[1]	Mineral[2]	Grain size (μm)	Depth (m)	Dose rates (Gy/ka)				
					α[3]	β[3]	γ[3]	Cosmic	Total[4]
KAM4-OSL1	KAM-4 Central Lake (US)	KF	180 - 255	0.75	0.05 ± 0.01	0.31 ± 0.02	0.25 ± 0.01	0.21 ± 0.02	1.63 ± 0.15
KAM4-OSL2	KAM-4 Northeast Lake (US)	KF	180 - 255	0.75	0.05 ± 0.01	0.39 ± 0.02	0.25 ± 0.01	0.21 ± 0.02	1.71 ± 0.15
KAM4-OSL3	KAM-4 Northeast Lake (OS)	KF	180 - 255	0.47	0.03 ± 0.01	0.23 ± 0.01	0.26 ± 0.01	0.23 ± 0.02	1.56 ± 0.15
KAM4-OSL4	KAM-4 Southeast Lake (US)	KF	180 - 255	0.45	0.06 ± 0.01	0.47 ± 0.02	0.27 ± 0.01	0.24 ± 0.02	1.84 ± 0.15
PD11b	KAM-4 Southeast Lake (US)	Q	180 - 210	0.40	--	0.30 ± 0.02	0.24 ± 0.01	0.24 ± 0.02	0.78 ± 0.04
PD12	KAM-4 Southeast Lake (US)	Q	180 - 210	0.80	--	0.33 ± 0.03	0.29 ± 0.02	0.21 ± 0.02	0.84 ± 0.04
PD9	KAM-4 Southwest Lake (US)	Q	180 - 210	0.75	--	0.20 ± 0.02	0.22 ± 0.01	0.21 ± 0.02	0.63 ± 0.03
PD10	KAM-4 Southwest Lake (IL)	Q	4 - 11	0.40	0.08 ± 0.01	0.63 ± 0.05	0.26 ± 0.01	0.24 ± 0.02	1.21 ± 0.06
PD7	KAM-4 South Lake (US)	Q	180 - 210	0.45	--	0.19 ± 0.02	0.20 ± 0.01	0.24 ± 0.02	0.63 ± 0.03
PD8	KAM-4 South Lake (US)	Q	180 - 210	0.40	--	0.20 ± 0.02	0.21 ± 0.01	0.24 ± 0.02	0.65 ± 0.03
KAM4-OSL5	KAM-4 Northwest Lake (IL)	KF	180 - 255	0.32	0.05 ± 0.01	0.46 ± 0.02	0.34 ± 0.02	0.20 ± 0.02	1.86 ± 0.15
KAM4-OSL6	KAM-4 Northwest Lake (US)	KF.	180 - 255	1.02	0.1 ± 0.03	1.08 ± 0.05	0.42 ± 0.02	0.25 ± 0.03	2.65 ± 0.16
PD13	KAM-4 Northwest Lake (US)	Q	180 - 210	0.80	--	0.33 ± 0.03	0.27 ± 0.01	0.21 ± 0.02	0.81 ± 0.04
PD14	KAM-4 Northwest Lake (IL)	KF	60 - 90	0.32	0.11 ± 0.01	0.65 ± 0.05	0.43 ± 0.02	0.25 ± 0.03	1.39 ± 0.06
PD14	KAM-4 Northwest Lake (IL)	Q	60 - 90	0.32	0.10 ± 0.02	0.62 ± 0.05	0.43 ± 0.02	0.25 ± 0.03	1.40 ± 0.06
PD14	KAM-4 Northwest Lake (IL)	Q	4 - 11	0.32	0.10 ± 0.02	0.62 ± 0.05	0.43 ± 0.02	0.25 ± 0.03	1.43 ± 0.06
JSM OSL7	JSM-1 Layer 2 (IL)	Q	180 - 210	0.86	0.10 ± 0.02	0.33 ± 0.01	0.33 ± 0.03	0.20 ± 0.02	0.76 ± 0.04
JSM OSL9	JSM-1 Layer 3 (IL)	Q	180 - 210	1.14	0.10 ± 0.02	0.36 ± 0.01	0.38 ± 0.04	0.20 ± 0.02	0.83 ± 0.05
JSM OSL12	JSM-1 Layer 4 (IL)	Q	180 - 210	1.59	0.10 ± 0.02	0.37 ± 0.01	0.35 ± 0.04	0.19 ± 0.02	0.79 ± 0.04

Supplementary Table 6. Dose rates and associated data for the KAM4, PD and JSM sites.

[1]US = Samples taken from sediments underlying the marl, which therefore provide a maximum age for the lake, OS = samples taken from sediments overlying the marl, which therefore provide a minimum age for the lake and IL = samples taken from within the marl, which therefore date the lake directly.

[2]Q = Quartz, KF = K-feldspar.

[3]Dose rates have been corrected for an assumed moisture content of 5.0 ± 2.5 % where appropriate.

[4]Total dose rates for K-feldspar samples include an internal dose rate of 0.81 ± 0.15 Gy/ka.

Sample	Context[1]	Mineral[2] (signal)	Grain size (μm)	Model[3]	OD[d4]	Total Dose rate (Gy/ka)	D_b (Gy)[5]	Age (ka)[6]
KAM4-OSL1	KAM-4 Central Lake (US)	KF ($pIRIR_{290}$)	180 - 255	CAM	55	1.63 ± 0.15	673 ± 127	412 ± 87
KAM4-OSL2	KAM-4 Northeast Lake (US)	KF ($pIRIR_{290}$)	180 - 255	CAM	26	1.7 ± 0.15	578 ± 44	337 ± 39
KAM4-OSL3	KAM-4 Northeast Lake (OS)	KF ($pIRIR_{290}$)	180 - 255	CAM	37	1.56 ± 0.15	479 ± 58	306 ± 47
KAM4-OSL4	KAM-4 Southeast Lake (US)	KF ($pIRIR_{290}$)	180 - 255	CAM	11	1.84 ± 0.15	435 ± 18	237 ± 22
PD11b	KAM-4 Southeast Lake (US)	Q (multigrain)	180 - 210	CAM	17	0.78 ± 0.04	121 ± 6	154 ± 11
PD12	KAM-4 Southeast Lake (US)	Q (multigrain)	180 - 210	CAM	11	0.84 ± 0.04	126 ± 5	149 ± 9
PD9	KAM-4 Southwest Lake (US)	Q (multigrain)	180 - 210	CAM	22	0.63 ± 0.03	118 ± 7	184 ± 14
PD10	KAM-4 Southwest Lake (IL)	Q (multigrain)	4 - 11	CAM	8	1.21 ± 0.06	174 ± 10	143 ± 10
PD7	KAM-4 Southeast Lake (US)	Q (multigrain)	180 - 210	CAM	17	0.63 ± 0.03	106 ± 6	168 ± 12
PD8	KAM-4 Southeast Lake (US)	Q (multigrain)	180 - 210	CAM	33	0.65 ± 0.03	93 ± 7	142 ± 13
KAM4-OSL5	KAM-4 Northwest Lake (IL)	KF ($pIRIR_{290}$)	180 - 255	CAM	47	1.86 ± 0.15	353 ± 61	190 ± 36
KAM4-OSL6	KAM-4 Northwest Lake (US)	KF ($pIRIR_{290}$)	180 - 255	CAM	74	2.65 ± 0.16	213 ± 53	80.4 ± 20.6
PD13	KAM-4 Northwest Lake (US)	Q (multigrain)	180 - 210	CAM	35	0.81 ± 0.04	116 ± 9	141 ± 13
PD14	KAM-4 Northwest Lake (IL)	Q (multigrain)	60 - 90	CAM	13	1.39 ± 0.06	272 ± 14	194 ± 13
PD14	KAM-4 Northwest Lake (IL)	Q (multigrain)	4 - 11	CAM	13	1.43 ± 0.06	303 ± 19	210 ± 16
PD14	KAM-4 Northwest Lake (IL)	KF ($pIRIR_{225}$)	60 - 90	CAM	2	1.76 ± 0.09	318 ± 13	192 ± 20
JSM OSL7	JSM-1 Layer 2 (IL)	Q (single-grain)	180 - 210	FMM	26	0.76 ± 0.04	58.9 ± 4.0 (67%)	77.6 ± 6.7
							103 ± 9 (33%)	136 ± 14
JSM OSL9	JSM-1 Layer 3 (IL)	Q (single-grain)	180 - 210	FMM	68	0.83 ± 0.05	59.3 ± 4.2 (43%)	71.5 ± 6.4
							103 ± 6 (54%)	124 ± 10
JSM OSL12	JSM-1 Layer 4 (IL)	Q (single-grain)	180 - 210	FMM	35	0.79 ± 0.04	103 ± 5 (90%)	130 ± 10

Supplementary Table 7. Summary dating and age results.

[1] US = Samples taken from sediments underlying the marl, which therefore provide a maximum age for the lake, OS = samples taken from sediments overlying the marl, which therefore provide a minimum age for the lake and IL = samples taken from within the marl, which therefore date the lake directly.

[2] Q = Quartz, KF = K-feldspar.

[3] Model used to determine sample D_b values, CAM = Central Age Model, FMM = Finite Mixture Model.

[4] Overdispersion.

[5] The proportion of the data from each FMM component is in parentheses where appropriate.

[6] K-feldspar ages are corrected for fading.

Sample	U (ppm)	^{232}Th (ppb)	(^{230}Th/ ^{232}Th)	(^{230}Th/^{238}U)	(^{234}U/ ^{238}U)	Uncorr. ^{230}Th Age (ka)	Corr. ^{230}Th Age (ka)	Corr. Initial (^{234}U/ ^{238}U)
KAM16/85 (dentine)	18	112.2 ± 0.1	1094 ± 2	2.1940 ± 0.0036	1.4454 ± 0.0012	-	-	-
KAM16/85 (enamel)	4	2.71 ± 0.01	5664 ± 20	1.4169 ± 0.0031	1.5422 ± 0.0027	204.8 ± 1.5	204.8 ± 1.5	1.967 ± 0.004

Note: Ratios in parentheses are activity ratios calculated from the atomic ratios, but normalised to measured values of secular-equilibrium HU-1[226]. Errors are given at the 2σ level. ^{230}Th ages are calculated using Isoplot EX 3.0[227] with decay constants λ238 = 1.551x10-10 yr-1 (for ^{238}U), λ234 = 2.826x10-6 yr-1 (for ^{234}U) and λ230 = 9.158x10-6 yr-1 (for ^{230}Th), respectively[228]. 2σ errors in the uncorrected (uncorr.) ages were propagated directly from the uncertainties in the (^{230}Th/^{238}U) and (^{234}U/^{238}U). The corrected (corr.) ^{230}Th age was calculated using the assumed bulk earth or upper crust value equivalent to the detrital ^{230}Th/^{232}Th activity ratio of 0.83

Supplementary Table 8. Uranium series dating information for sample KAM16/85 (Northwest Lake).

Assemblage (lake)	Bifaces	Cores	flakes	retouched	Chips/chunks	Total
A (central)	10		30		2	42
B (Northeast)	78	24	245	3	33	383
C (Northwest)		16	151	6	26	199
D (Southeast)		44	464	25	197	730
E (South)		19	173	3	10	205

Supplementary Table 9. KAM-4 assemblage composition. Nb flakes includes Levallois flakes.

	N.	25%	75%	Mean	S.D.	C.V.
**Weight**	10	124.9	261.9	206.9	89.5	43.3
**Length**	10	103.7	128.2	114.2	19.3	16.9
**Thickness**	10	21.9	27.9	24.5	4.5	18.2
**Circumference**	10	254.0	315.1	285.2	46.1	16.2

Supplementary Table 10. Assemblage A handaxe size data

LAKE 1 (MIS 11)

	N.	25%	75%	Mean	S.D.	C.V.
**Edge shape**	10	0.52	0.62	0.59	0.10	17.0
**Elongation**	10	1.57	1.91	1.73	0.22	12.61
**Refinement**	10	2.39	2.91	2.74	0.44	16.2
**Weight/margins**	10	0.54	1.06	0.82	0.34	41.37

Supplementary Table 11. Assemblage A handaxe shape data

	N.	25%	75%	Mean	S.D.	C.V.
**Weight**	78	69.0	164.9	129.0	96.2	74.6
**Length**	78	75.8	109.3	94.3	25.4	26.9

**Thickness**	78	18.0	26.1	21.7	5.8	26.9
**Circumference**	78	194.0	275.3	236.9	72.6	30.6

Supplementary Table 12. Assemblage B handaxe size data.

	**N.**	**25%**	**75%**	**Mean**	**S.D.**	**C.V.**
**Edge shape**	78	0.49	0.63	0.57	0.11	19.69
**Elongation**	78	1.49	1.81	1.67	0.23	14.01
**Refinement**	78	2.19	3.00	2.67	0.77	28.75
**Weight/margins**	78	0.37	0.63	0.53	0.26	49.11

Supplementary Table 13. Assemblage B handaxe shape data.

**Region**	**Site**	**Mean Weight**
Africa	Kariandusi	552.7
	Olorgesailie DE89A	847.8
	Olorgesailie I3	253.0
Europe	High Lodge	259.9
SW Asia	Azraq Lion Spring	216.0
	**KAM-4, Ass. B**	**129.0**
South Asia	Hunsgi V	638.1
	Hungsi II	994.4
	Yediyapur VI	555.4
	Fatehgpur V	469.1
	Teggihalli II	350.8

Supplementary Table 14. Assemblage B mean handaze weight compared to comparative sample. Data from Shipton and Petraglia[133,134].

**Region**	**Site**	**Mean length**
Africa	Kariandusi	157.9
	Olorgesailie DE89A	180.8
	Olorgesailie I3	98.0
Europe	High Lodge	259.9
SW Asia	206-76	162.9
	**KAM-4, Ass. B**	**94.3**
South Asia	Hunsgi V	143.5
	Hungsi II	162.9
	Yediyapur VI	127.9
	Fatehgpur V	126.8
	Teggihalli II	121.24
	Anagwadi	137.24

Supplementary Table 15. Assemblage B mean handaxe length compared to comparative sample. Data from Shipton and Petraglia[133,134].

Region	Site	Mean length
Africa	Kariandusi	0.47
	Olorgesailie DE89A	0.45
	Olorgesailie I3	0.55
Europe	High Lodge	0.44
SW Asia	206-76	0.54
	Azraq Lion Spring	0.52
	**KAM-4, Ass. B**	**0.40**
South Asia	Hunsgi V	0.53
	Hungsi II	0.52
	Yediyapur VI	0.49
	Fatehgpur V	0.44
	Teggihalli II	0.47
	Anagwadi	0.58

Supplementary Table 16. Assemblage B mean handaxe refinement compared to comparative sample. Data from Shipton and Petraglia[133,134].

Region	Site	Mean length
Africa	Kariandusi	1.77
	Olorgesailie DE89A	1.75
	Olorgesailie I3	1.54
SW Asia	206-76	1.77
	**KAM-4, Ass B.**	**1.67**
South Asia	Hunsgi V	1.63
	Hungsi II	1.68
	Yediyapur VI	1.54
	Fatehgpur V	1.49
	Teggihalli II	1.52
	Anagwadi	1.70

Supplementary Table 17. Assemblage B mean handaxe elongation compared to comparative sample. Data from Shipton and Petraglia[133,134].

Phase	Assemblage	Mean length (mm)
Early Acheulean	Latamne	166.6
	Ubeidiya I-15a	143.8
	Ubeidiya I-15b	153.1
	Ubeidiya I-36	136.8
	Ubeidiya I-30a	138.7
	Ubeidiya I-30b	148.8
Middle Acheulean	GBY	142.1
	GBY B	144.1
	Evron Quarry	94.4
	Holon	100.8
	Umm Qatafa D2	117.5
	Umm Qatafa E	135.0
Late Acheulean	Azraq C-Pb	101.0
	Azraq C-Qa	88.0
	Azraq C-Qb	82.8
	Azraq T	107.7
	Azraq Lion Spring	102.9
	Berekhat Ram	67.5
	Beth Uziel	87.2
	Holon D	105.4
	Kissifum	105.3
	Maayan Barukh	114.0
	Rephaim-Baqa	102.6
	Revadim early	89.1
	Revadim late	73.3
	Ruhama	93.7
	Sahel el Koussin/Baram	89.7
	Tabun Ec	84.3
	Tabun Ed	85.0
	Tabun F	86.4
	Tabun Ronen EUA (350-370)	77.3
	Tabun Ronen EUA (310-340)	71.8
	Umm Qatafa D1	93.2
	Yiron	89.6
	Tabun XIV (unit 90)	74.0
Acheulo-Yabrudian	Bezez C	105.0
	Tabun Ea/Eb	87.6
	Misliya Cave terrace	84.6
	Tabun XI (bed 75)	85.1
	Tabun XI (bed 76)	72.3
	Tabun XII (bed 79)	72.1
	Tabun XII (bed 80)	69.4
	Tabun XIII (bed 83)	83.1
	Tabun X (bed 70)	84.5
	Tabun Ronen (210-240)	75.6
	Tabun Ronen (250-270)	69.3

Supplementary Table 18. Biface average length (mm) of bifaces in the Levant organised by traditional industrial phases. See text for sources.

	B	C	D	E
Tested	1 (4.2)		2 (4.5)	
Bidirectional			1 (2.3)	
Radial			2 (4.5)	1 (5.3)
Single platform	1 (4.2)	1 (6.3)		1 (5.3)
Multidirectional	1 (4.2)	1 (6.3)	1 (2.3)	
Discoidal	1 (4.2)			
Pref Lev with centrip. prep.	16 (67.0)	11 (68.8)	25 (56.8)	9 (47.4)
Rec. centrip. Levallois		2 (12.5)	6 (13.6)	3 (15.8)
Levallois indet.	2 (8.3)	1 (6.3)	2 (4.5)	2 (10.5)
Core on flake			5 (11.4)	1 (5.3)
Indet. (broken, weathered)	2 (8.3)			2 (10.5)
total	24	16	44	19

Supplementary Table 19. KAM-4 core typology by assemblage. Numbers (%).

	Bifaces	*Cores*	*flakes*	*retouched*	*Chips/chunks*	*Total*
JQ-1	0	1	61	0	11	73

Supplementary Table 20. JQ-1 MIS 7 assemblage composition

	Bifaces	*Cores*	*flakes*	*retouched*	*Chips/chunks*	*Total*
JSM-1	0	65	264	8	378	715

Supplementary Table 21. JSM-1 assemblage composition

**Context/Unit**	**Number**
1	201
2	232
3	159
4	23
Strat. Indet	62
Surface	38

Supplementary Table 22. JSM-1 summary of finds by context.

Core type	Number	%
Tested	4	6.2
Levallois preform/tested	9	13.8
Bidirectional	1	1.5
Radial	1	1.5
Single platform	3	4.6
Multidirectional	2	3.1
Pref Lev with centrip prep.	25	38.5
Rec. centrip. Levallois	13	20.0
Pref Lev with uni. Conv prep	1	1.5
Rec. unidirectional Levallois	1	1.5
Rec. bidirectional Levallois	1	1.5
Levallois indet.	4	6.2

Supplementary Table 23. JSM-1 core typology

	Uni	Uni Conv	Bidi	Centrip	Other	Total
KAM-4-C	1 (4.8)	2 (9.5)	6 (28.6)	9 (42.9)	3 (14.3)	21
KAM-4-D		3 (7.7)	5 (12.8)	29 (74.4)	2 (5.1)	39
KAM-4-E		7 (50)	1 (7.1)	5 (35.7)	1 (7.1)	14
JSM-1	1 (2.8)	2 (5.6)	3 (8.3)	30 (83.3)		36

Supplementary Table 24. scar patterns on Levallois flakes. Nb – only includes complete Levallois flakes (brackets in %).

Variable	PC1	PC2	PC3	PC4	PC5
**Number of Scars**	-0.1058	-0.3975	-0.8331	0.0385	-0.3238
**Flaking Length**	-0.2716	0.5960	-0.3679	0.0842	0.3267
**Width at Midpoint**	-0.4406	0.0372	-0.1162	0.1712	0.5092
**Proximal Width**	-0.4204	-0.0634	0.0858	0.5259	-0.2961
**Distal Width**	-0.2462	-0.6776	0.1677	-0.0009	0.4895
**Thickness at Midpoint**	-0.3901	0.1031	-0.0713	-0.5906	-0.0644
**Platform Width**	-0.4204	0.0924	0.3010	0.2630	-0.3831
**Platform Thickness**	-0.3932	-0.0550	0.1610	-0.5172	-0.2256

Supplementary Table 25. Loadings for late Middle Pleistocene PCA.

Variable	PC1	PC2	PC3	PC4	PC5
**Number of Scars**	0.1590	0.6196	-0.2903	0.6746	-0.2215
**Flaking Length**	0.3030	-0.2823	-0.7650	-0.0812	0.1986
**Width at Midpoint**	0.4275	0.0910	-0.1993	-0.2527	-0.0800
**Proximal Width**	0.4209	-0.2980	0.0743	0.0289	-0.3964
**Distal Width**	0.3027	0.4733	0.2014	-0.5330	-0.3343
**Thickness at Midpoint**	0.4062	0.1593	0.0494	-0.0954	0.4295
**Platform Width**	0.3526	-0.4330	0.2746	0.3538	-0.3326
**Platform Thickness**	0.3769	0.0484	0.4086	0.2350	0.5841

Supplementary Table 26. Loadings for Late Pleistocene PCA.

Supplementary figures

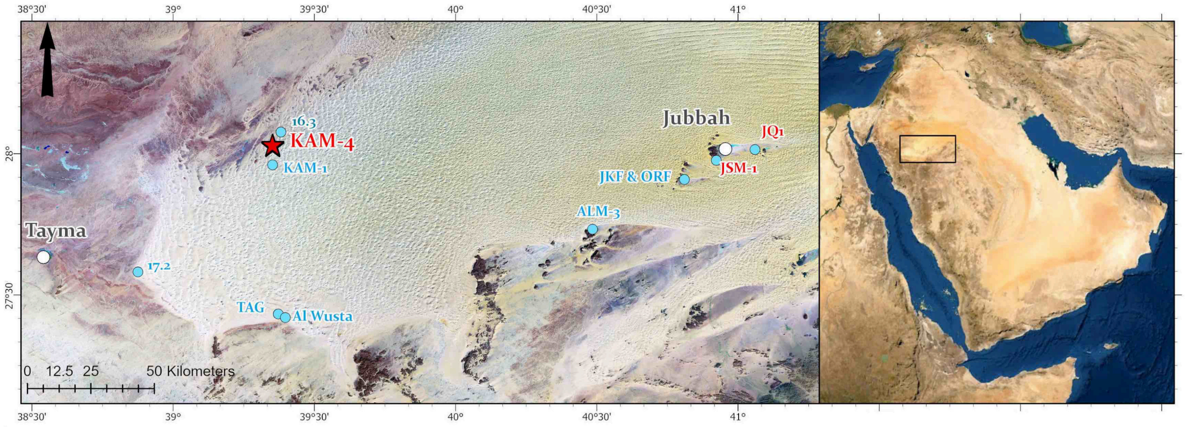

Supplementary figure 1. Location of KAM-4 and Jubbah sites (JSM-1 and JQ-1) in the Nefud Desert in northern Saudi Arabia. Other regional sites discussed in the text are also shown.

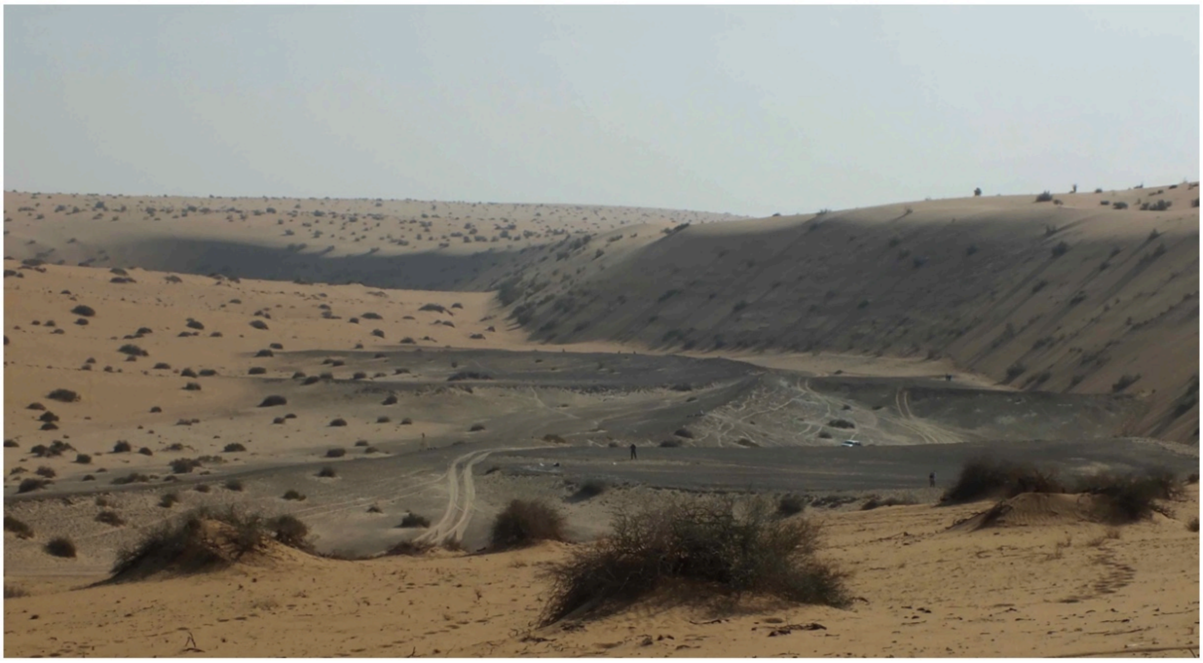

Supplementary figure 2. View of KAM-4 looking south. Two team members can be seen with a car between them, on the Northwest lake. Two more are in the back right, on the Central Lake mound.

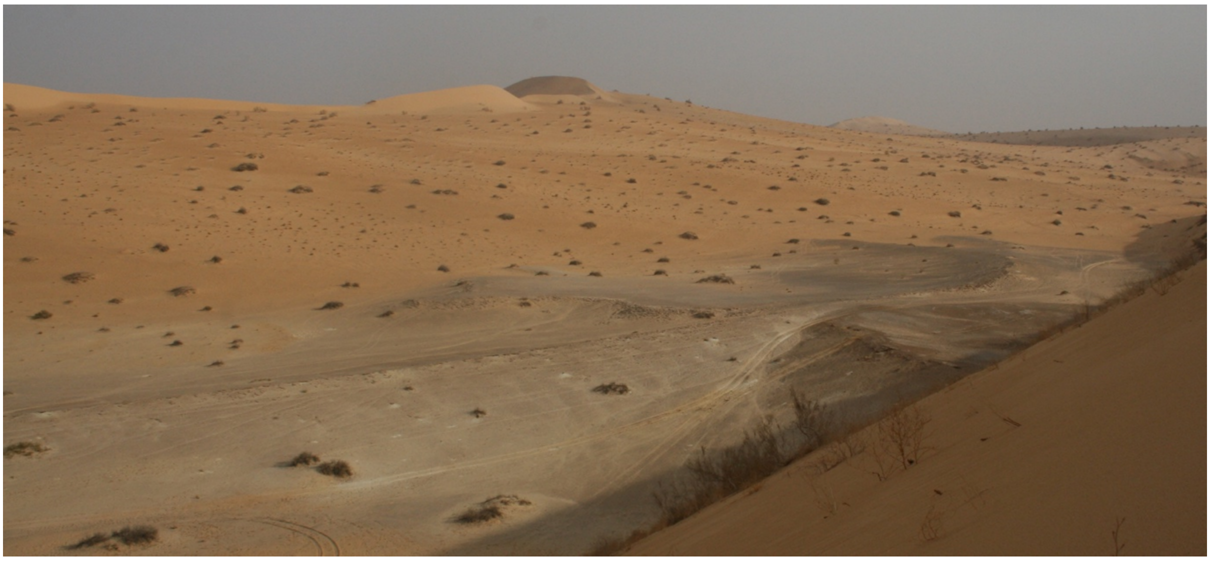

Supplementary figure 3. KAM-4, looking southeast from around the mid-point of the site. Northeast lake deposits can be seen on the left, Central Lake on the right and Southeast Lake in background.

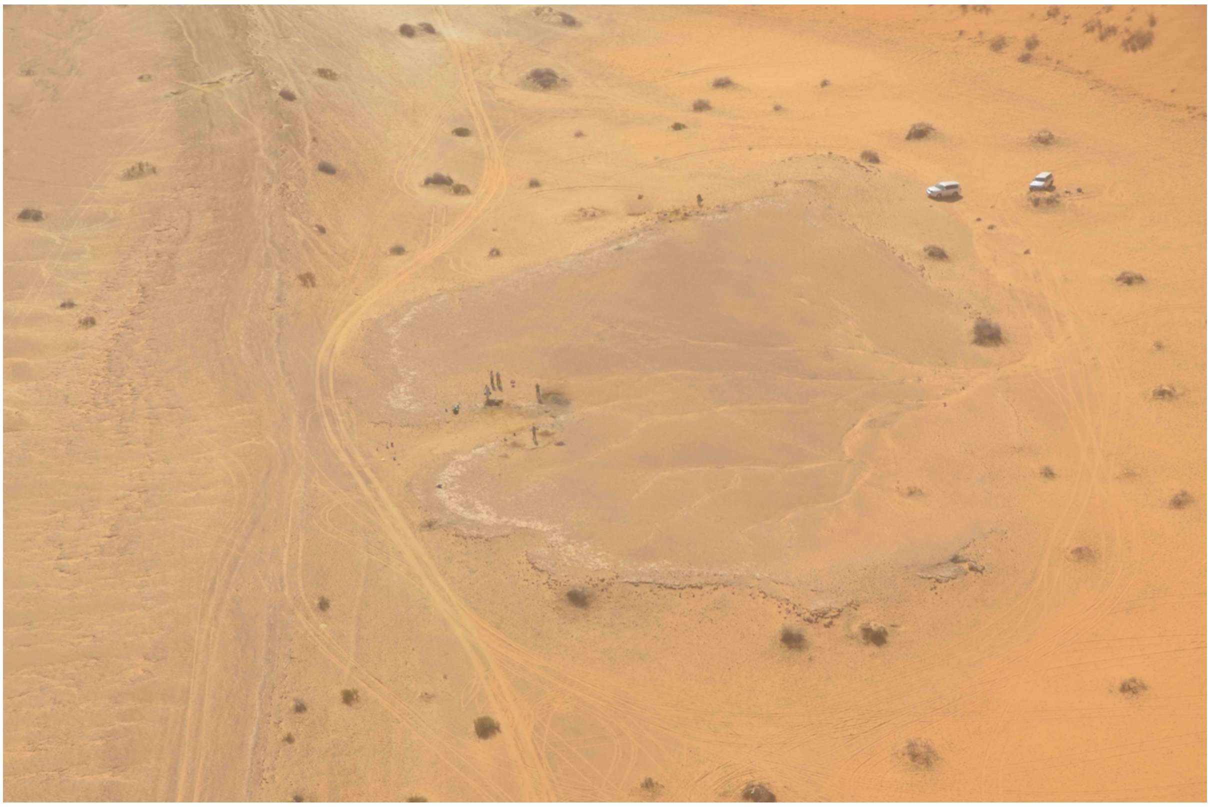

Supplementary figure 4. Aerial image of the northern part of KAM-4. Northwest Lake deposits between the team members and vehicles. Northeast Lake deposits on left.

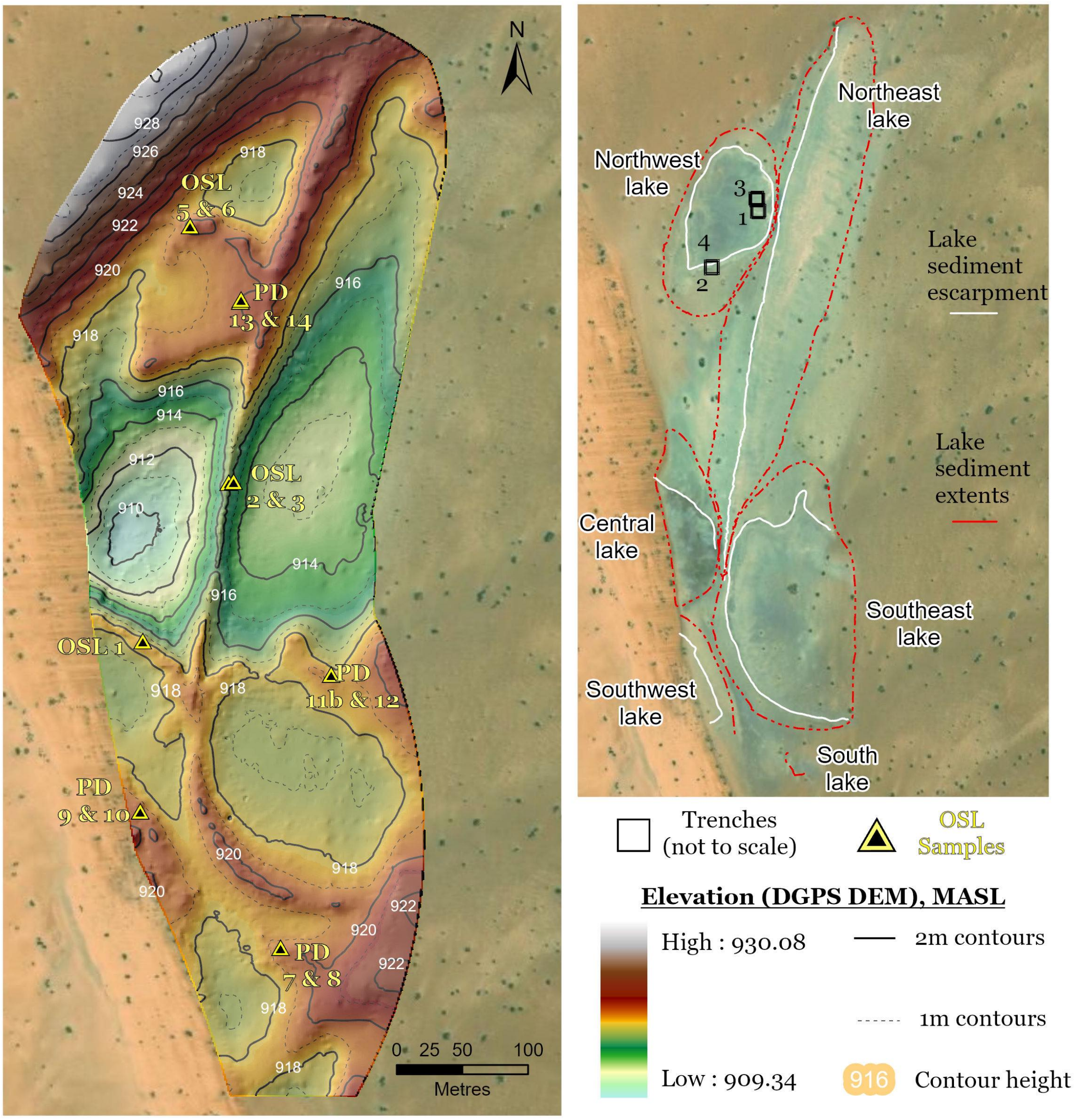

Supplementary figure 5. Digital elevation model of KAM-4 (left) and outlines of deposits assigned to different lake phases (right). Trenches, sections, and luminescence sample spots are also indicated. NB, U-series sample from trench 1, Northwest Lake.

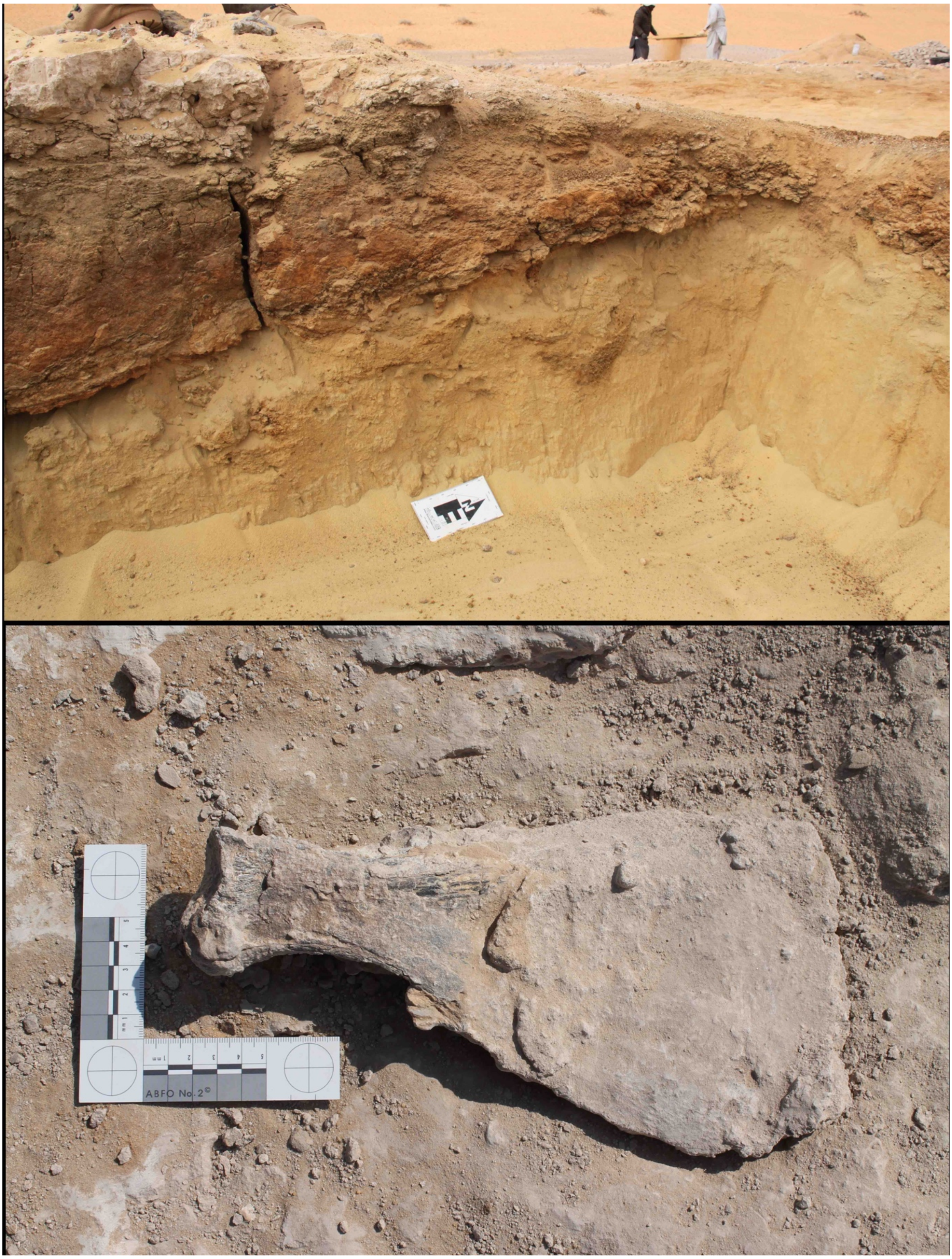

Supplementary figure 6. Top: trench 1 section in Northwest Lake showing steeply sloping sediments, where MIS 7 lacustrine deposits are draped on top of underlying aeolian sand. A sand bar/spit seems to have extended into the lake just to the right of this image, and fossils were focused around this point. Bottom: An example of one of the many fossils, in this case an *Oryx* scapula, found where the Unit 3 sediments met the surface.

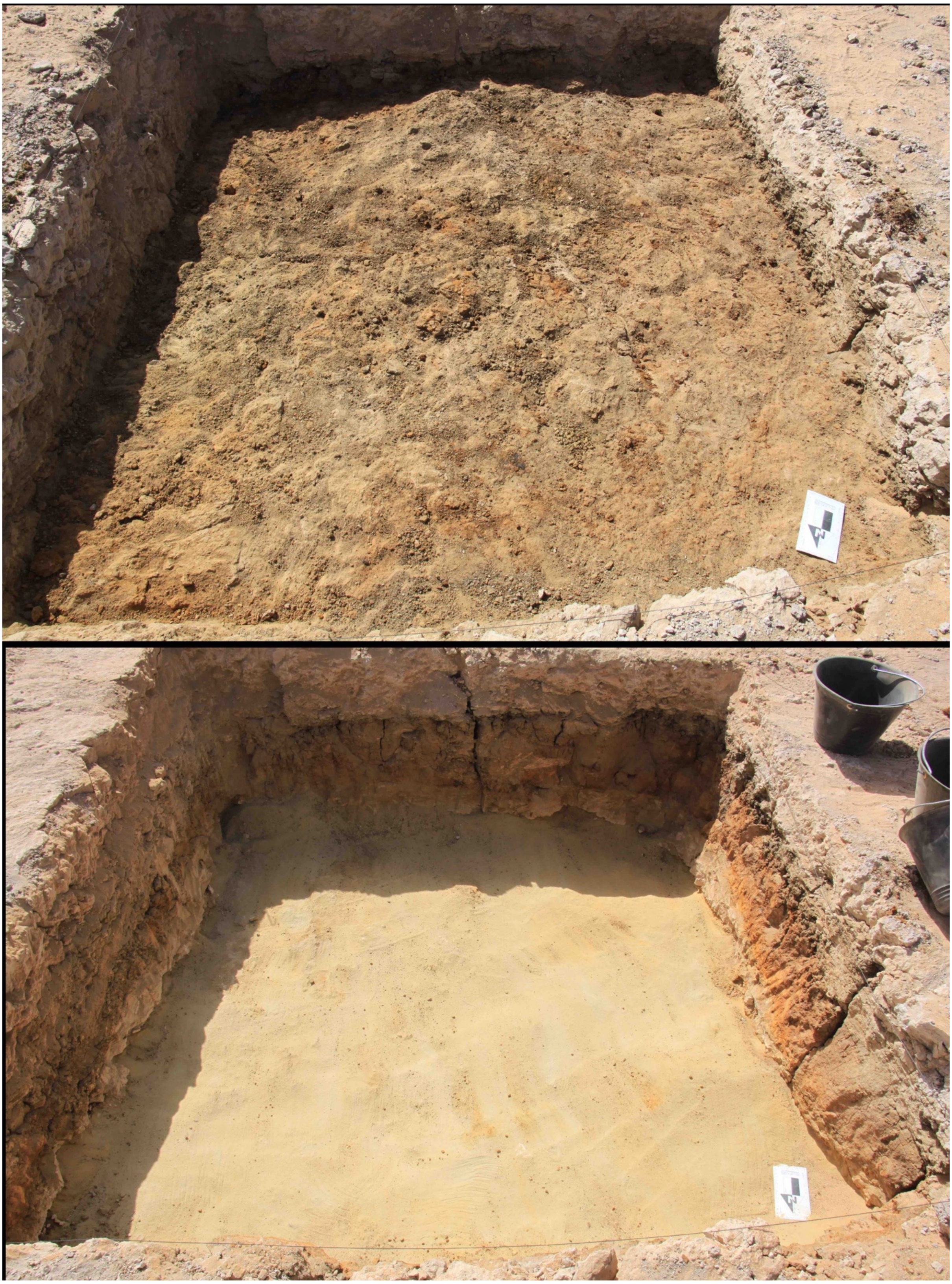

Supplementary figure 7. Example of excavated trench in Northwest Lake, KAM-4, where buried animal fossils were recovered. Top: during excavation of fossil bearing Unit 3 deposits. Bottom: underlying aeolian sands.

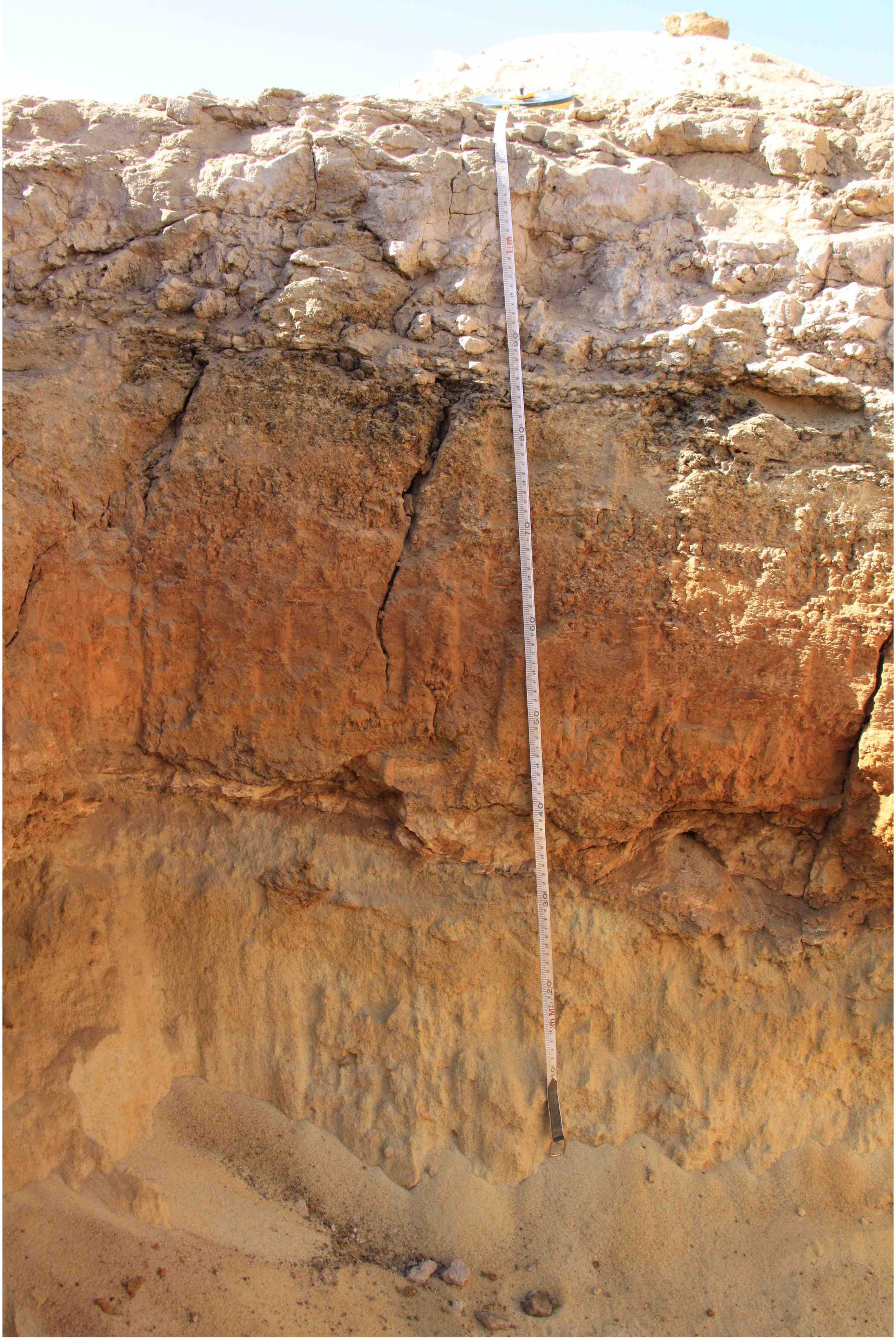

Supplementary figure 8. Example of stratigraphic section from KAM-4, Northwest Lake.

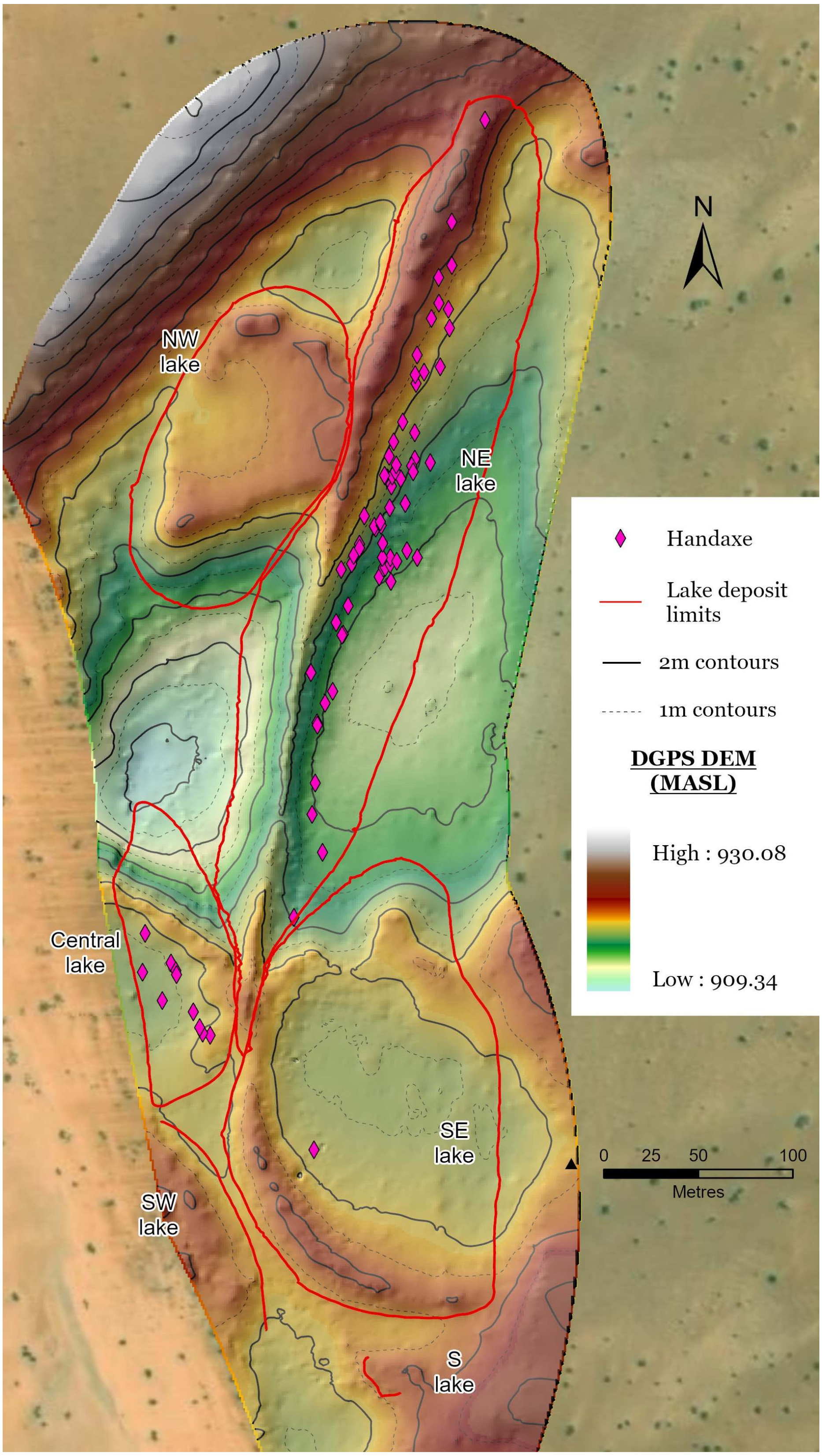

Supplementary figure 9. Distribution of handaxes at KAM-4.

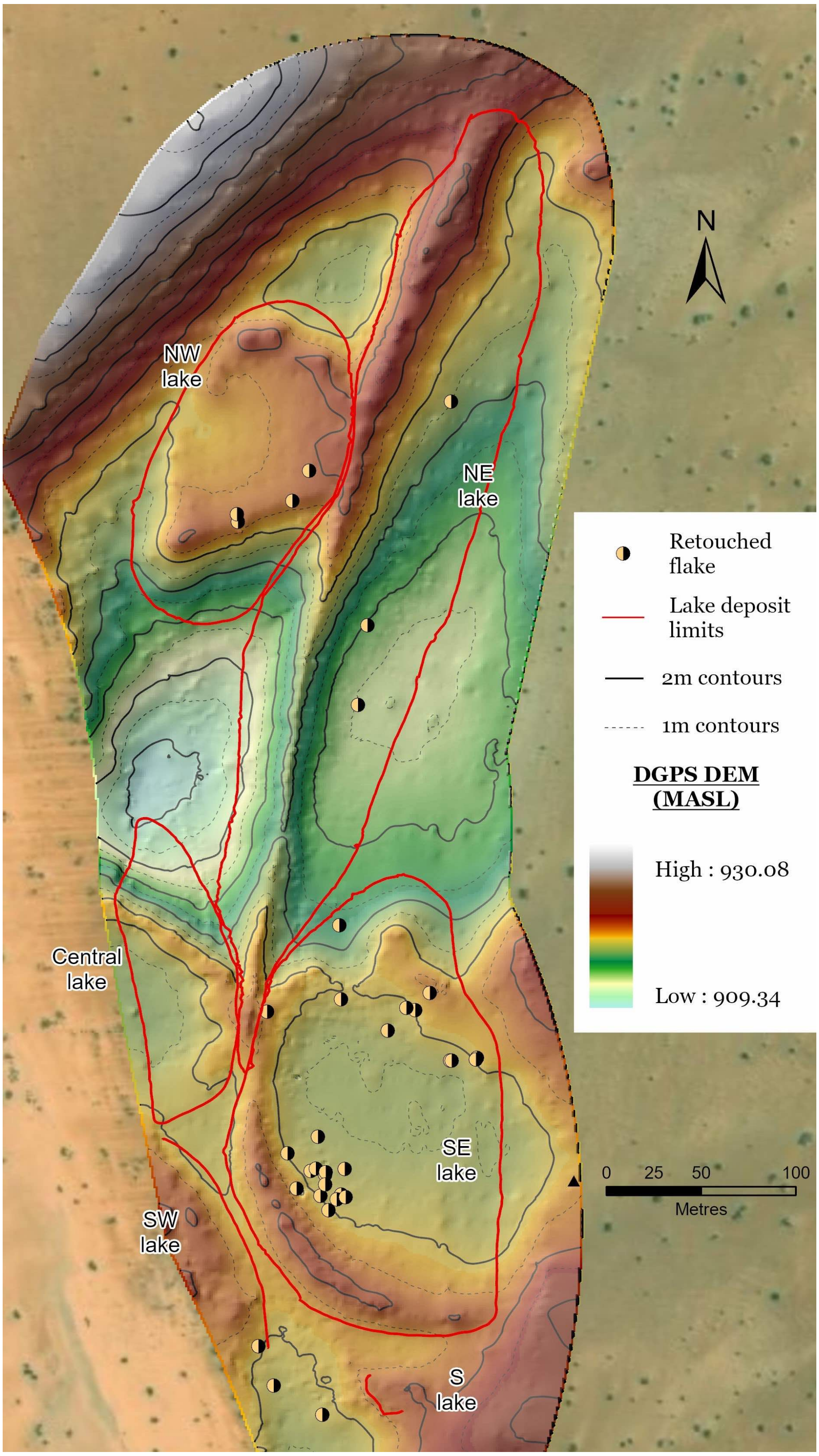

Supplementary figure 10. Distribution of retouched flakes at KAM-4.

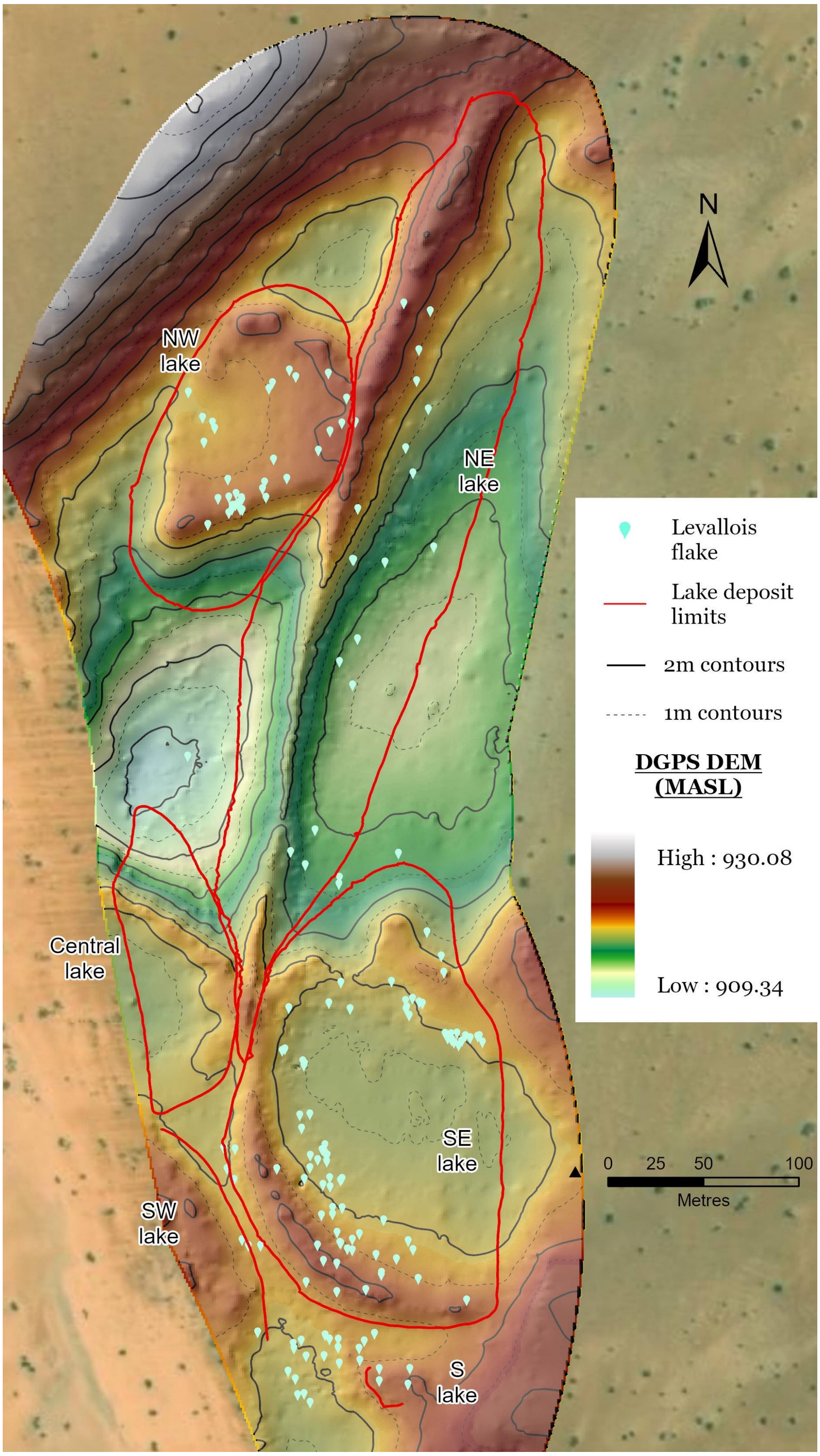

Supplementary figure 11. Distribution of Levallois flakes at KAM-4.

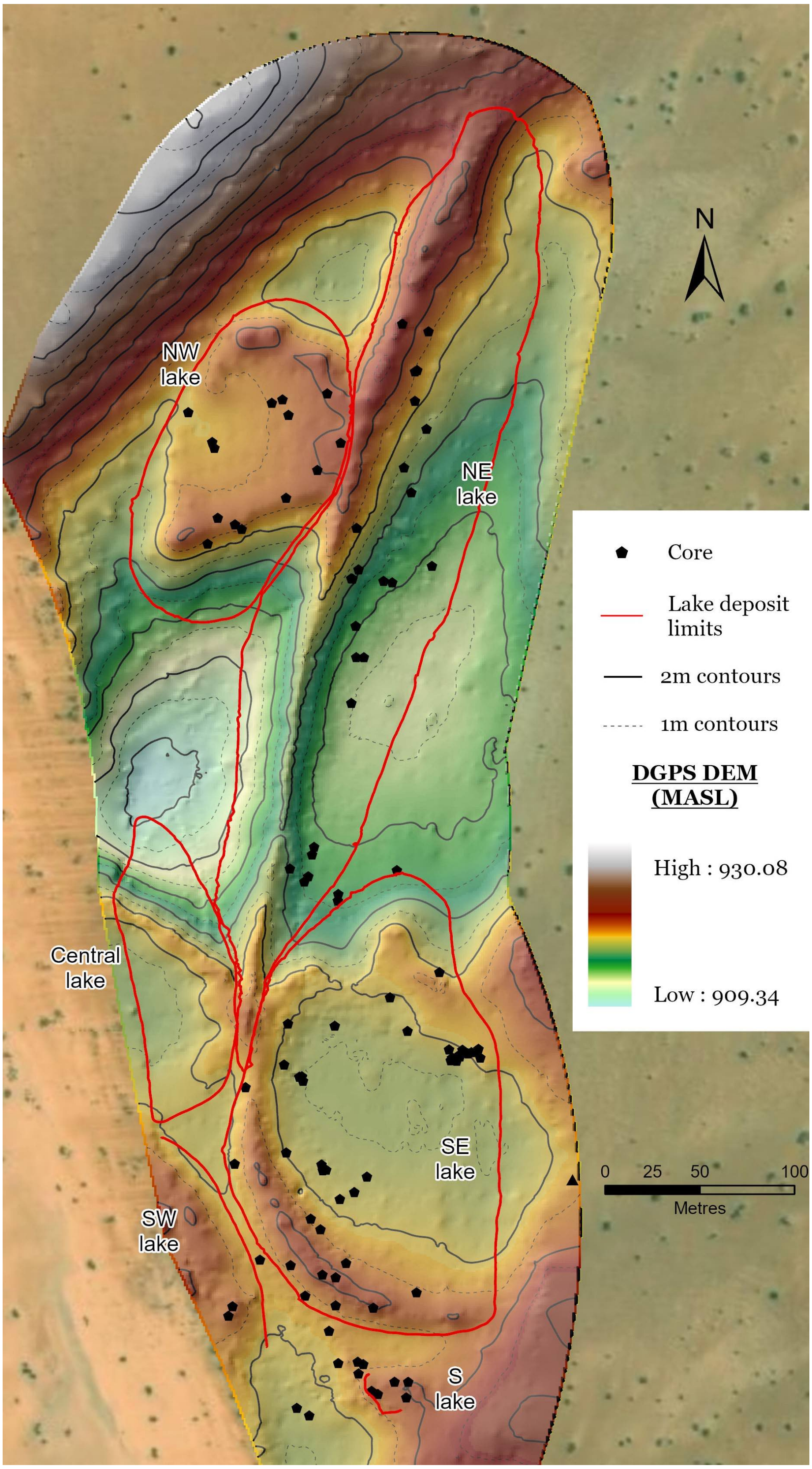

Supplementary figure 12. Distribution of cores at KAM-4.

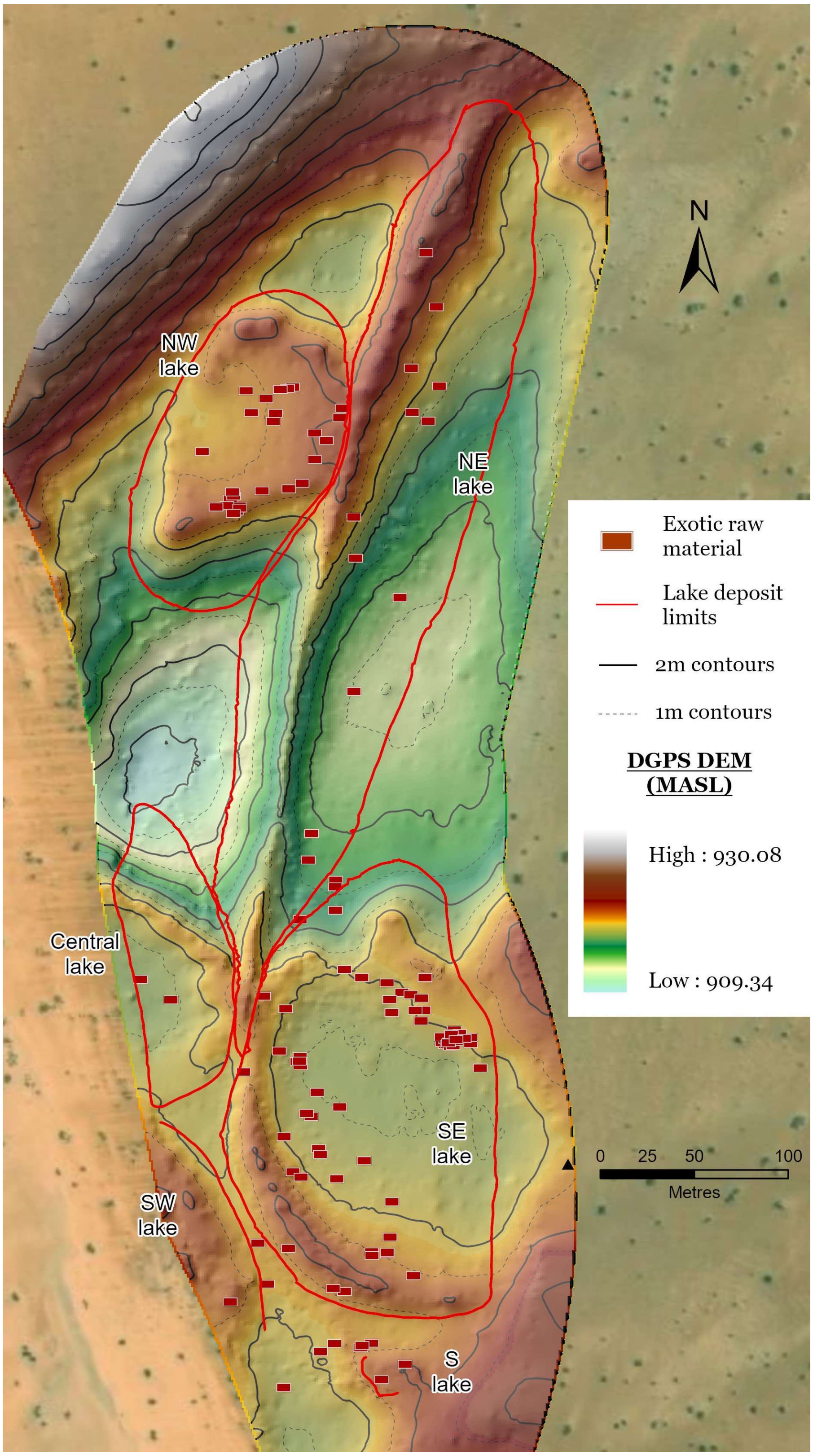

Supplementary figure 13. Distribution of lithics made on exotic raw materials at KAM-4.

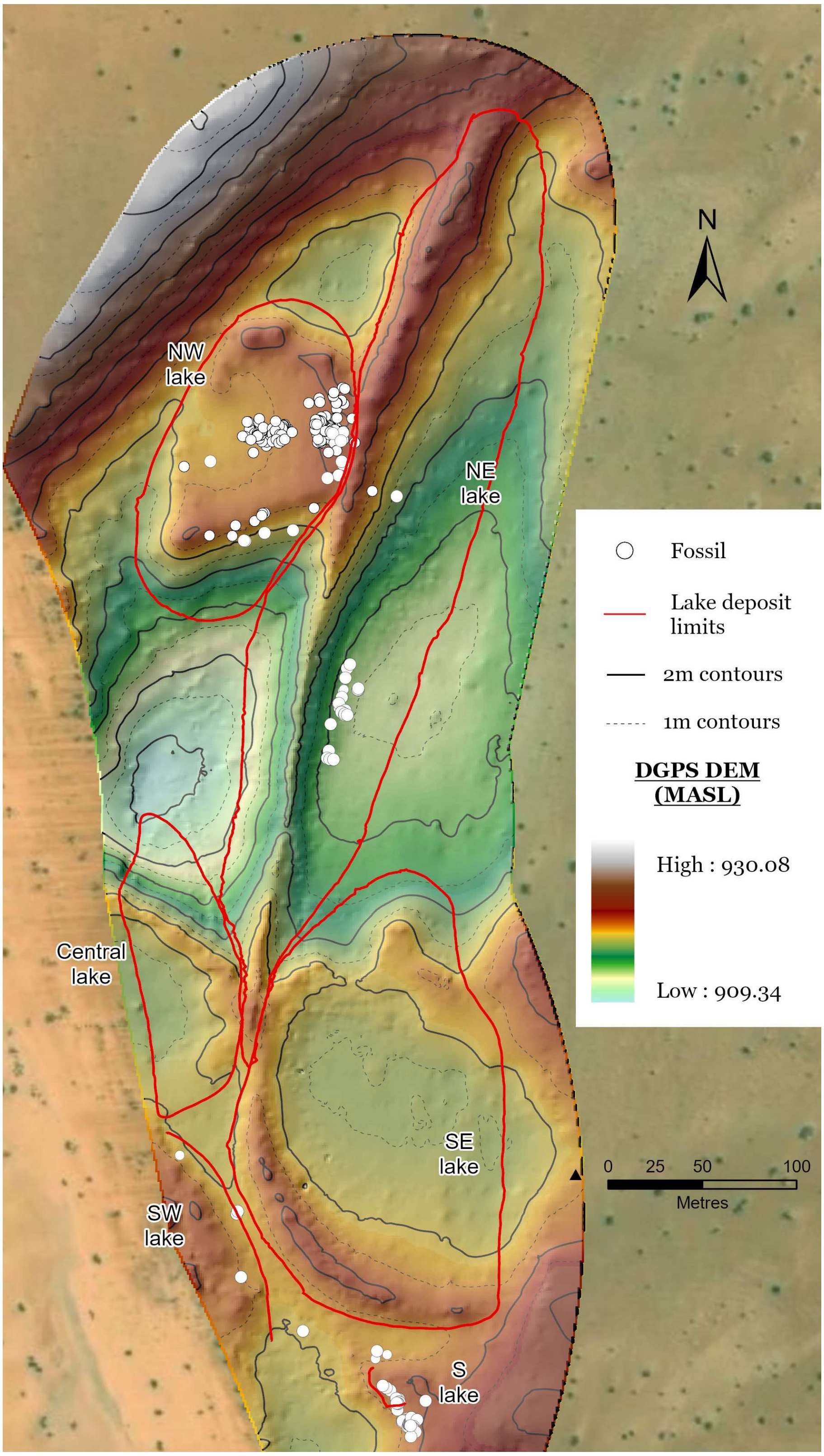

Supplementary figure 14. Distribution of animal fossils at KAM-4.

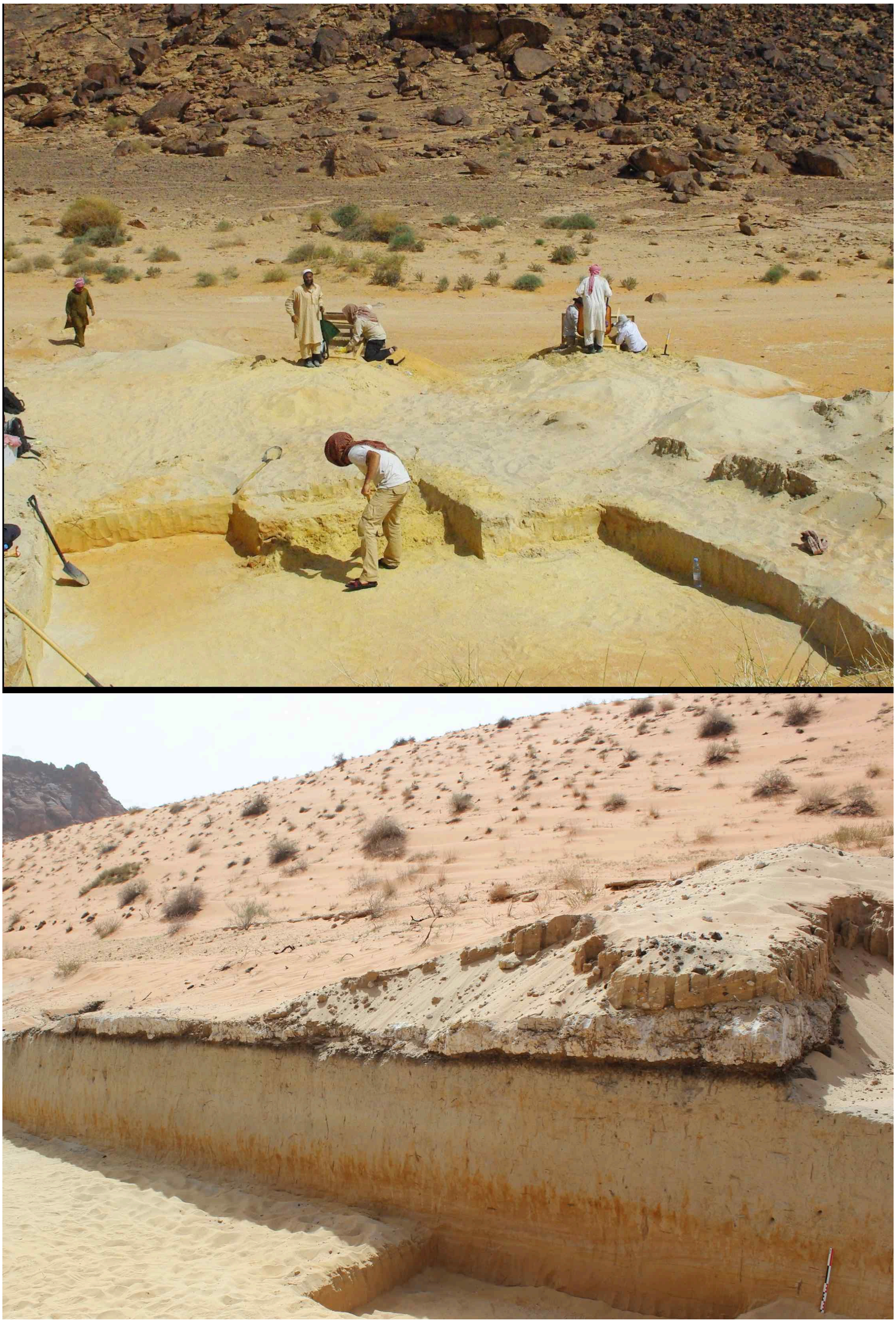

Supplementary figure 15. 2013 excavations at Jebel Qattar-1. Top: looking northeastwards during excavation of Trench B. Bottom: looking southeastwards at Trench A section at end of excavations.

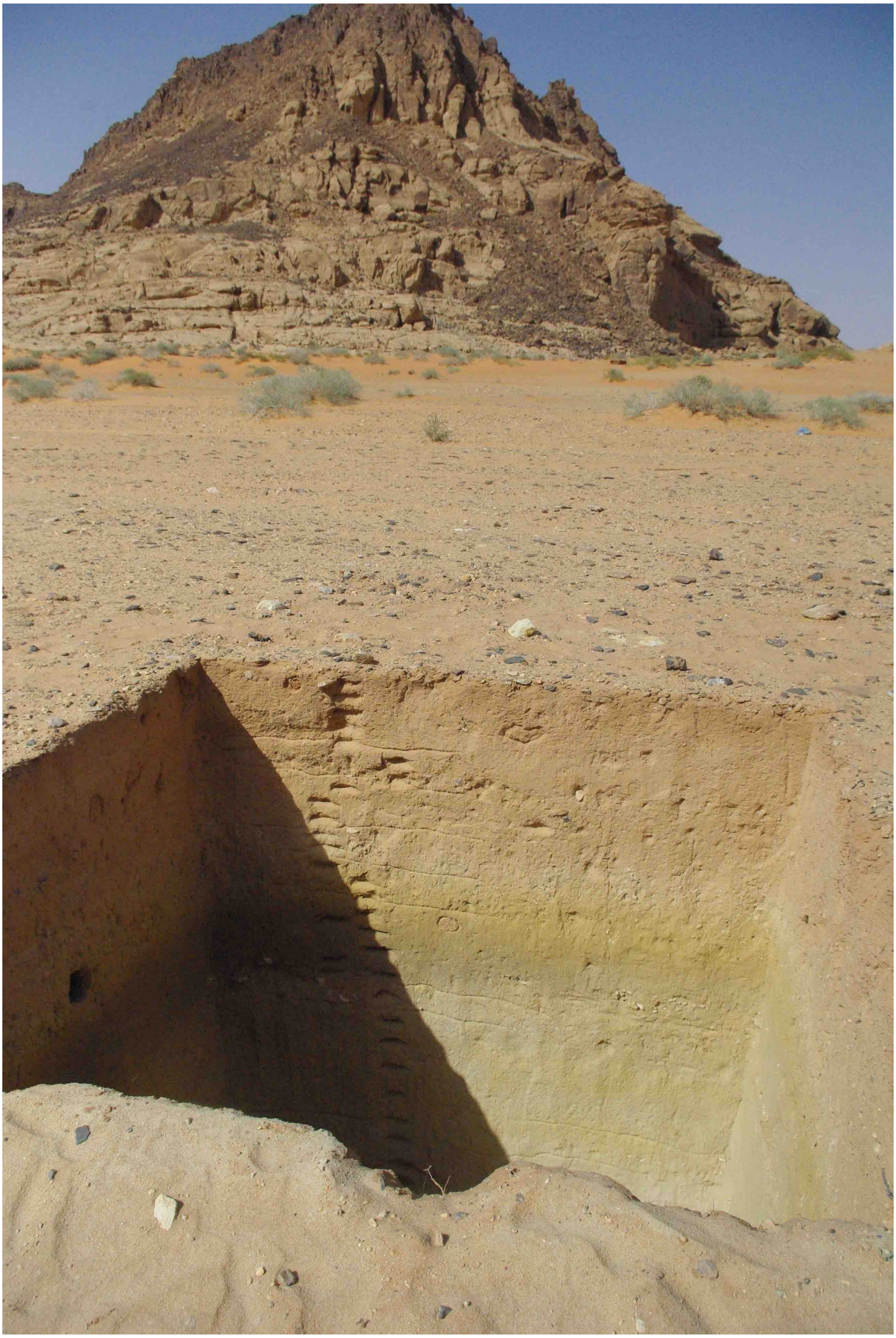

Supplementary figure 16. Trench 3 at JSM-1, looking uphill towards Jebel Umm Sanman.

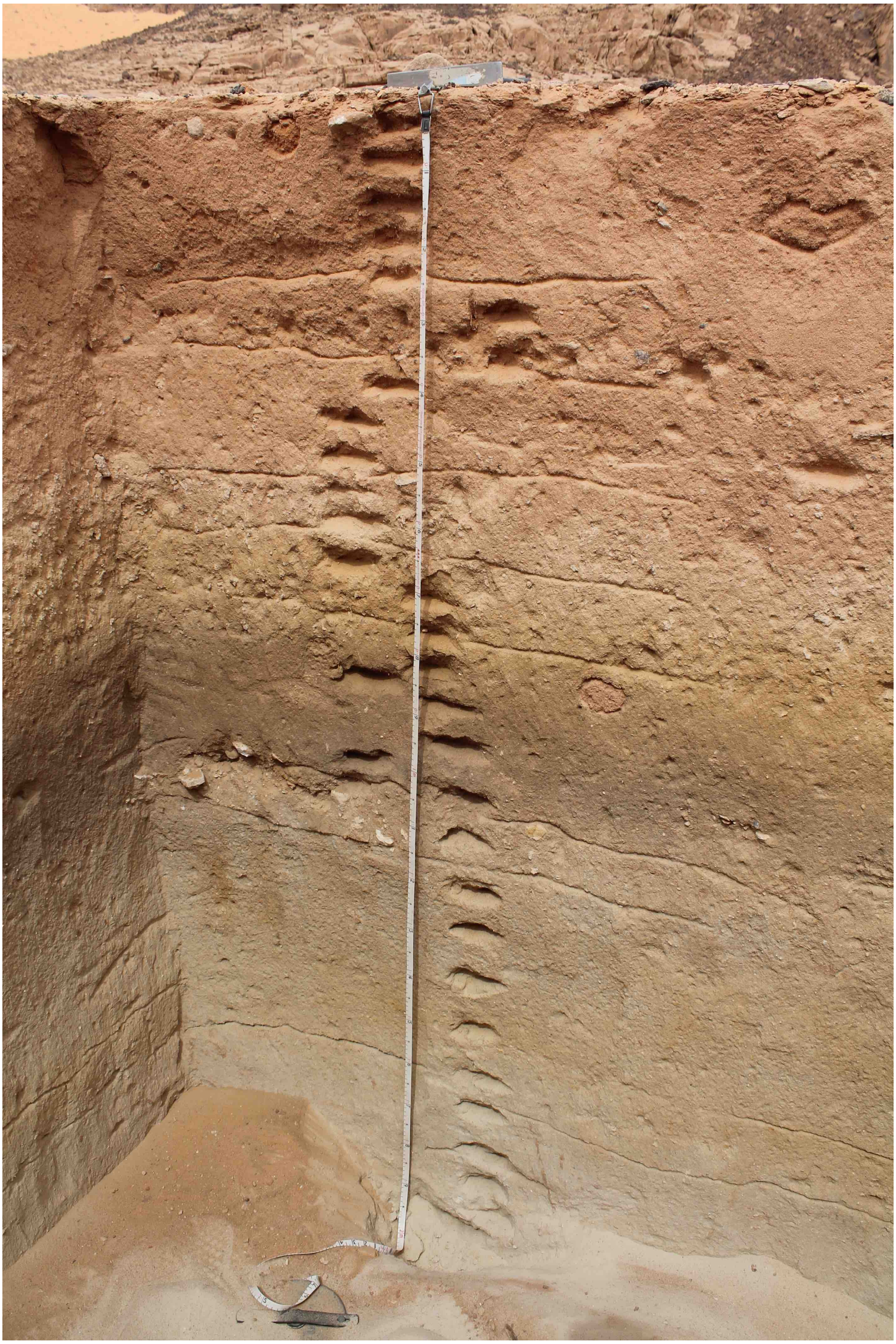

Supplementary figure 17. JSM-1 trench 3 section before OSL sampling.

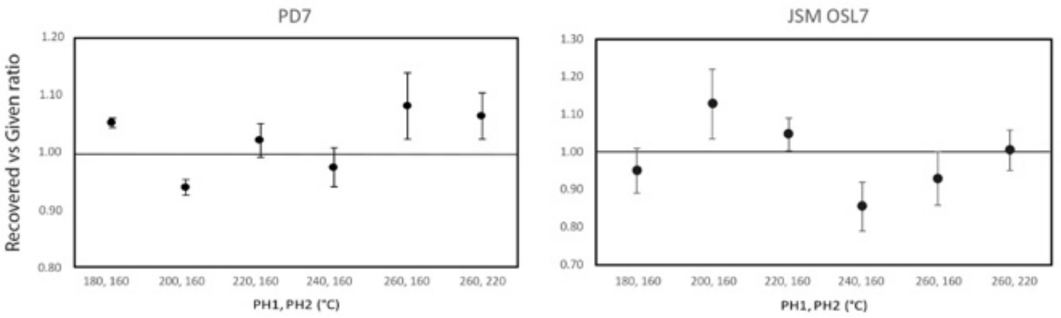

Supplementary figure 18. Preheat plateau dose recovery tests for PD7 and JSM-OSL7. A preheat of 240°C followed by a second preheat of 160°C was chosen for PD7, and 260°C/220°C for JSM-OSL7. Data are presented as mean values (n=3 in all cases) with 1σ uncertainties.

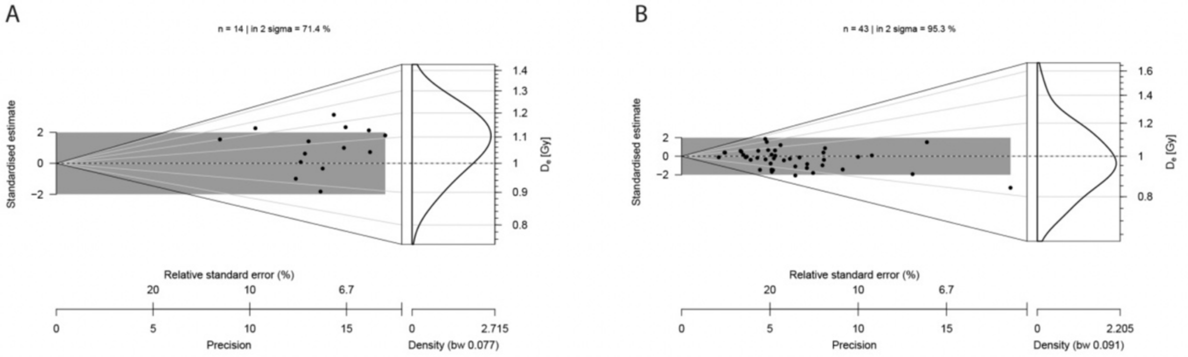

Supplementary figure 19. Dose recovery tests for the samples PD12 (A) and JSM-OSL12 (B). Given dose were ca. 94 Gy and ca. 103 Gy respectively. The dark grey rectangles represent the two-sigma distribution centred around unity.

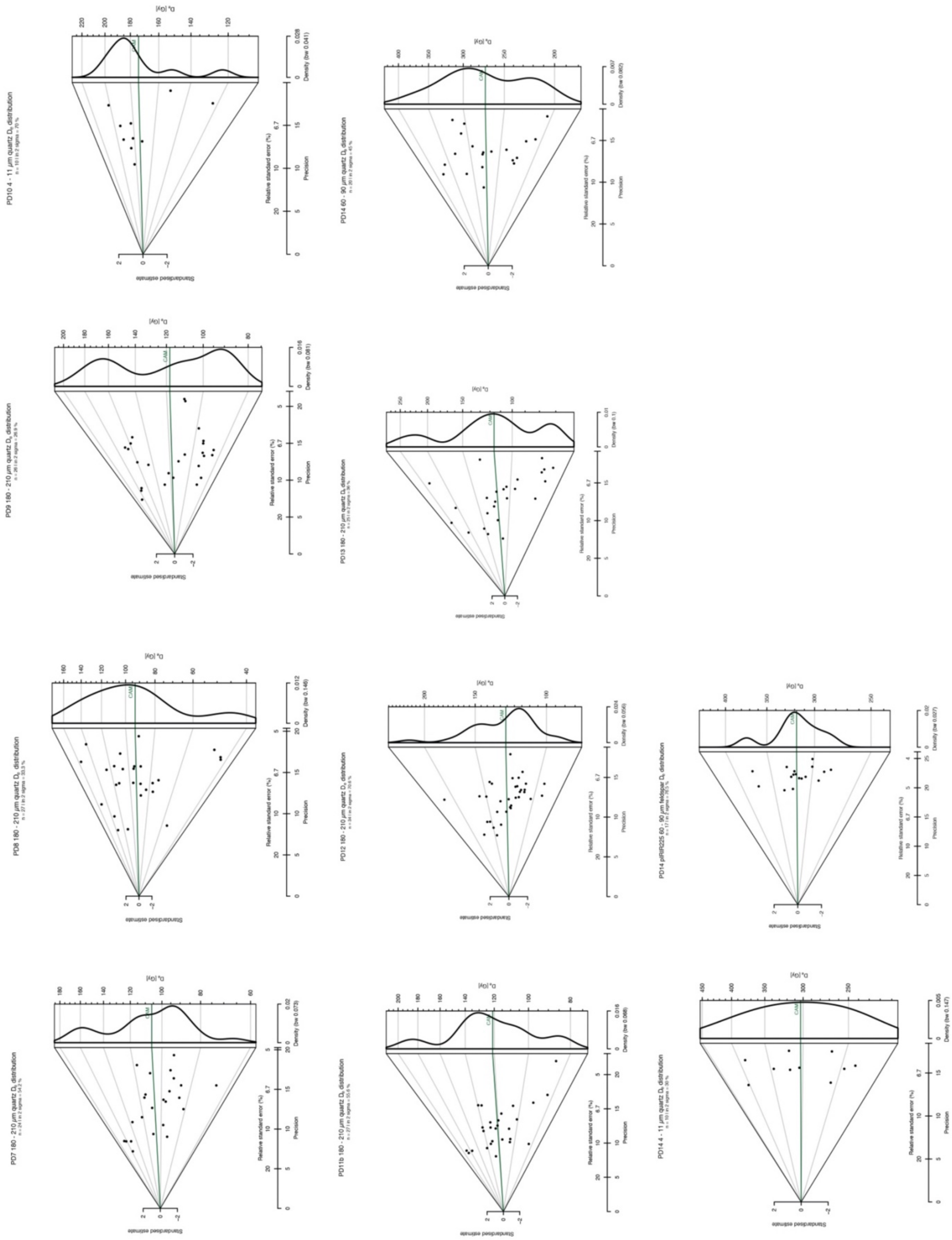

Supplementary figure 20a. Abanico plots of the De distributions extracted from the PD samples. Abanico plots are a combination of radial plots and Kernel Density Estimates. The dark green line represents the CAM De.

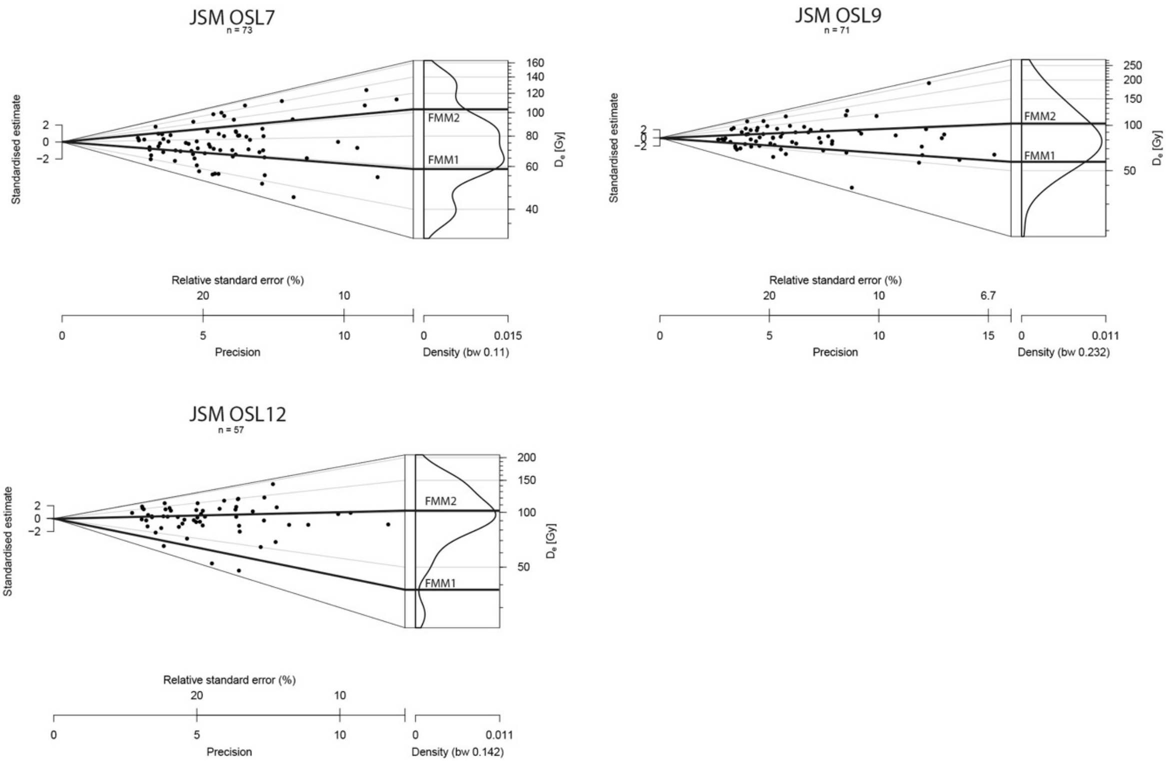

Supplementary figure 20b. Abanico plots of the De distributions extracted from the JSM-1 samples. The grey rectangles represent the FMM components.

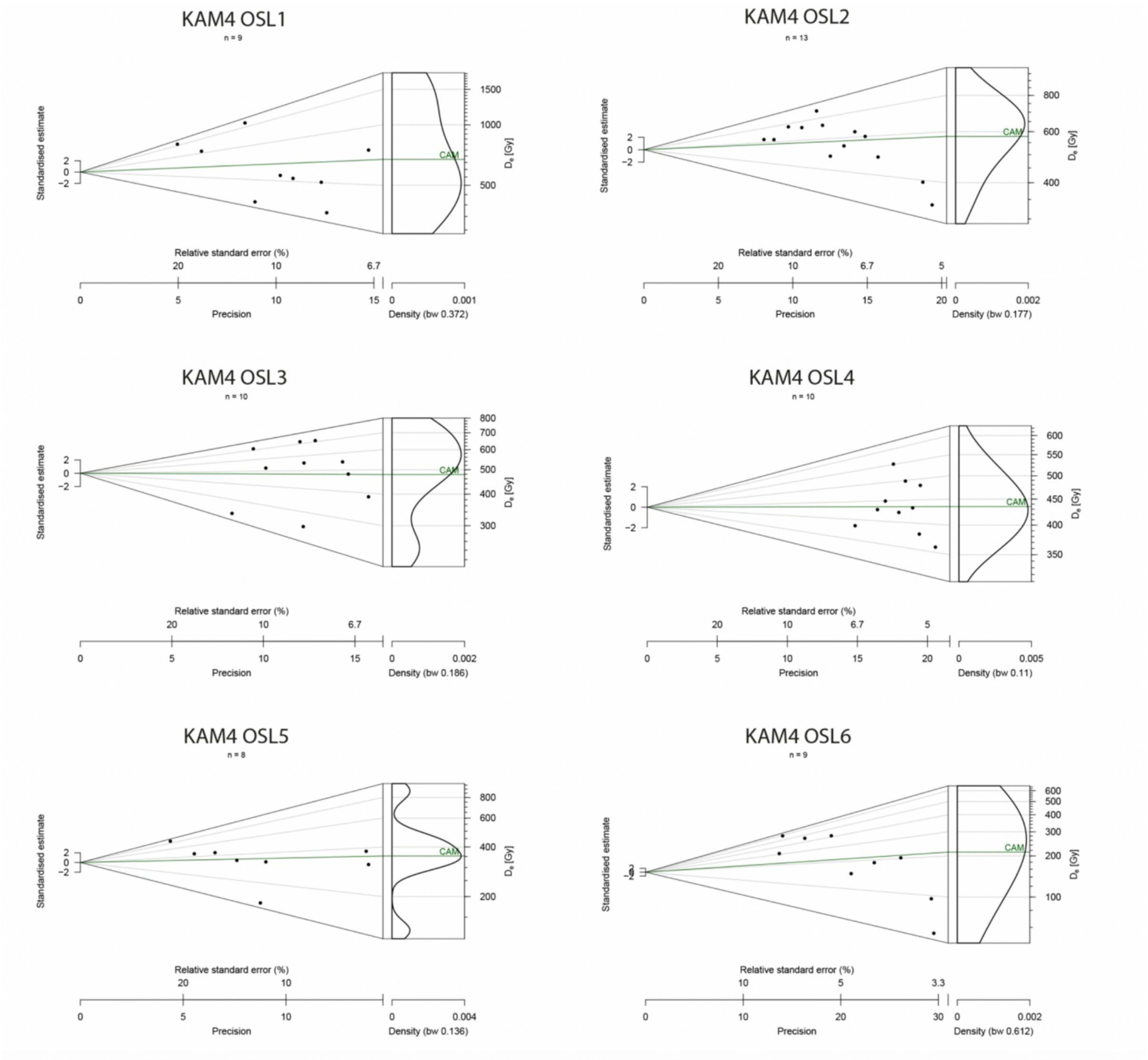

Supplementary figure 20c. Abanico plots of the De distributions extracted from the KAM-4 samples.

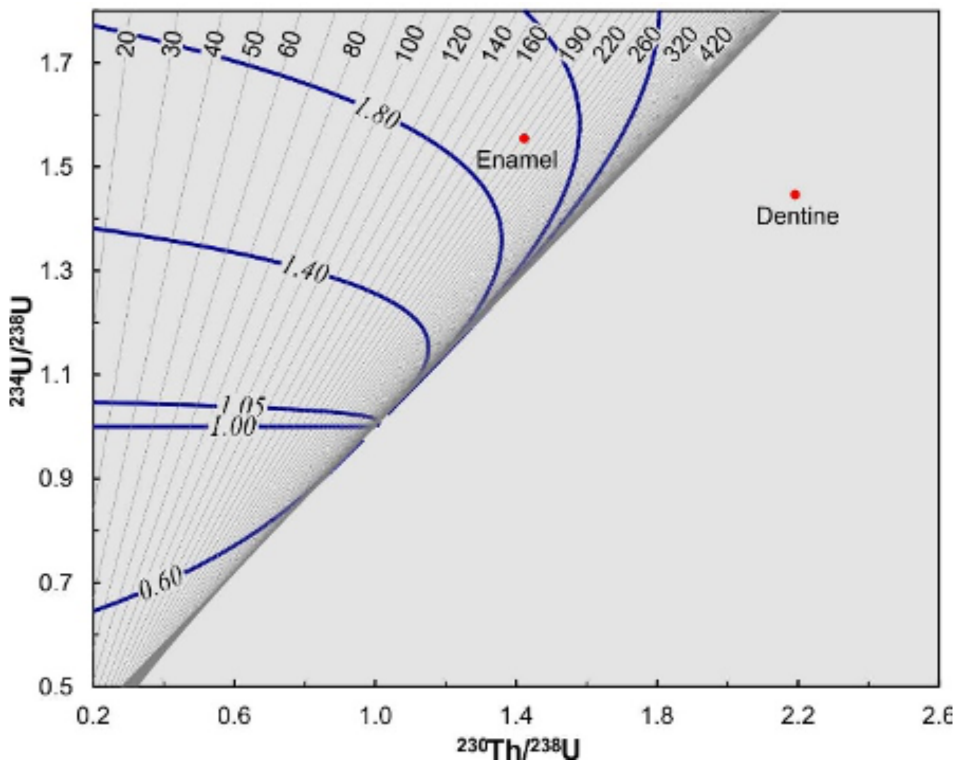

Supplementary figure 21. Isotope evolution diagram showing U-Th activity ratios for fossil tooth specimen KAM16/85. The dentine component lies in a region that cannot be reached had the tooth acted as a closed system after U uptake.

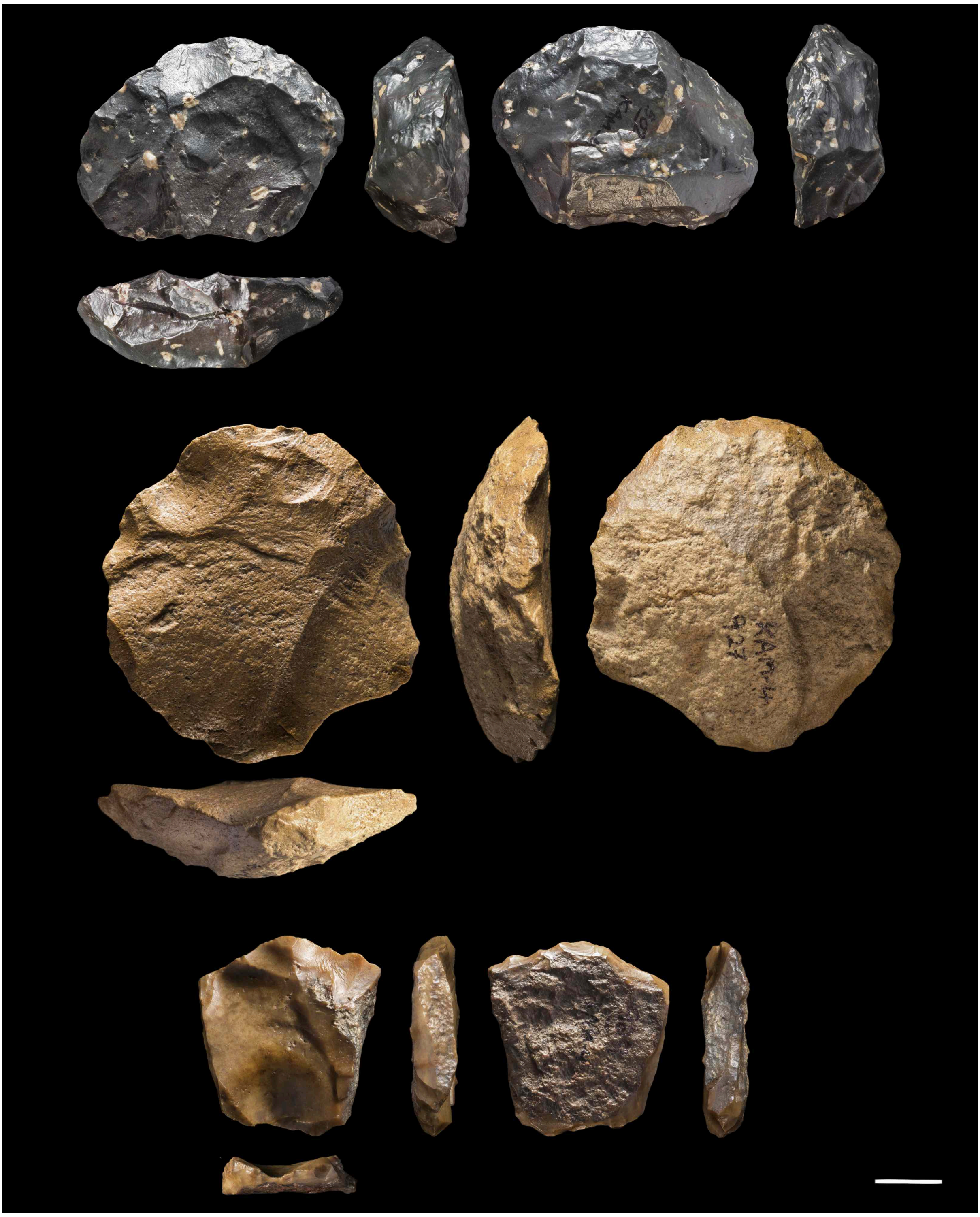

Supplementary figure 22. Examples of diverse raw materials used at KAM-4. All from Assemblage D, Southeast Lake (MIS 5?). Top: rhyolite. Middle: quartzite (non-ferruginous). Bottom: chert (probably of lacustrine origin).

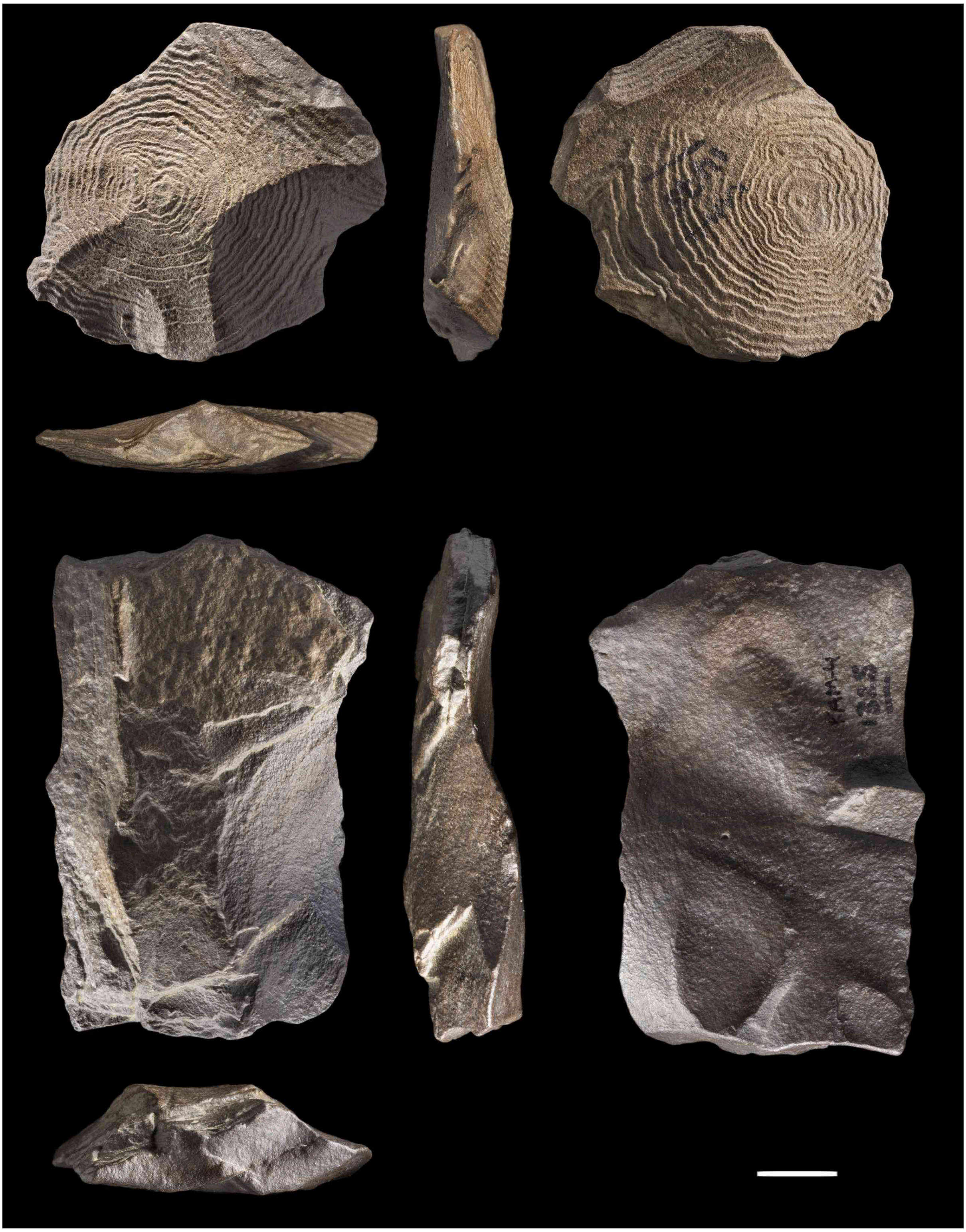

Supplementary figure 23. Illustration of different weathering of ferruginous quartzite at KAM-4. Top: flake from Assemblage C, Northwest Lake (MIS 7), bottom: flake from Assemblage E, South Lake (MIS 3?).

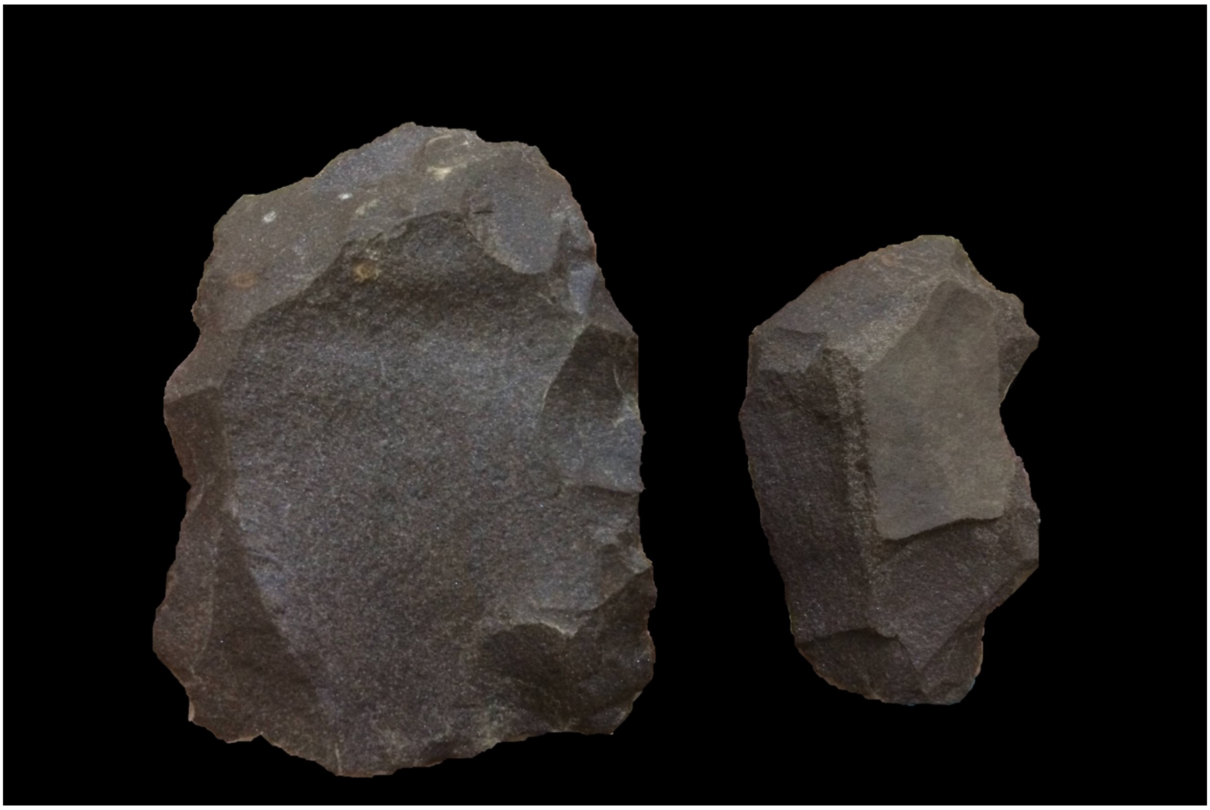

Supplementary figure 24. Example of experimental Levallois flake produced by HSG on the kind of ferruginous quartzite widely available in the area, and the most common raw material used at KAM-4.

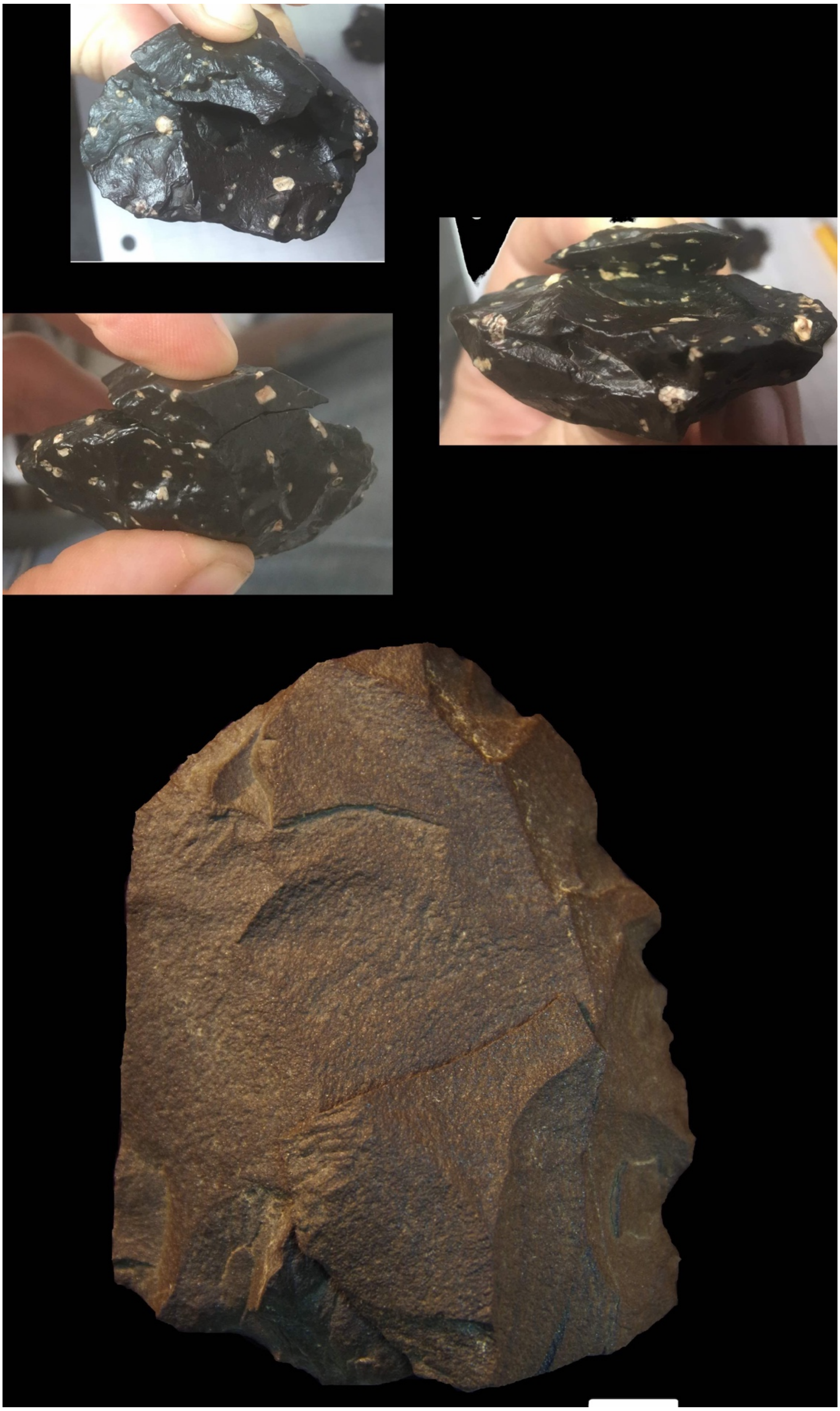

Supplementary figure 25. Examples of refits from KAM-4 (scale at bottom: 1 cm). Top: three views of rhyolite core preparation flake refitting to core (Assemblage D, South Lake). Bottom: proximal part of broken preferential Levallois flake refitting to core (Assemblage C, Northwest Lake).

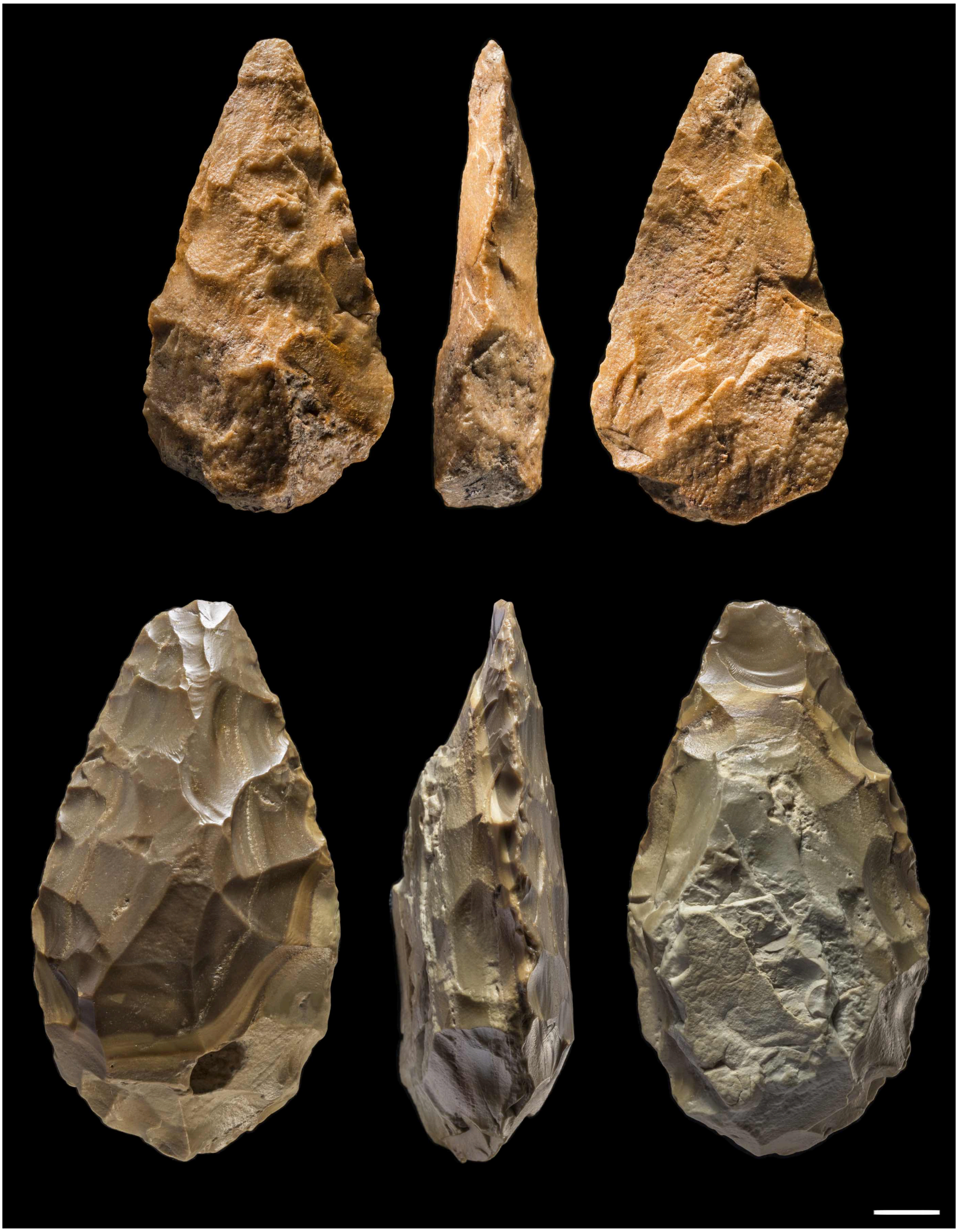

Supplementary figure 26. Examples of previously identified handaxes from the western Nefud Desert, from the site of Khabb Musayyib 2. (scale: 1 cm).

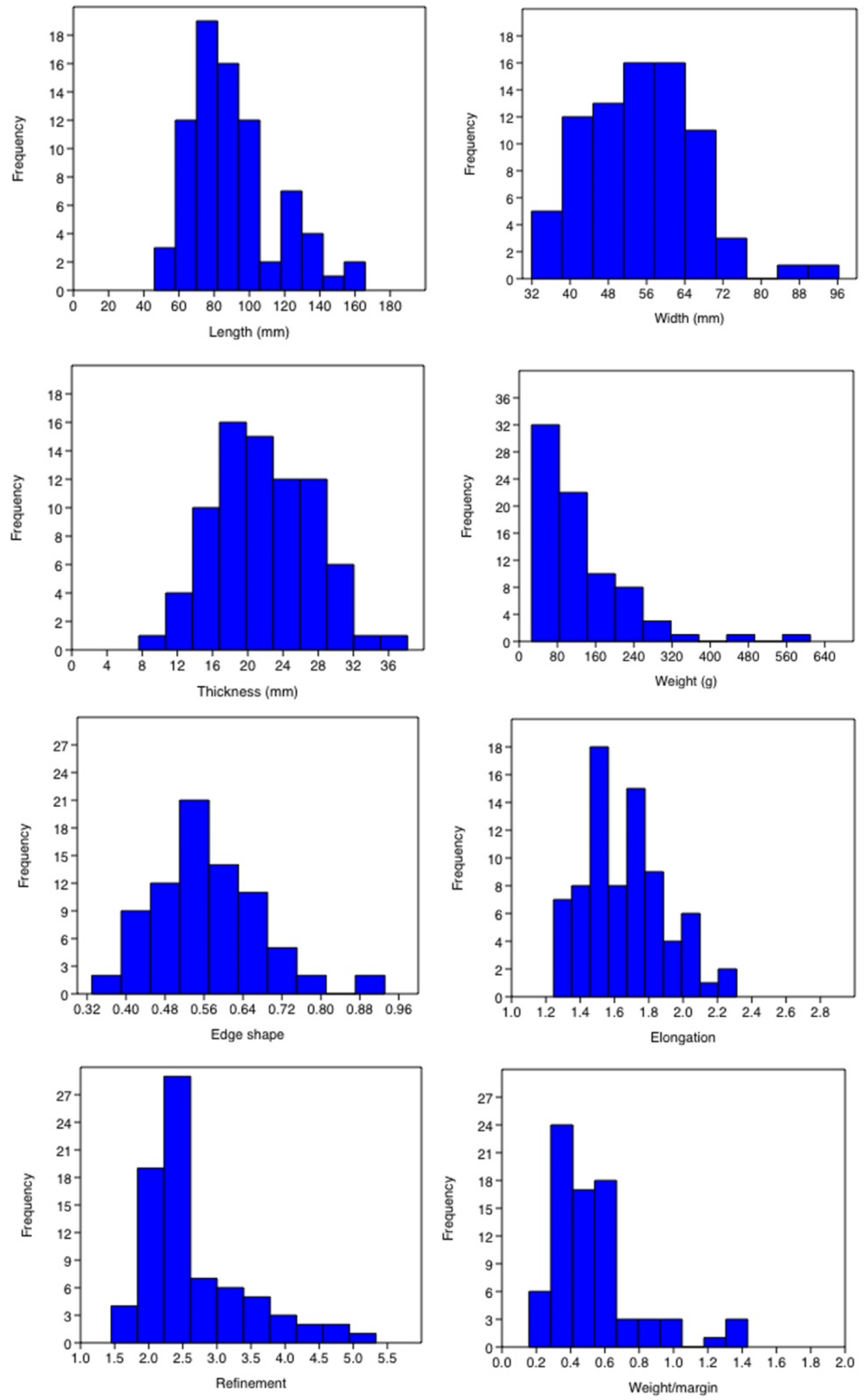

Supplementary figure 27. Histograms of size and shape data for Assemblage B bifaces, KAM-4. See text for definition of terms, and discussion.

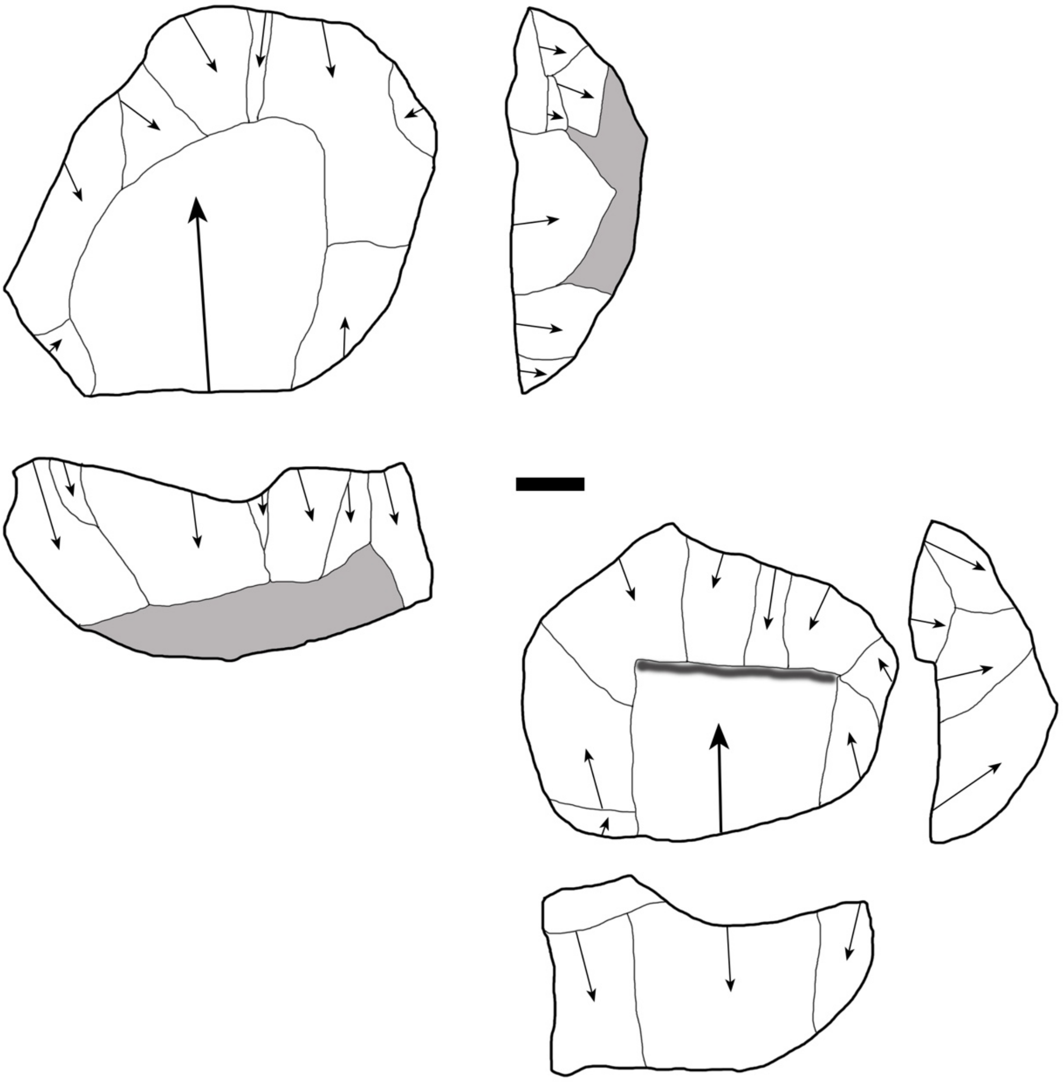

Supplementary figure 28. Preferential Levallois cores from Assemblage B, KAM-4. Scale: 1cm, grey: cortex, thick arrow: Levallois removal.

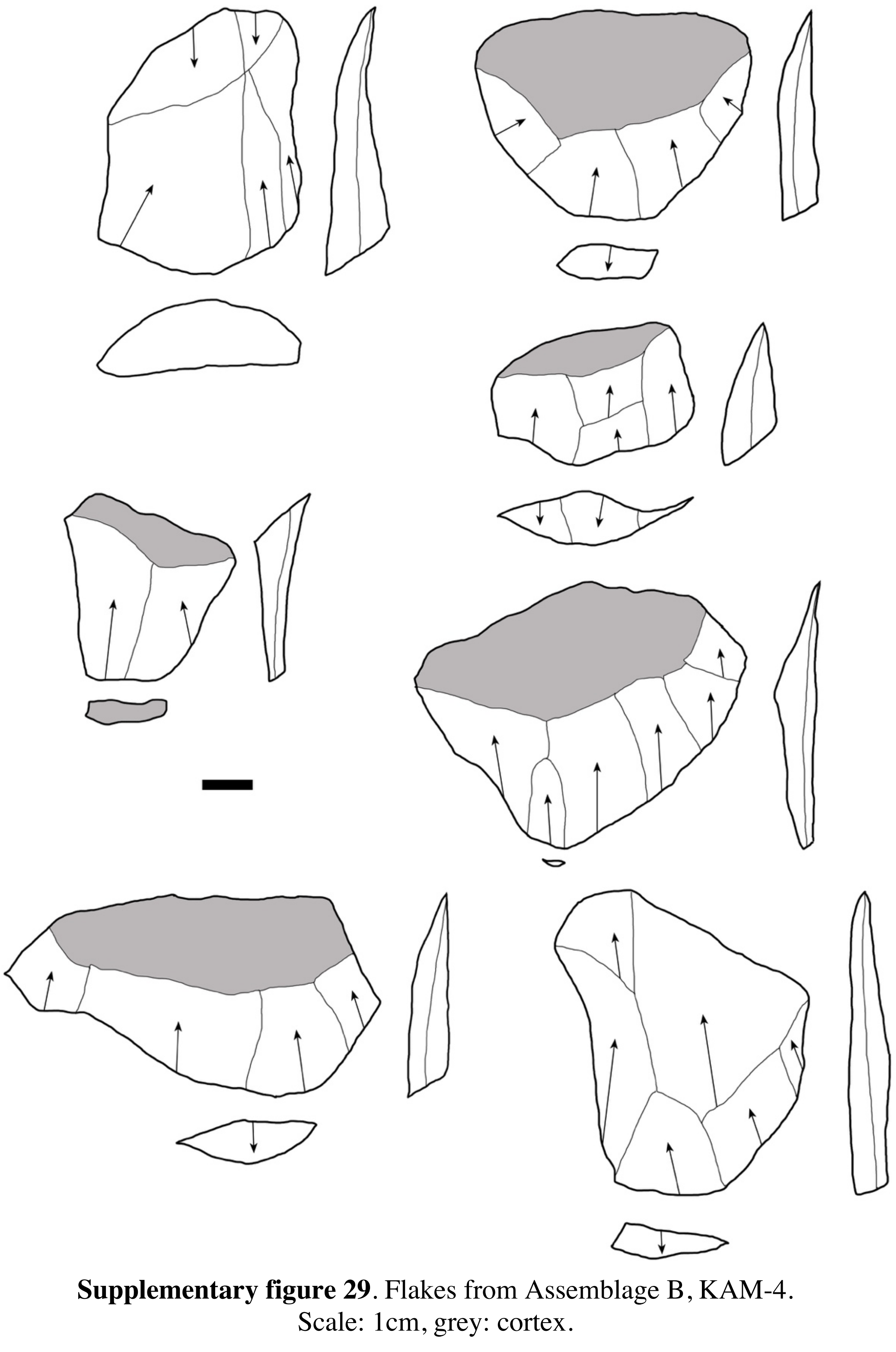

Supplementary figure 29. Flakes from Assemblage B, KAM-4.
Scale: 1cm, grey: cortex.

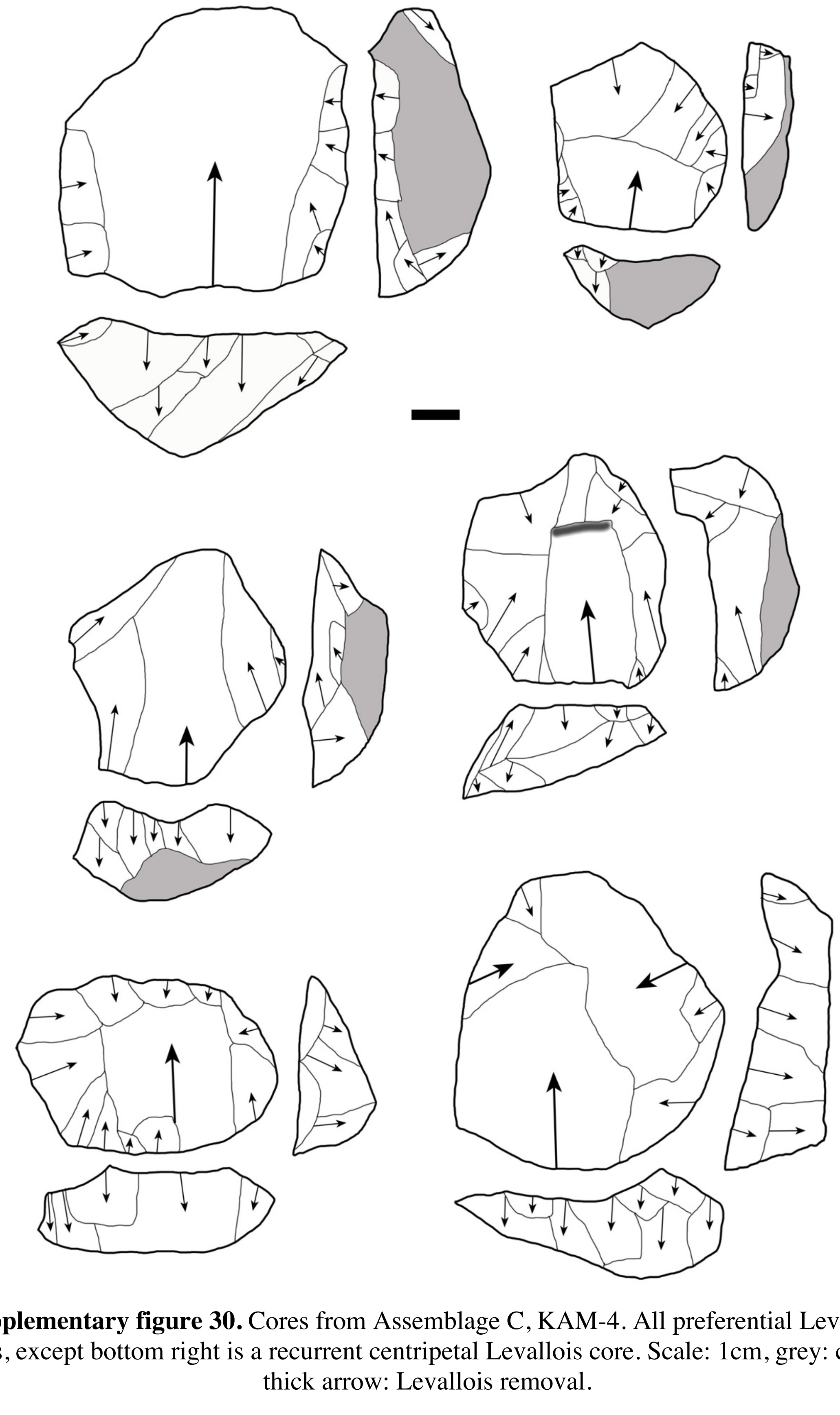

Supplementary figure 30. Cores from Assemblage C, KAM-4. All preferential Levallois cores, except bottom right is a recurrent centripetal Levallois core. Scale: 1cm, grey: cortex, thick arrow: Levallois removal.

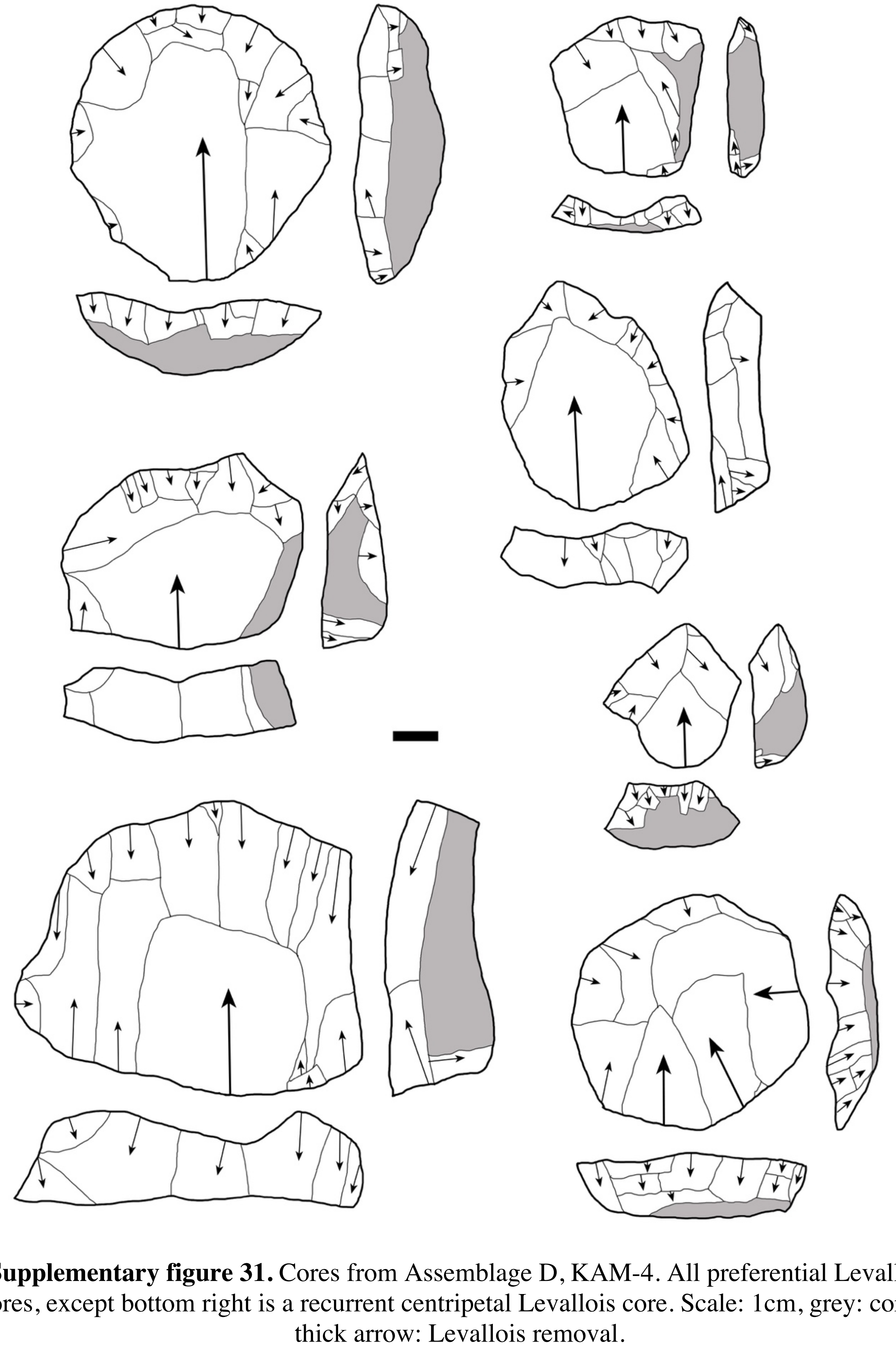

Supplementary figure 31. Cores from Assemblage D, KAM-4. All preferential Levallois cores, except bottom right is a recurrent centripetal Levallois core. Scale: 1cm, grey: cortex, thick arrow: Levallois removal.

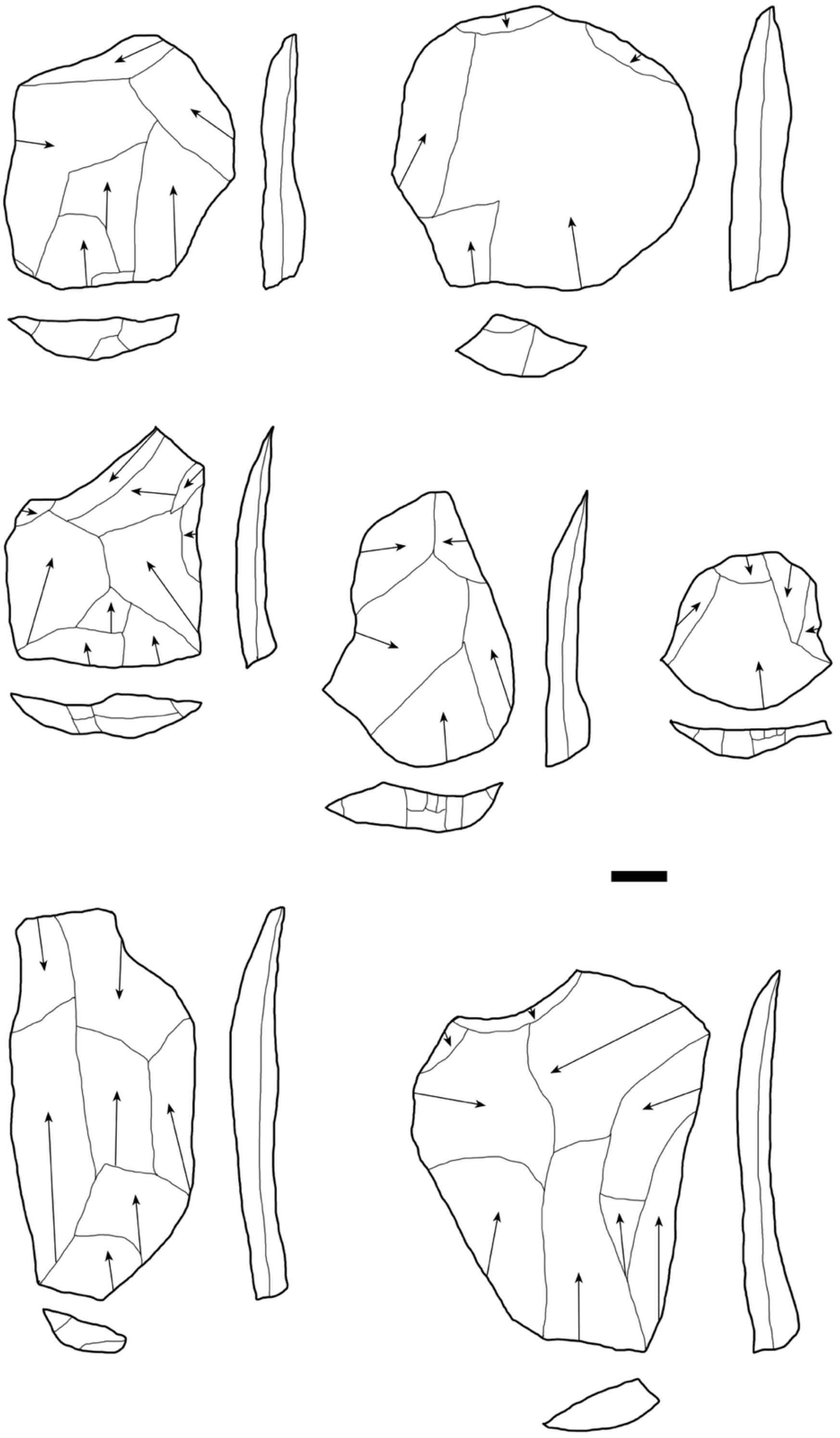

Supplementary figure 32. Levallois flakes from Assemblage D, KAM-4. Scale: 1cm.

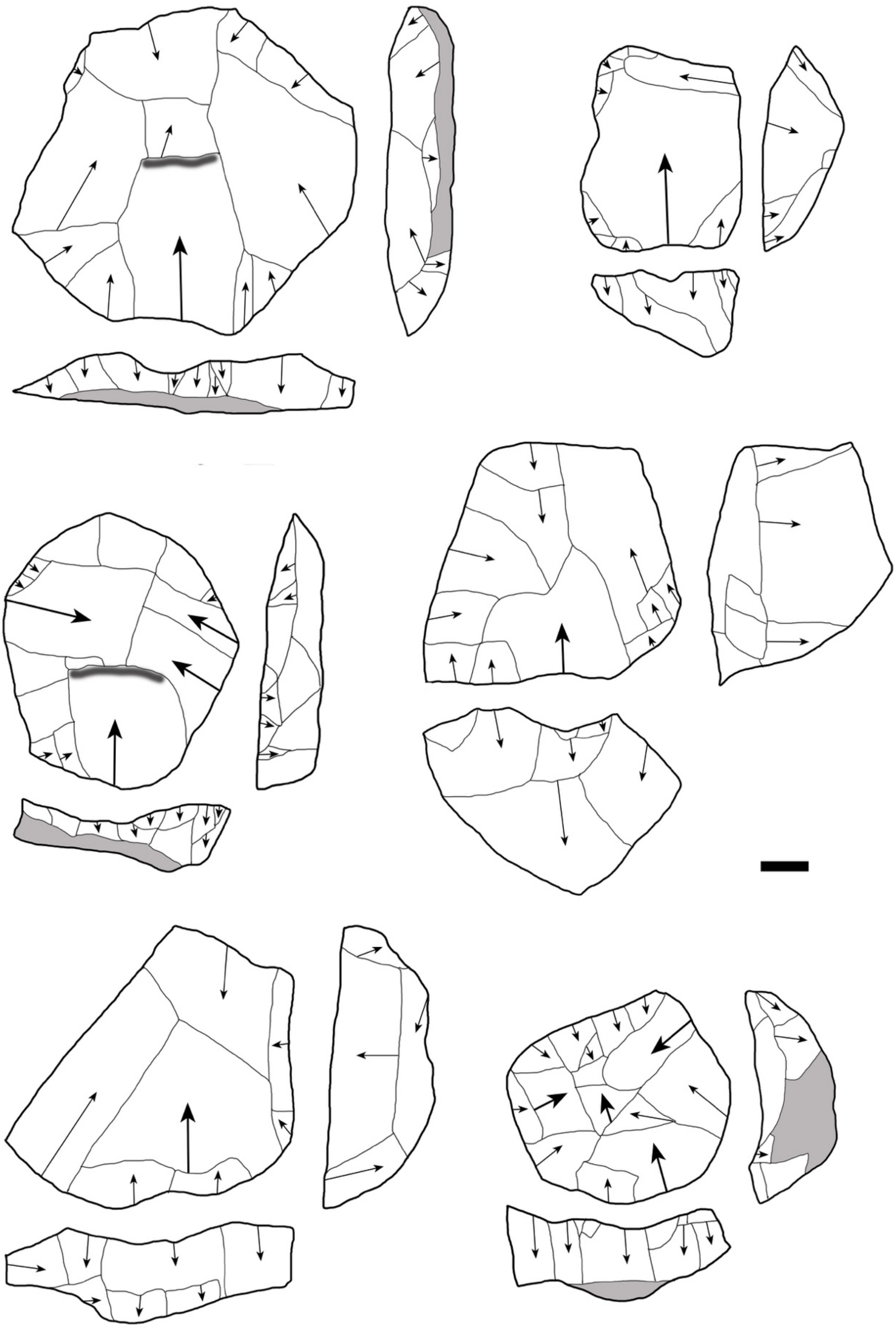

Supplementary figure 33. Cores from Assemblage E, KAM-4. Scale: 1cm, grey: cortex, thick arrow: Levallois removal.

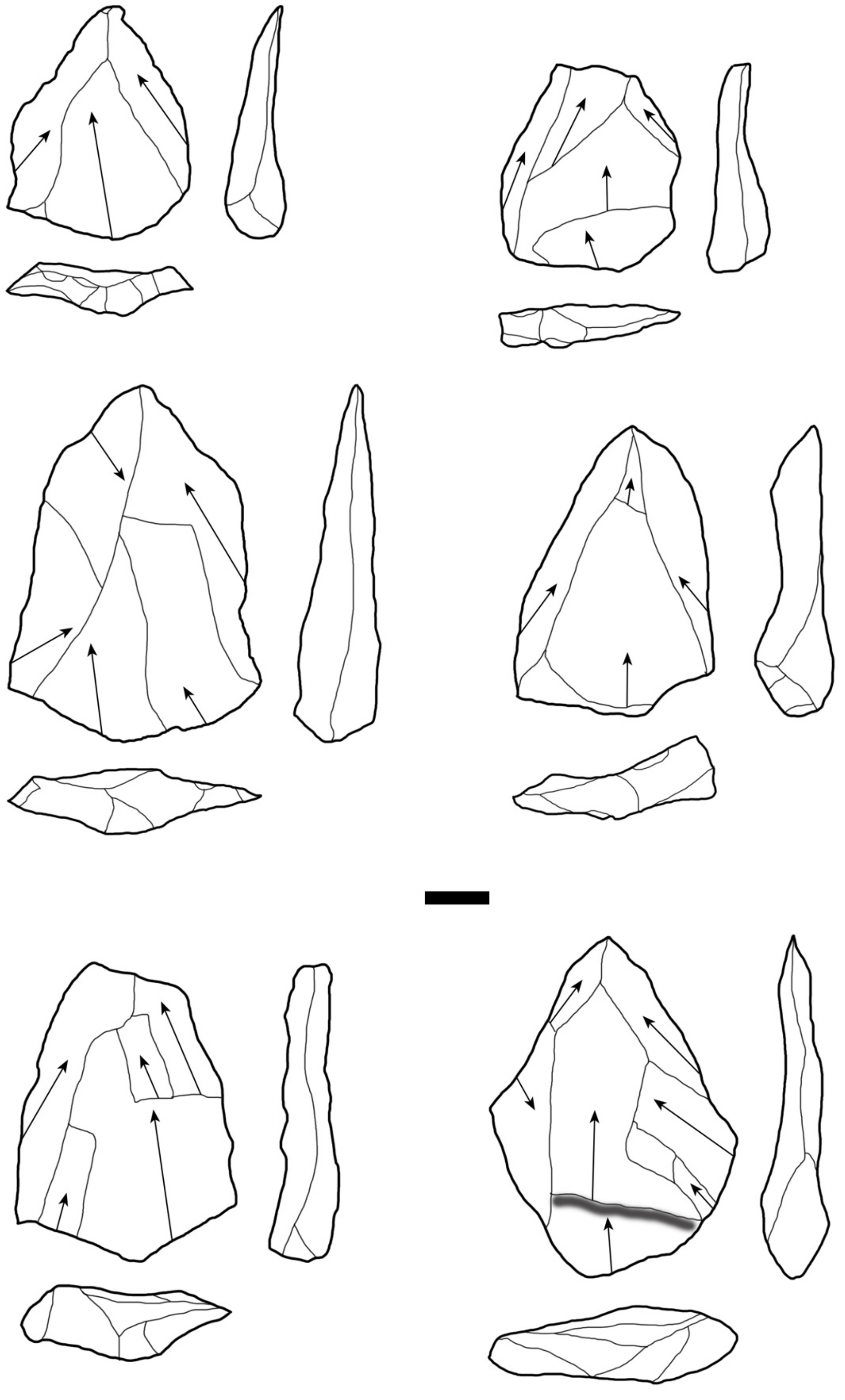

Supplementary figure 34. Levallois flakes from Assemblage E, KAM-4. Scale: 1cm.

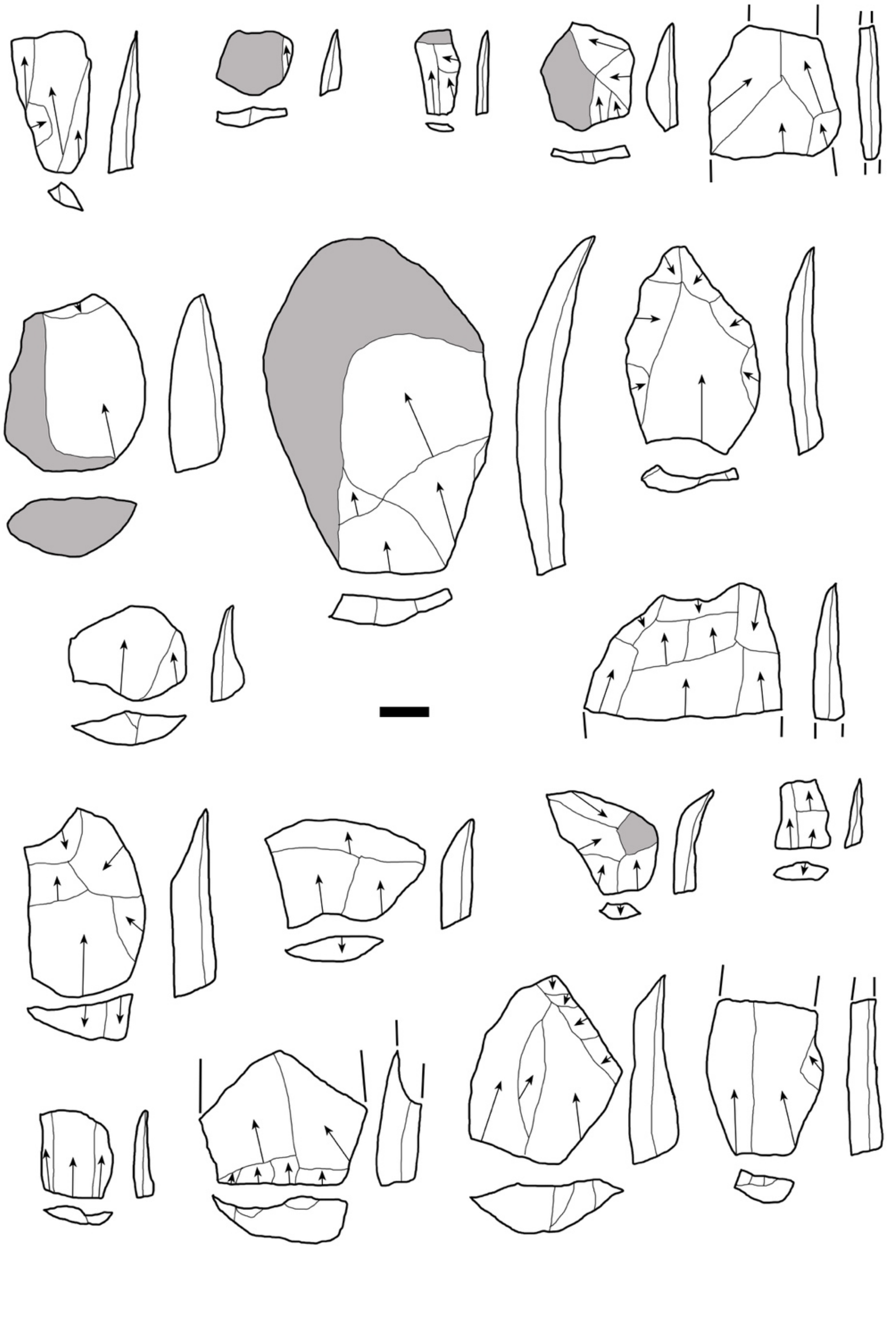

Supplementary figure 35. MIS 7 flakes from JQ-1. Scale: 1cm, grey: cortex.

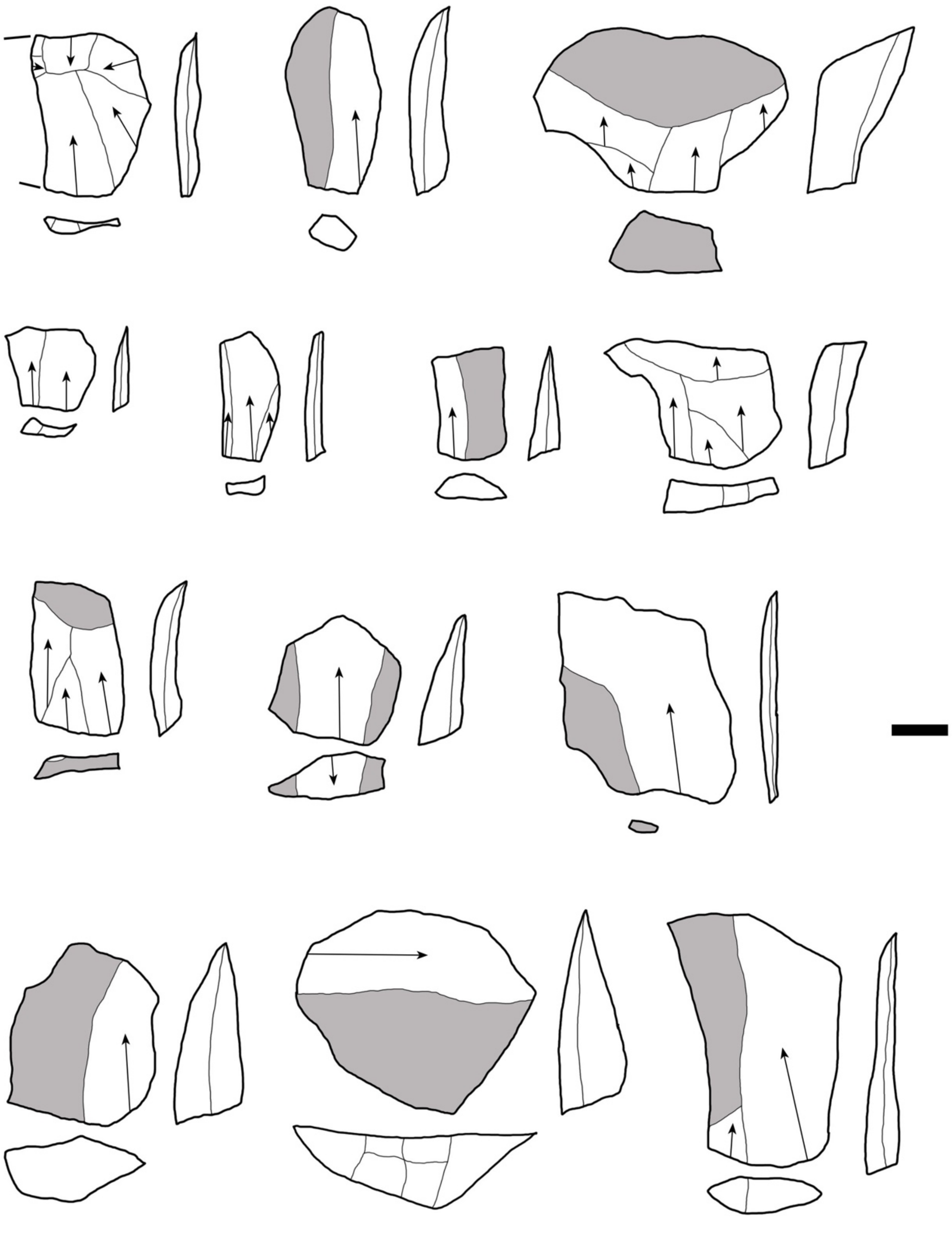

Supplementary figure 36. JSM-1 flakes. An example of a collection of flakes from a single sedimentary context, unit 3C of trench 3 at JSM-1. Except bottom row, which are other example of preparation flakes from across the site. Scale: 1cm, grey: cortex.

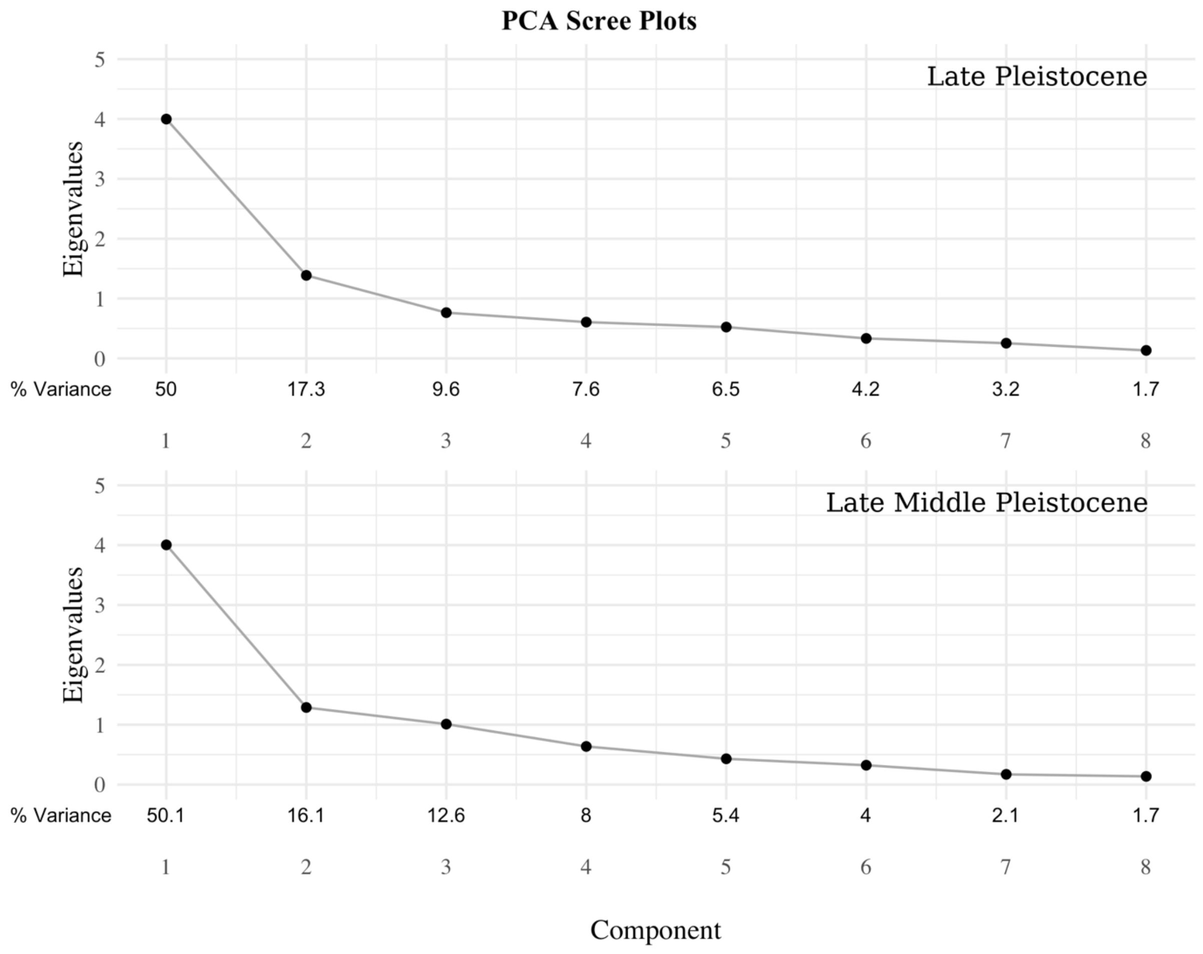

Supplementary figure 37. Scree plots for the two PCA analyses.

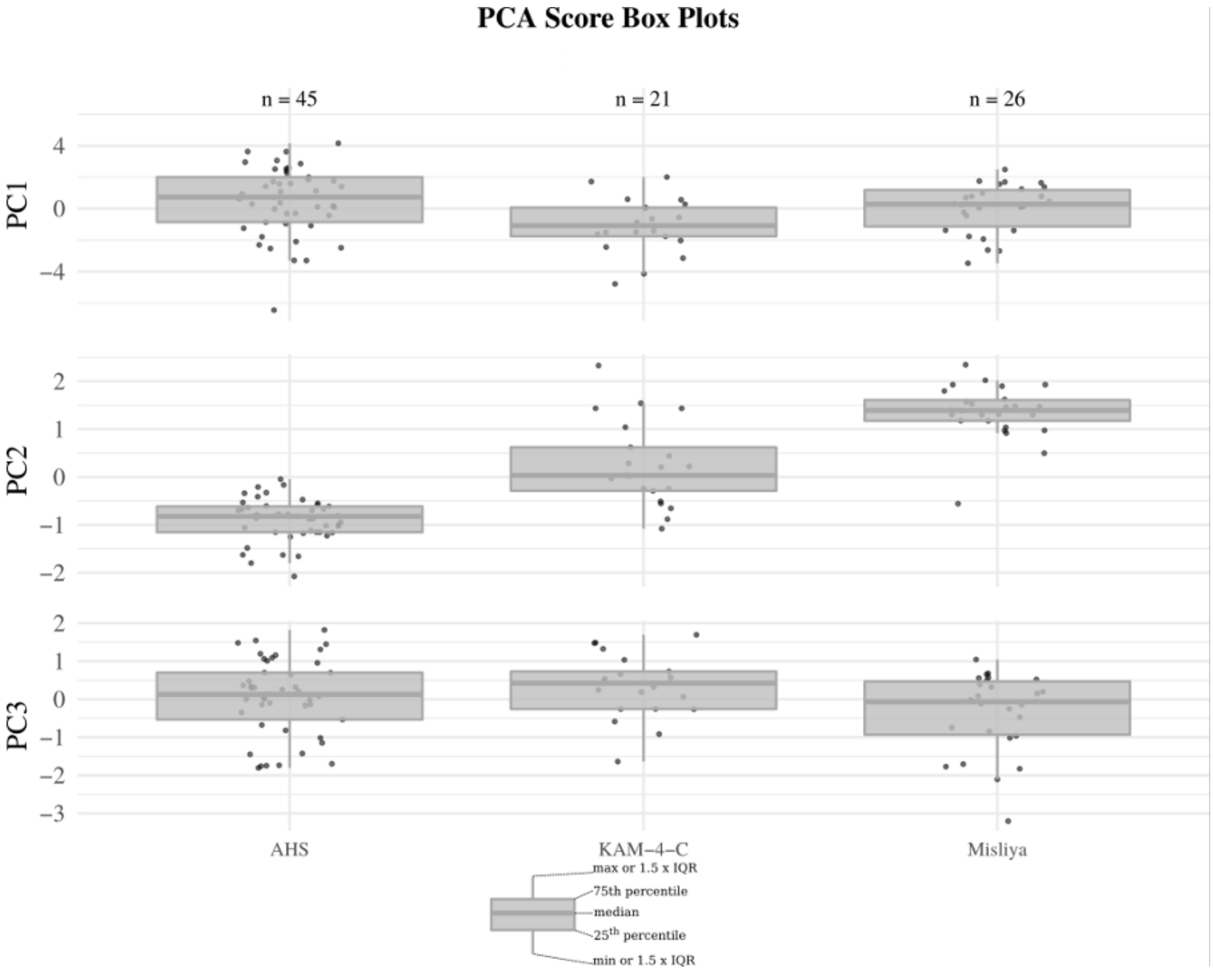

Supplementary figure 38. Score plots for late Middle Pleistocene (MIS 7/6) PCA. Note that the horizontal positions of the points have been randomly jittered to improve visibility. Sample sizes for each assemblage are noted at the top of the plot.

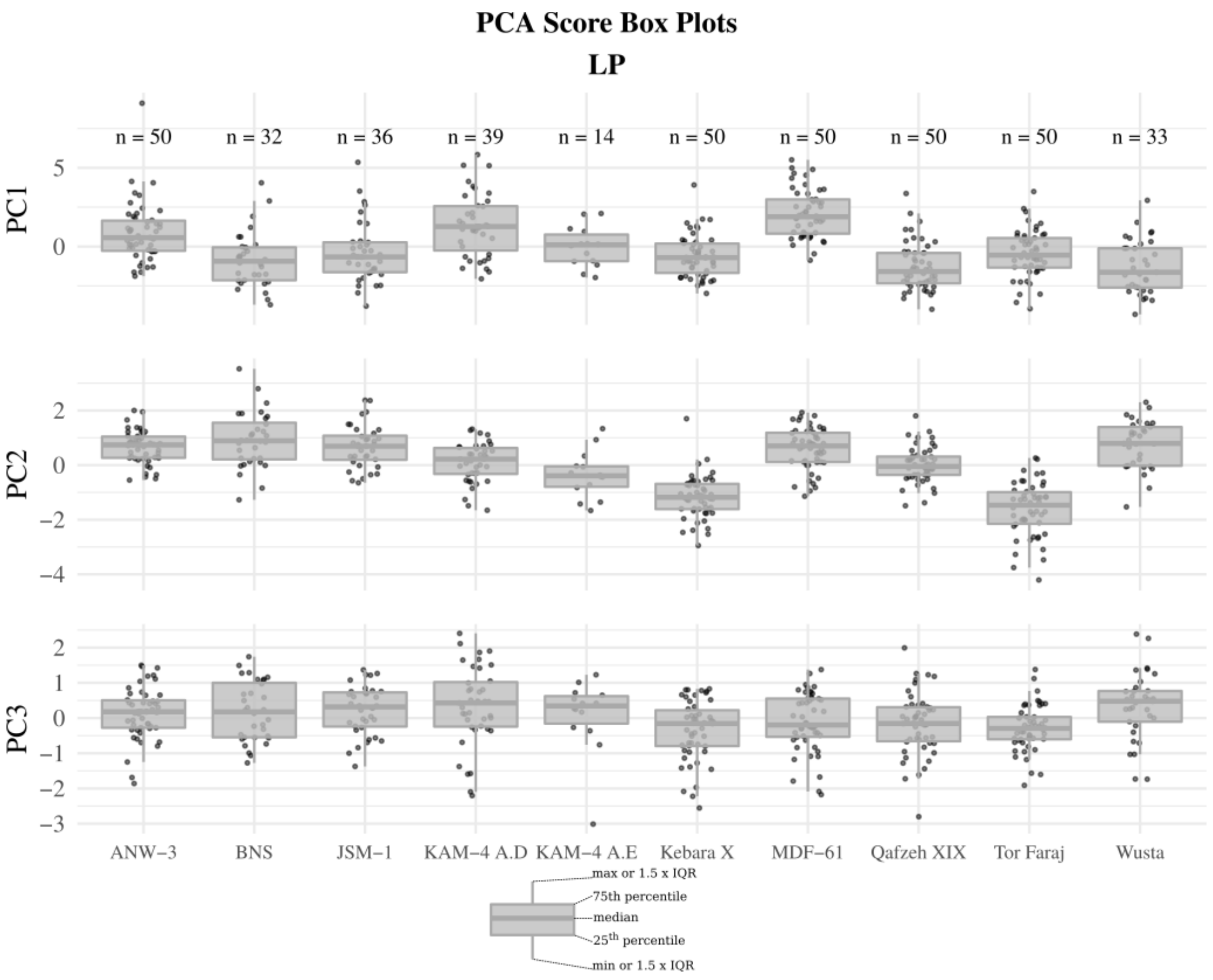

Supplementary figure 39. Score plots for Late Pleistocene PCA. Note that the horizontal positions of the points have been randomly jittered to improve visibility. Sample sizes for each assemblage are noted at the top of the plot.

Supplementary references

39. Jelinek, A.J. The Tabun Cave and Paleolithic man in the Levant. *Science* **216**, 1369-1375 (1982).
40. Shea, J.J. Transitions or turnovers? Climatically-forced extinctions of *Homo sapiens* and Neanderthals in the east Mediterranean Levant. *Quatern. Sci. Rev.* **27**, 2253-2270 (2008).
41. Roberts, P., Stewart, B.A. Defining the 'generalist specialist' niche for Pleistocene *Homo sapiens*. *Nat. Hum. Behav.* **2**, 542-550 (2018).
42. Mellars, P., Gori, K.C., Carr, M., Soares, P.A., Richards, M.B. Genetic and archaeological perspectives on the initial modern human colonization of southern Asia. *Proc. Natl. Acad. Sci. USA* **110**, 10699-10704 (2013).
43. Mirazón Lahr, M., Foley, R.A. Towards a theory of modern human origins: Geography, demography, and diversity in recent human evolution. *Yearbook Phys. Anth.* **41**, 137-176 (1998).
44. Adler, D.S, et al. Early Levallois technology and the Lower to Middle Paleolithic transition in the southern Caucasus. *Science* **345**, 1609-1613 (2014).
45. Bergström, A., et al. Insights into human genetic variation and population history from 929 diverse genomes. *Science* **367**, eaay5012 (2020).
46. Stutz, A.J. *et al.* Early Upper Paleolithic chronology in the Levant: New ABOx-SC accelerator mass spectrometry results from the Mughr el-Hamamah site, Jordan. *J. Hum. Evol.* **85**, 157-173 (2015).
47. Hublin, J.J. The modern human colonization of western Eurasia: when and where? *Quatern. Sci. Rev.* **188**, 194-210 (2015).
48. Hershkovitz, I., *et al*, The earliest modern humans outside Africa. *Science* **359**, 456-459 (2018).
49. Sharp, W.D., Paces, J.B. Comment on "the earliest modern humans outside Africa". *Science* **362**, eaat6598 (2018).
50. Shea, J.J. *Stone Tools in the Paleolithic and Neolithic of the Near East: A Guide* (Cambridge University Press, 2012).
51. Ekshtain, R., *et al,* Persistent Neanderthal occupation of the open-air site of 'Ein Qashish, Israel. *PLoS ONE* **14**, e0215668 (2019).
52. Malinky-Buller, A. The Muddle in the Middle Pleistocene: The Lower-Middle Paleolithic transition from the Levantine perspective. *J. World Prehist.* **29**, 1-78 (2016).
53. Goren-Inbar, N. Culture and cognition in the Acheulian industry: a case study from Gesher Benot Ya'aqov. *Phil. Trans. Royal. Proc. B.* **366**, 1038-1049 (2011).
54. Bar-Yosef, O. The lower paleolithic of the Near East. *J. World Prehist.* **8**, 211-265 (1994).
55. Shimelmitz, R., Weinstein-Evron, M., Ronen, A., Kuhn, S.L. The Lower to Middle Paleolithic transition and the diversification of Levallois technology in the Southern Levant: Evidence from Tabun Cave, Israel. *J. Hum. Evol.* **409**, 23-40 (2016).
56. Stewart, M., et al. Human footprints provide snapshot of late interglacial ecology in the Arabian interior. *Science Advances* **6,** eaba8940 (2020).
57. Zaidner, Y., Weinstein-Evron, M., The end of the Lower Paleolithic in the Levant: The Acheulo-Yabrudian lithic technology at Misliya Cave, Israel. *Quatern. Int.* **409**, 9-22 (2016).
58. Groucutt, H.S., *et al*, Human occupation of the Arabian Empty Quarter during MIS 5: Evidence from Mundafan Al-Buhayrah, Saudi Arabia. *Quatern. Sci. Rev.* **119**, 116-135 (2015).

59. Crassard, R., Hilbert, Y.H., Preusser, F. Wulf, G., Schiettecatte, J. Middle Palaeolithic occupations in central Saudi Arabia during MIS 5 and MIS 7: New insights on the origins of the peopling of Arabia. *Arch. Anth. Sci.* **11**, 3101-3120 (2019).
60. Stimpson, C.M., et al. Middle Pleistocene vertebrate fossils from the Nefud Desert, Saudi Arabia: implications for biogeography and palaeoecology. *Quatern. Sci. Rev.* **143**, 13–36. (2016).
61. Roberts, P., et al. Fossil herbivore stable isotopes reveal middle Pleistocene hominin palaeoenvironment in 'Green Arabia'. *Nature Eco. Evo.* **2**, 1871-1878 (2018).
62. Stewart, M., et al. Taphonomic and zooarchaeological investigations at the middle Pleistocene site of Ti's al Ghadah, western Nefud Desert, Saudi Arabia. *Quatern. Sci. Rev.* **218**, 228–253 (2019).
63. Whitney, J.W., Faulkender, D.J., Meyer, R. The environmental history and present condition of Saudi Arabia's northern sand seas. USGS open-file report **83**, 749-788 (1983).
64. Rosenberg, T.M., et al, 2011. Humid periods in southern Arabia: Windows of opportunity for modern human dispersal: Geology **39**, 1115–1118 (2011)
65. Edgell, H.S. *Arabian Deserts: Nature, origin and evolution* (Springer, Netherlands, 2006).
66. Groucutt, H.S., et al. Middle Palaeolithic raw material procurement and early stage reduction at Jubbah, Saudi Arabia. *Arch. Res. Asia.* **9**, 44-62 (2017).
67. Cohen, A.S. *Paleolimnology: the history and evolution of lake systems* (Oxford University Press, 2003).
68. Petraglia M.D., *et al.* Middle Paleolithic occupation on a Marine Isotope Stage 5 lakeshore in the Nefud Desert, Saudi Arabia. *Quatern. Sci. Rev.* **30**, 1555-1559 (2011).
69. Scerri, E.M.L., Groucutt, H.S., Jennings, R.P., Petraglia, M.D. Unexpected technological heterogeneity in northern Arabia indicates complex Late Pleistocene demography at the gateway to Asia. *J. Hum. Evol.* **75**, 125-142 (2014).
70. Duller, G.A.T. *Luminescence dating: guidelines in using luminescence dating in archaeology. (*English Heritage, Swindon, 2008).
71. Wintle, A.G. Luminescence dating: where it has been and where it is going. *Boreas* **37**, 369-370 (2008).
72. Rhodes, E. J. Optically stimulated luminescence dating of sediments over the past 200,000 years. *Annual Rev. Earth Plan. Sci.*, **39**, 461–488 (2011).
73. Aitken, M.J. *An Introduction to Optical Dating: the Dating of Quaternary Sediments by the Use of Photon-stimulated Luminescence*. (Oxford Science Publications, Oxford, 1998).
74. Galbraith, R., Roberts, R.G., Laslette, G., Yoshidha Olley, J., 1999. Optical dating of single and multiple grain quartz from Jinmium Rock Shelter, Northern Australia. Part I, experimental design and statistical models. *Archaeometry* **41**, 339–364 (1999).
75. Wintle, A., Murray, A.S. A review of quartz optically stimulated luminescence characteristics and their relevance in single-aliquot regeneration dating protocols. *Radiat. Meas.* **41**, 369-391 (2006).
76. Thomsen, K.J., Murray, A.S., Jain, M., Bøtter-Jensen, L. Laboratory fading rates of various luminescence signals from feldspar-rich sediment extracts. *Radiat. Meas.* **43**, 1474-1486. (2008).
77. Huntley, D. J., Lamothe, M. Ubiquity of anomalous fading in K-feldspars and the measurement and correction for it in optical dating. *Can. J. Earth Sci.* **38**, 1093–1106 (2001).
78. Wintle, A.G. Anomalous fading of thermo-luminescence in mineral samples. *Nature* **245**, 143–144 (1973).
79. Lamothe, M., Barré, M., Huot, S., Ouimet, S. Natural luminescence and anomalous fading in K-feldspar. *Radiat. Meas.* **47**, 682–687 (2012).
80. Aitken, M.J. *Thermoluminescence Dating* (Academic Press, London, 1985).

81. Bøtter-Jensen, L., Andersen, C.E., Duller, G.A.T., Murray, A.S., Developments in radiation, stimulation and observation facilities in luminescence measurements. *Radiat. Meas.* **37**, 535–541 (2003).
82. Duller, G.A.T., Bøtter-Jensen, L., Murray, A.S., Truscott, A.J. Single grain laser luminescence (SGLL) measurements using a novel automated reader. *Nuclear Instruments and Methods in Physics B* **155**, 506-514 (1999).
83. Armitage, S.J., Bailey, R.M. The measured dependence of laboratory beta dose rates on sample grain size. *Radiat. Meas.* **39**, 123-127 (2005).
84. Ballarini, M., Wintle, A.G., Wallinga, J. Spatial variation of dose rate from beta sources as measured using single grains. *Ancient TL* **24**, 1-7 (2006).
85. Murray, A.S., Wintle, A.G. Luminescence dating of quartz using an improved single-aliquot regenerative-dose protocol. *Radiat. Meas.* **32**, 57–73 (2000).
86. Murray, A.S., Wintle, A.G. The single aliquot regenerative dose protocol: potential for improvements in reliability. *Radiat. Meas.* **37**, 377–381 (2003).
87. Duller, G.A.T. Distinguishing quartz and feldspar in single grain luminescence measurements. *Radiat. Meas.* **37**, 161–165 (2003).
88. Duller, G.A.T. Assessing the error on equivalent dose estimates derived from single aliquot regenerative dose measurements. *Ancient TL* **25**, 15-24 (2007).
89. Thiel, C., et al. Luminescence dating of the Stratzing loess profile (Austria) – Testing the potential of an elevated temperature post-IR IRSL protocol. *Quatern. Int.* **234**, 23–31 (2011).
90. Kars, R.H., Busschers, F.S., Wallinga, J. Validating post IR-IRSL dating on K-feldspars through comparison with quartz OSL ages. *Quaernt. Geochronol.* **12**, 74–86 (2012).
91. Li, B., Jacobs, Z., Roberts, R.G., Li, S-H. Review and assessment of the potential of post-IR IRSL dating methods to circumvent the problem of anomalous fading in feldspar luminescence. *Geochronometria* **41**, 178–201 (2014).
92. Buylaert, J.P., et al. Luminescence dating of the PASADO core 5022-1D from Laguna Potrok Aike (Argentina) using IRSL signals from feldspar. *Quatern. Sci. Rev.* **71**, 70-80 (2013).
93. Auclair, M., Lamothe, M., Huot, S. Measurement of anomalous fading for feldspar IRSL using SAR. *Radiat. Meas.* **37**, 487–492 (2003).
94. Buylaert, J.P., Jain, M., Murray, A.S., Thomsen, K.J., Thiel, C., Sohbati, R. A robust feldspar luminescence dating method for Middle and Late Pleistocene sediments. *Boreas* **41**, 435–451 (2012).
95. Roberts, H.M. Testing Post-lR IRSL protocols for minimising fading in feldspars, using Alaskan loess with independent chronological control. *Radiat. Meas.* **47,** 716-724. (2012).
96. Lowick, S.E., Trauerstein, M., Preusser, F. Testing the application of post IR- IRSL dating to fine grain waterlain sediments. *Quatern. Geochronol.* **8**, 33-40 (2012).
97. Roberts, R.G., Galbraith, R.F., Yoshida, H., Laslett, G.M., Olley, J.M. Distinguishing dose populations in sediment mixtures: a test of optical dating procedures using mixtures of laboratory-dosed quartz. *Radiat. Meas.* **32**, 459–465 (2000).
98. Galbraith, R.F., Laslett, G.M. Statistical models for mixed fission track ages. *Nucl. Tracks Radiat. Meas.* **21**, 459–470 (1993).
99. Galbraith, R.F., Green, P.F. Estimating the component ages in a finite mixture. *Int. J. Radiat. Appl. Instrum. D. Nucl. Tracks Radiat. Meas.* 17, 197–206 (1990).
100. Bailey, R.M., Arnold, L.J. Statistical modelling of single grain quartz De distributions and an assessment of procedures for estimating burial dose. *Quatern. Sci. Rev.* **25,** 2475–2502 (2006).
101. Galbraith, R.F., Roberts, R.G. Statistical aspects of equivalent dose and error calculation and display in OSL dating: an overview and some recommendations. *Quatern. Geochron.* **11**, 1–27 (2012).

102. Bøtter-Jensen, L., Mejdahl, V. Assessment of beta dose-rate using a GM multicounter system. *Nucl. Tracks Radiat. Meas.* **10**, 663-666 (1988).
103. De Corte, F., Vandenberghe, D., Hossain, S.M., De Wispelaere, A., Buylaert, J.-P., Van Den Haute, P. Preparation and characterization of loess sediment for use as a reference material in the annual radiation dose determination for luminescence dating. *J. Radioanal. Nucl. Chem.* **272**, 311–319 (2007).
104. Adamiec, G., Aitken, M.J., 1998. Dose-rate conversion factors: update. *Anc. TL* **16**, 37-46 (1998).
105. Brennan, B.J., Lyons, R.G., Phillips, S.W. Attenuation of alpha particle track dose for spherical grains. *Int. J. Radiat. Appl. Instrum. Part D. Nucl. Tracks Radiat. Meas.* **18**, 249-253 (1991).
106. Mercier, N., Falguères, C. Field gamma dose-rate measurement with a NaI (Tl) detector: re-evaluation of the “threshold” technique. *Anc. TL* **25**, 1-4 (2007).
107. Duval, M., Arnold, L.J. Field gamma dose-rate assessment in natural sedimentary contexts using LaBr3(Ce) and NaI(Tl) probes: a comparison between the “threshold” and “windows” techniques. *Appl. Radiat. Isot.* **74**, 36-45 (2013).
108. Huntley, D.J., Baril, M.R. The K content of the K-feldspars being measured in optical dating or in thermoluminescence dating. *Ancient TL* **15**, 11–13 (1997).
109. Huntley, D.J., Hancock, R.G.V. The Rb contents of the K-feldspar grains being measured in optical dating. *Ancient TL* **19**, 43–46 (2001).
110. Brennan, B.J. Beta doses to spherical grains. *Radiat. Meas.* **37**, 299-303 (2003).
111. Readhead, M.L. Absorbed dose fraction for 87Rb beta particles. *Anc. TL* **20**, 25-29 (2002).
112. Balescu, S., Lamothe, M. Comparison of TL and IRSL age estimates of feldspar coarse grains from waterlain sediments. *Quatern. Sci. Rev.* **13**, 437-444 (1994).
113. Rees-Jones, J. Optical dating of young sediments using fine-grained quartz. *Anc. TL* **13**, 9-14 (1995).
114. Bell, W.T. Attenuation factors for the absorbed radiation dose in quartz inclusions for thermoluminescence dating. *Anc. TL* **8**, 1-12. (1979).
115. Prescott, J.R., Hutton, J.T. Cosmic ray contributions to dose rates for luminescence and ESR dating: large depths and long-term variations. *Radiat. Meas.* **23**, 497-500 (1994).
116. Munyikwa, K. Cosmic ray contribution to environmental dose rates with varying overburden thickness. *Ancient TL* **18**, 27-34 (2000).
117. Diaz, N., Armitage, S.J., Verrechia, E.P., Herman, F. OSL dating of a carbonate island in the Chobe Enclave, NW Botswana. Quarter. Geochron. **49**, 172-176 (2019).
118. Janjou, D., Halawani, M., Roobol, M.J., Memesh, A.M., Razin, P., Shorbaji, H., Roger, J. Explanatory Notes to the Geological Map of the Jibal Al Misma Quadrangle, Sheet 27 D. Kingdom of Saudi Arabia, Jiddah (1998).
119. Schulz, E., Whitney, J.W. Upper pleistocene and holocene lakes in the An Nafud, Saudi Arabia. Hydrobiologia 143, 175-190.9 (1986)
120. Mayya, Y.S., Morthekai, P., Murari Madhav, K., Singhvi, A.K. Towards quantifying beta microdosimetric effects in single-grain quartz dose distribution, *Radiat. Meas.* **41**, 1032-1039 (2006).
121. Armitage, S.J., Krishna, A., Parker, L.E., King, G.E. Optically stimulated luminescence dating of heat retainer hearths from the Sahara: Insights into signal accumulation and measurement. *Quatern. Geochron.* **49**, 249-253 (2019).
122. Grün, R. Direct dating of human fossils. *Am. J. Phys. Anth.* **131**, 2–48 (2006).
123. Pike, A.W.G., Hedges, R.E.M. U-series dating of bone using the diffusion-adsorption model. *Geochim. et Cosmochim. Acta*, **66**, 4273–4286 (2002).

124. Grün, R., McDermott, F. Open system modelling for U-series and ESR dating of teeth. *Quatern. Sci. Rev.* **13**, 121–125 (1994).
125. Eggins, S.M, et al. In situ U-series dating by laser-ablation multi-collector ICPMS: new prospects for Quaternary geochronology. *Quatern. Sci. Rev.* **24**, 2523–2538 (2005).
126. Sambridge, M., Grün, R. and Eggins, S. U-series dating of bone in an open system: the diffusion-adsorption-decay model. *Quatern. Geochron.* **9**, 42–53 (2012).
127. Shao, Q., Bahain, J.J., Falguères, C., Dolo, J.M. and Garcia, T. A new U-uptake model for combined ESR/U-series dating of tooth enamel. *Quatern. Geochron.* **10**, 406–411 (2012).
128. Price, G.J., Feng, Y.X., Zhao, J.X., Webb, G.E. Direct U–Th dating of vertebrate fossils with minimum sampling destruction and application to museum specimens. *Quatern. Geochron.* **18**, 1–8 (2013).
129. Zhou, H., Zhao, J., Qing, W., Feng, Y., Tang, J. Speleothem-derived Asian summer monsoon variations in Central China, 54–46 ka. *J. Quatern. Sci.* **26**, 781-790 (2011).
130. Grün, R. The relevance of parametric U-uptake models in ESR age calculations. *Radiat. Meas.* **44**, 472–476 (2009).
131. Grün, R., Schwarcz, H.P. and Chadam, J. ESR dating of tooth enamel: Coupled correction for U-uptake and U-series disequilibrium. *Int. J. Radiat. App Instr. D. Nuclear Tracks Radi. Meas.* **14**, 237-241 (1988).
132. Duval, M., Falguères, C., Bahain, J.J. Age of the oldest hominin settlements in Spain: contribution of the combined U-series/ESR dating method applied to fossil teeth. *Quatern. Geochron.*, **10**, 412–417 (2012).
133. Petraglia, M.D., Shipton, C. Large cutting tool variation west and east of the Movius Line. *J. Hum. Evol.* **55**, 962-6 (2008).
134. Shipton, C., Petraglia, M.D. Inter-continental variation in Acheulean bifaces. In *Asian Paleoanthropology* (eds Norton, C., Braun, D.) 49-55 (Springer, Dordrecht, 2010).
135. Copeland, L. The Late Acheulean knapping floor at C-Spring, Azraq Oasis, Jordan. *Levant* **23**, 1-6 (1991).
136. Zaidner, Y., Druc, D., Weinstein, Evron, M. Acheulo-Yabrudian handaxes from Misliya Cave, Mount Carmel, Israel. In *Axe Age: Acheulian tool-making from quarry to discard* (ed. Goren-Inbar, N., Sharon, G.) 243-266 (Equinox, London, 2006).
137. Bar-Yosef, O. Archaeological occurrences in the Middle Pleistocene of Israel. In *After the Australopithecines (*ed. Butzer, K.W., Isaac, G.L) 571-604 (Mouton, The Hague, 1975).
138. McPherron, S.P. What typology can tell us about Acheulian handaxe production. In *Axe Age: Acheulian tool-making from quarry to discard* (ed. Goren-Inbar, N., Sharon, G.) 267-286 (Equinox, London, 2006).
139. Gisis, I. Bifaces from the Acheulian and Yabrudian layers of Tabun Cave, Israel. In *Axe Age: Acheulian tool-making from quarry to discard* (ed. Goren-Inbar, N., Sharon, G.) 137-154 (Equinox, London, 2006).
140. Copeland, L. Analysis of the Palaeolithic artefacts from the sounding of A. Garrard at C-Spring, 1985 season. In *The Hammer on The Rock: Studies in the Early Palaeolithic of Azraq, Jordan* (*part 2)* (eds L. Copeland, F. Hours) 329-290 (BAR International 540, Oxford, 1989).
141. Copeland, L. The artifacts from the sounding of D. Kirkbride at Lion Spring, Azraq in 1956. In *The Hammer on The Rock: Studies in the Early Palaeolithic of Azraq, Jordan* (*part 2)* (eds L. Copeland, F. Hours) 171-212 (BAR International 540, Oxford, 1989).
142. Goren-Inbar, N. The lithic assemblage of the Berekhat Ram Acheulian site, Golan Heights. *Paléorient* **11**, 7-28 (1985).
143. Chazan, M., Horowitz (eds.) *Holon: A Lower Palaeolithic site in Israel*. American School of Prehistoric Research Bulletin 50. (Peabody Museum, Cambridge, 2007).

144. Marder, O., Milevski, I., Matskevich, Z. The handaxes of Revedim Quarry: Typo-technological considerations and aspects of intra site variability. In *Axe Age: Acheulian tool-making from quarry to discard* (ed. Goren-Inbar, N., Sharon, G.) 223-242 (Equinox, London, 2006).
145. Goren-Inbar, N, Saragusti, L. An Acheulian biface assemblage from the site of Gesher Benot Ya'Aqov, Israel: Indications of African affinities. *J. Field Arch.* **23**, 15-30 (1996).
146. Bar-Yosef, O., Goren-Inbar, N. *The Lithic Assemblages of Ubeidiya: A Lower Palaeolithic site in the Jordan Valley*. (Hebrew University, Jerusalem, 1993).
147. Gilead, D. 1977. Some metrical studies of Acheulian assemblages in Israel. *Eretz Israel*, *13*, pp.38-49.
148. Rose, J.I., *et al*, The Nubian Complex of Dhofar, Oman: A Middle Stone Age industry in southern Arabia. *PLoS ONE* **6**, e28239 (2011).
149. Shipton, C., et al. 78,000-year-old record of Middle and Later Stone Age innovation in an East African tropical forest. *Nat. Coms.* **9**, 1832 (2018).
150. Yellen, J. The archaeology of Aduma Middle Stone Age sites in the Awash Valley, Ethiopia. *Paleoanth.* **2005**, 25-100 (2005).
151. Jolliffe, I.T. *Principal Component Analysis* (Springer, New York, 1986).
152. Bartlett, M.S.. The effect of standardization on a chi-square approximation in factor analysis, *Biometrika* **38**, 337-344 (1951).
153. Kaiser, H.F. An index of factor simplicity, *Psychometrika* 39, 31-36 (1974).
154. Revelle, W. psych: procedures for personality and psychological research, https://CRAN.R-project.org/package=psych Version = 1.9.12. (2019).
155. R Core Team, R: A Language and Environment for Statistical Computing. https://www.r-project.org. (2020).
156. McClure, H.A. *Late Quaternary Palaeoenvironments of the Rub' al Khali*. Ph.D. Thesis, (University College, London, 1984).
157. Thomas, H., et al. First Pleistocene faunas from the Arabian Peninsula: an Nafud Desert, Saudi Arabia. *Comptes Rendus de l'Académie des Sciences, Series IIA, Earth and Planetary Science* **326**, 145–152 (1998).
158. Eltringham, S.K. *The Hippos: Natural History and Conservation*. (Academic Press, London, 1999).
159. Horwitz, L.K., Tchernov, E. Cultural and environmental implications of hippopotamus bone remains in archaeological contexts in the Levant. *Bull. Am. Sch. Orient. Res.* **280**, 67–76 (1990).
160. Pokines, J.T., Lister, A.M., Ames, C.J.H., Nowell, A., Cordova, C.E. Faunal remains from recent excavations at Shishan Marsh 1 (SM1), a late Lower Paleolithic open-air site in the Azraq Basin, Jordan. *Quatern. Res.* **91**, 768–791 (2019).
161. Ben-Dor, M., Gopher, A., Hershkovitz, I., Barkai, R. Man the fat hunter: the demise of *Homo erectus* and the emergence of a new hominin lineage in the middle Pleistocene (ca. 400 kyr) Levant. *PLoS ONE* **6**(12), e28689 (2011).
162. Guagnin, M., et al. Rock art provides new evidence on the biogeography of kudu (*Tragelaphus imberbis*), wild dromedary, aurochs (*Bos primigenius*) and African wild ass (*Equus africanus*) in the early and middle Holocene of north-western Arabia. *J. Biogeog.* **45**, 727-740 (2018).
163. Jennings, RP., et al. The greening of Arabia: Multiple opportunities for human occupation of the Arabian Peninsula during the Late Pleistocene inferred from an ensemble of climate model simulations. *Quatern. Int.* **382**, 181-199 (2015).
164. Parton, A., et al. Orbital-scale climate variability in Arabia as a potential motor for human dispersals. *Quatern. Int.* **382**, 82-97 (2015).

165. Past Interglacials working group of PAGES. Interglacials of the last 800,000 years. *Review of Geophysics* **54**, 162-219 (2016).
166. Hoffman, G., Rupprechter, M., Rahn, M., Preusser, F. Fluvio-lacustrine deposits reveal precipitation pattern in SE Arabia during early MIS 3. *Quatern. Int.* **382**, 145-153 (2015).
167. Parton, A., et al. Alluvial fan records from southeast Arabia reveal multiple windows for human dispersal. *Geology* **43**, 295-298 (2015).
168. Directional changes in Levallois core technologies between Eastern Africa, Arabia, and the Levant during MIS 5. *Scientific Reports* **11**, 11465 (2021).
169. Posth, C., et al. Deeply divergent archaic mitochondrial genome provides lower time boundary for African gene flow into Neanderthals. *Nat. Com.* **8**, 16046.
170. Akhilesh, K., Pappu, S., Rajapara, H. M., Gunnell, Y., Shukla, A. D., Singhvi, A. K. Early Middle Palaeolithic culture in India around 385–172 ka reframes Out of Africa models. *Nature* **554**, 97-101 (2018).
171. Liu, W., et al. The earliest unequivocally modern humans in southern China. *Nature* **526**, 690-700 (2015).
172. Westaway, K.E., et al. An early modern human presence in Sumatra 73,000-63,000 years ago. *Nature* **548**, 322-325 (2017).
173. Petraglia, M.D. et al. Middle Paleolithic assemblages from the Indian Subcontinent before and after the Toba super-eruption. *Science* **317**, 114-116 (2007).
174. Blinkhorn, J., et al. Middle Palaeolithic occupation in the Thar Desert during the Upper Pleistocene: the signature of a modern human exit out of Africa. *Quatern. Sci. Rev.* **77**, 233-238 (2013).
175. Boivin, N., et al. Human dispersal across diverse environments of Asia during the Upper Pleistocene. *Quatern. Int.* **300**, 32-47 (2013).
176. Groucutt, H.S., et al. Stone tool assemblages and models for the dispersal of *Homo sapiens* out of Africa. *Quatern. Int.* 382, 8-30 (2015).
177. Clarkson, C., et al. Human occupation of northern India spans the Toba super-eruption ~74,000 years ago. *Nat. Comm.* **11**, 961 (2020).
178. Scerri, E.M.L. Did our species evolve in subdivided populations across Africa, and why does it matter? *Trends Eco. Evo.* **33**, 582-594 (2018).
179. Nicholson, S.L., et al. Beyond arrows on a map: The dynamics of *Homo sapiens* dispersal and occupation of Arabia during Marine Isotope Stage 5. *J. Anth. Arch.* **62**, 101269 (2021).
180. Welker, F., et al. The dental proteome of *Homo antecessor* **580**, 235-238 (2020).
181. Bretzke, K., Conard, N.J., Uerpmann, H.P. Excavations at Jebel Faya – the FAY-NE1 shelter sequence. *Proc. Sem. Arab. Stud.* **44**, 69-82 (2014).
182. Groucutt, H.S., Middle Palaeolithic point technology, with a focus on the site of Tor Faraj (Jordan, MIS 3). *Quatern. Int.* **350,** 205-226 (2014).
183. Rink, W.J., Richter, D., Schwarcz, H.P., Marks, A.E., Monigal, K., Kaufman, D. Age of the Middle Palaeolithic site of Rosh Ein Mor, Central Negev, Israel: Implications for the Age Range of the Early Levantine Mousterian of the Levantine Corridor. *J. Arch. Sci.* **30**, 195-204 (2003)
184. Goder-Goldberger, M., Bar-Matthews, M. Novel chrono-cultural constraints from the Middle Paleolithic site of Rosh Ein Mor (D15), Israel. *J. Arch Sci. Rep.* **24**, 102-114 (2019).
185. Clark, G.A., Schuldenrein, J., Donaldson, M.L., Schwarcz, H.P., Rink, W.J., Fish, S.K. Chronostratigraphic context of Middle Paleolithic horizons at the 'Ain Difla Rockshelter (WHS 634), West-Central Jordan. In *The Prehistory of Jordan II, Perspectives from 1997* (ed. Gebel, H.G.K., Kafafi, Z., Rollefson, G.) 77-100 (Ex Oriente, Berlin, 1997).

186. Goder-Goldberger, M., et al. The Middle to Upper Paleolithic transition in the southern Levant: New insights from the late Middle Paleolithic site of Far'ah II, Israel. *Quatern. Sci. Rev.* **237**, 106304 (2020).
187. Porat, N., Chazan, M., Schwarcz, H., Horwitz, L.K. Timing of the Lower to Middle Palaeolithic boundary: New dates from the Levant. *J. Hum. Evol.* **43**, 107-122 (2002).
188. Marder, O., Malinsky-Buller, A., Shahack-Gross, R., Ackermann, O., Ayalon, A., Bar-Matthews, M., et al. Archaeological horizons and fluvial processes at the Lower Paleolithic open-air site of Revadim (Israel). *J. Hum. Evol.* **60**, 508-522 (2011).
189. Malinsky-Buller, A., Hovers, E., Marder, O. Making time: 'Living floors', 'palimpsests' and site formation processes – A perspective from the open-air Lower Paleolithic site of Revadim Quarry, Israel. *J. Anth. Arch.* **30**, 89-101 (2011).
190. Zaidner, Y., Frumkin, A., Porat, N., Tsatskin, A., Yeshurun, R., Weissbrod, L. A series of Mousterian occupations in a new type of site: The Nesher Ramla karst depression, Israel. *J. Hum. Evol.* **66**, 1-17 (2014).
191. Nowell, A., et al. Middle Pleistocene subsistence in the Azraq Oasis, Jordan: Protein residue and other proxies. *J. Arch. Sci* **73**, 36-44 (2016).
192. Falguères, C., et al. New ESR-U-series dates in Yabrudian and Amudian layers at Qesem Cave, Israel. *Quatern. Int.* **398**, 6-12 (2016).
193. Porat, N., Zhou, L.P., Chazan, M., Noy, T., Horwitz, L.K. Dating the Lower Paleolithic Open-Air site of Holon, Israel by luminescence and ESR techniques. *Quatern.Res.* **51**, 328-341.
194. Goder-Goldberger, M.,et al. Emanuel Cave: The site and its bearing on Early Middle Paleolithic technological variability. *Paléorient* **38**, 203-225 (2012).
195. Grün, R., et al. U-Series and ESR analyses of bones and teeth relating to the human burials from Skhul. *J. Hum. Evol.* **49**, 316-34 (2005).
196. Groucutt, H.S., Scerri, E.M.L., Stringer, C., Petraglia, M.D. Skhul lithic technology and the dispersal of *Homo sapiens* into Southwest Asia. *Quatern. Int.* **515**, 30-52 (2019).
197. Mercier, N., Valladas, H. Reassessment of TL age estimates of burnt flints from the Paleolithic site of Tabun Cave, Israel. *J. Hum. Evol.* **45**, 401-409 (2003).
198. Shimelmitz, R., Weinstein-Evron, M., Ronen, A., Kuhn, S.L. The Lower to Middle Paleolithic transition and the diversification of Levallois technology in the southern Levant: Evidence from Tabun Cave, Israel. *Quatern. Int.* **409**, 23-40 (2016).
199. Valladas, H, et al. Dating the Lower to Middle Paleolithic transition in the Levant: A view from Misliya Cave, Mount Carmel, Israel. *J. Hum. Evol.* **65**, 585-593 (2013).
200. Valladas, H., Mercier, M., Joron, J.-L., Reyss, J.L. GIF laboratory dates for Middle Paleolithic Levant. In: *Neanderthals and Modern Humans in Western Asia* (eds. Akazawa, T., Aoki, K., Bar-Yosef, O.) 69-76 (Plenum, New York,1998).
201. Rink, W.J., Schwarcz, H.P., Lee, H.K., Rees-Jones J., Rabinovich, R., Hovers, E. Electron spin resonance (ESR) and thermal ionization mass spectrometric (TIMS) ^{230}Th/^{234}U dating of teeth in Middle Paleolithic layers at Amud Cave, Israel. *Geoarchaeology* **16**, 701-717 (2001).
202. Hovers, E. The lithic assemblages of Amud Cave: Implications for the end of the Mousterian in the Levant. In *Neanderthals and Modern Humans in the Levant* (eds. Akazawa, T., Aoki, K., Bar-Yosef, O. (Eds.) 143-163 (Plenum, New York, 1998).
203. Friesem, D.E., Malinsky-Buller, A., Ekshtain, R. Gur-Arieh, S., Vaks, A., Mercier, N. New data from Shovakh Cave and its implications for reconstructing Middle Paleolithic settlement patterns in the Amud drainage, Israel. *J. Palaeolithic Arch.* **2**, 298-237 (2019).
204. Meignen, L. Hayonim Cave lithic assemblages in the context of the Near Eastern Middle Paleolithic. In *Neanderthals and Modern Humans in the Levant* (eds. Akazawa, T., Aoki, K., Bar-Yosef, O. (Eds.) 165-180 (Plenum, New York, 1998).

205. Mercier, N., Valladas, H., Froget, L., Joron, J.L., Reyss, J-L., Weiner, S., et al. Hayonim Cave: a TL-based chronology for this Levantine Mousterian sequence. *J. Arch.Sci.* **34**, 1064-1077 (2007).
206. Kalbe, J., Sharon, G., Porat, N., Zhang, C., Mischke, S. Geological setting and age of the Middle Paleolithic site of Nahal Mahanayeem Outlet (Upper Jordan Valley, Israel). *Quatern. Int.* **331**, 139-148 (2014).
207. Sharon, G., Oron, M. The lithic tool arsenal of a Mousterian hunter. *Quatern. Int.* **331**, 167-185 (2014).
208. Hershkovitz, I. *et al.* Levantine cranium from Manot Cave (Israel) foreshadows the first European modern humans. *Nature* **520**, 216-219 (2015).
209. Goren-Inbar, N. *Quneitra: A Mousterian site on the Golan Heights* (Jerusalem, Hebrew University, 1990).
210. Fleisch, H. Les habitats du paléolithique moyen à Naamé (Liban). *Bulletin de la Musée de Beyrouth* **23**, 25-93 (1970).
211. Leroi-Gourhan, A. Les analyses polliniques au Moyen-Orient. *Paléorient* **6**, 79-91 (1980).
212. Marks, A.E., Volkman, P. The Mousterian of Ksar Akil: Levels XXVIA through XXVIIIB. *Paléorient* **12**, 5-20 (1986).
213. Bosch, M.D., Mannino, M.A., Prendergast, A.L., O'Connell, T.C., Demarchi, B., Taylor, S.M., et al. New chronology for Ksâr Akil (Lebanon) supports Levantine route of modern human dispersal into Europe. *PNAS* **112**, 7683-7688 (2015).
214. Copeland, L., Moloney, N. (eds.). *The Mousterian Site of Ras el-Kelb*, *Lebanon*. British Archaeological Reports, BAR International Series **706**. (BAR, Oxford) (1998).
215. Porat, N., Schwarcz, H.P. Use of signal subtraction methods in ESR dating of burned flint. *Nucl. Tracks. Radiat. Meas.* **18**, 203-212 (1991).
216. Solcki, R.S. The Middle Palaeolithic site of Nahr Ibrahim (Asfourieh Cave) in Lebanon. In *Problems in Prehistory: North Africa and the Levant* (eds. Wendorf, F., Marks, A.) 286-296 (Dallas, SMU Press, 1975).
217. Kai, A., Miki, T., Ikeya, M. ESR ages of Paleolithic site of Douara Cave, Syria. *Journal of Speleological Society of Japan* **12**, 41-49 (1987).
218. Boëda, E., et al. Middle Palaeolithic bitumen use at Umm el Tlel around 70 000 BP. *Antiquity* **82**, 853-861 (2008).
219. Griggo, C., Boëda, E., Bonilaurie, S., Al Sakhle, H, Emery-Barbier, A., Courty, M.A. A Mousterian dromedary hunting camp: Level VIIa0 at Umm el Tlel (El Kowm, Central Syria). *Palethnology* **3**, 103-129 (2011).
220. Hauck, T., Wojtczak, D., Wegmüller, F., Le Tensorer, J.M. Variation in Lower and Middle Paleolithic land use strategies in the Syrian Desert Steppe: The example of Hummal (El Kowm area). In *Settlement Dynamics of the Middle Paleolithic and Middle Stone Age, Vol. III* (eds. Conard, N., Delagnes, A.) 145-162 (Kerns Verlag, Tübingen, 2010).
221. Hauck, T. Mousterian technology and settlement dynamics in the site of Hummal (Syria). *J. Hum. Evol.* **61**, 519-537 (2011).
222. Jagher, R. Nadaouiyeh Aïn Askfar, an example of Upper Acheulean variability in the Levant. *Quatern. Int.* **411**, 44-58 (2016).
223. Nishiaki, Y., Kanjo, Y., Muhesen, S., Akazawa, T. The temporal variability of late Levantine Mousterian lithic assemblages from Dederiyeh Cave, Syria. *Eurasian Prehistory* **9**, 3-27 (2012).
224. Pomeroy, E. et al. New Neanderthal remains associated with the 'flower burial' at Shanidar Cave. *Antiquity* **94**, 11-256 (2020).

225. Hilbert, Y., et al. Epipalaeolithic occupation and palaeoenvironments of the southern Nefud desert, Saudi Arabia, during the Terminal Pleistocene and Early Holocene. *J. Arch. Sci.* **50**, 460-474 (2014).
226. Ludwig, K. R., 2003. *Users Manual for Isoplot/Ex version 3.0: A Geochronological Toolkit for Microsoft Excel*. Berkeley: Berkeley Geochronology Centre.
227. Ludwig, K.R., Simmons, K.R., Szabo, B.J., Winograd, I.J., Landwehr, J.M., Riggs, A.C. and Hoffman, R.J., Mass-spectrometric ^{230}Th-^{234}U-^{238}U dating of the Devils Hole calcite vein. *Science*, **258**, 284–287 (1992).
228. Cheng, H., Edwards, R.L., Hoff, J., Gallup, C.D., Richards, D.A., & Asmerom, Y. The half-lives of uranium-234 and thorium-230. Chem. *Geology* **169**, 17–33 (2000).
