# Supplementary material for: Multiple hominin dispersals into Southwest Asia over the past 400,000 years
Source: Nature. 2021 Sep 1;597(7876):376–80. doi: 10.1038/s41586-021-03863-y (PMC8443443; doi:10.1038/s41586-021-03863-y)
Supplement: Supplementary file 2 — Reporting Summary [file 41586_2021_3863_MOESM2_ESM.pdf]

nature portfolio

Corresponding author(s): Huw Groucutt, Michael Petraglia

Last updated by author(s): Jul 19, 2021

Reporting Summary

Nature Portfolio wishes to improve the reproducibility of the work that we publish. This form provides structure for consistency and transparency in reporting. For further information on Nature Portfolio policies, see our Editorial Policies and the Editorial Policy Checklist.

Statistics

For all statistical analyses, confirm that the following items are present in the figure legend, table legend, main text, or Methods section.

n/a	Confirmed	
☐	☒	The exact sample size (*n*) for each experimental group/condition, given as a discrete number and unit of measurement
☐	☒	A statement on whether measurements were taken from distinct samples or whether the same sample was measured repeatedly
☐	☒	The statistical test(s) used AND whether they are one- or two-sided<br>*Only common tests should be described solely by name; describe more complex techniques in the Methods section.*
☒	☐	A description of all covariates tested
☐	☒	A description of any assumptions or corrections, such as tests of normality and adjustment for multiple comparisons
☐	☒	A full description of the statistical parameters including central tendency (e.g. means) or other basic estimates (e.g. regression coefficient) AND variation (e.g. standard deviation) or associated estimates of uncertainty (e.g. confidence intervals)
☐	☒	For null hypothesis testing, the test statistic (e.g. *F*, *t*, *r*) with confidence intervals, effect sizes, degrees of freedom and *P* value noted<br>*Give P values as exact values whenever suitable.*
☒	☐	For Bayesian analysis, information on the choice of priors and Markov chain Monte Carlo settings
☒	☐	For hierarchical and complex designs, identification of the appropriate level for tests and full reporting of outcomes
☐	☒	Estimates of effect sizes (e.g. Cohen's *d*, Pearson's *r*), indicating how they were calculated

Our web collection on statistics for biologists contains articles on many of the points above.

Software and code

Policy information about availability of computer code

Data collection	For collection of the luminescence data, software specific to the instruments (Risø TL-DA-15 and TL-DA-20) was used.
Data analysis	Luminescence data were analysed using Analyst v.4.31.9 (individual equivalent doses and fading rates) and R v. 3.6.1 using v.0.9.10 of the Luminescence package (age models, fast ratios, fading corrections, cosmic dose rates, Abanico plots). The PCA was conducted using R 4.1.0, psych 2.1.3, and ggplot2 package (3.3.3).

For manuscripts utilizing custom algorithms or software that are central to the research but not yet described in published literature, software must be made available to editors and reviewers. We strongly encourage code deposition in a community repository (e.g. GitHub). See the Nature Portfolio guidelines for submitting code & software for further information.

Data

Policy information about availability of data

All manuscripts must include a data availability statement. This statement should provide the following information, where applicable:

- Accession codes, unique identifiers, or web links for publicly available datasets
- A description of any restrictions on data availability
- For clinical datasets or third party data, please ensure that the statement adheres to our policy

All relevant data is included in the paper and SI, or for the PCA analysis the data and code is archived at DOI: 10.5281/zenodo.5082293.

Field-specific reporting

Please select the one below that is the best fit for your research. If you are not sure, read the appropriate sections before making your selection.

☐ Life sciences ☐ Behavioural & social sciences ☒ Ecological, evolutionary & environmental sciences

For a reference copy of the document with all sections, see nature.com/documents/nr-reporting-summary-flat.pdf

Ecological, evolutionary & environmental sciences study design

All studies must disclose on these points even when the disclosure is negative.

Study description	Stone tools and samples for chronometric dating were collected from archaeological sites in the western Nefud Desert of Saudi Arabia. Stone tools were described both qualitatively and with descriptive statistics. For the comparison of Levallois flakes, the structure of the dataset was explored using PCA analysis of standard metric measurements, with comparative material selected from neighbouring regions. No experimental factors or control groups were used in such analyses, where PCA was conducted on the dataset and then data were plotted by assemblage name. Luminescence dating was conducted to determine the ages of the assemblages.
Research sample	The locations of study sites were determined by a combination of remote sensing and field prospecting. Systematic pedestrian transects were conducted across the sites and all objects of interest (lithics, fossils, etc.) were recorded using a differential GPS system or total station. Excavations following standard archaeological practices were conducted in areas of particular interest (i.e. with high artefact densities). Sections of trenches were samples for luminescence dating and palaeoenvironmental analysis. For the PCA analysis comparing Levallois flake morphologies, a series of assemblages from surrounding regions were sampled.
Sampling strategy	For the comparative lithic aspect, for large assemblages, Levallois flakes were sampled to give sample sizes of several dozen. Sufficient sample sizes were judged as being larger than the number of pieces included in complete assemblages.
Data collection	The main forms of data collected for this interdisciplinary study consist of 1) lithic artefacts, which were studied by Huw Groucutt, Eleanor Scerri, and James Blinkhorn. Standard metrics and technological classifications were recorded using goniometers and entered into excel files, 2) Luminescence dating was carried out by Eric Andrieux, Simon Armitage and Richard Clark-Wilson, with input from Laine Clark-Balzan; Gamma dose rates for samples prefixed "PD" were measured using an EG&G Ortec digiDart-LF instrument while for those prefixed "JSM" or "KAM4-OSL", an Inspector 1000 was used. Luminescence measurements were performed using Risø TL/OSL-DA-15 or Risø TL/OSL-DA-20 instruments. Beta dose rates for "JSM" and "PD" samples were measured using a Risø GM-25-5 low-level beta counting system, 3) U-Series dating was conducted by Gilbert Price and Mathieu Duval. Drilling was conducted at Griffith University, and U-Series dating at the University of Queensland
Timing and spatial scale	Fieldwork and initial data collected during field seasons in Saudi Arabia in 2013 to 2015. All analyses were conducted in an intermittent manner from 2013 onwards and were completed in 2020.
Data exclusions	All data collected were analysed and contributed to the final conclusions
Reproducibility	The archaeological analyses are descriptive, and reproducibility is possible by re-analysis of the data. The luminescence component of this study involved measurements of multiple individual quartz and K-feldspar grains, or multi-grain aliquots of the same, yielding distributions of equivalent-dose estimates from which the weighted mean was calculated using well-established statistical models (see SI).
Randomization	The only aspect this applies to is a few cases where Levallois flakes were sampled from large assemblages. A random sample of flakes laid out on a table was taken. These data were used for descriptive statistics, categorised by the relevant assemblage.
Blinding	No blinding was conducted as we are reporting descriptive statistics, not experimental results.

Did the study involve field work? ☒ Yes ☐ No

Field work, collection and transport

Field conditions	Fieldwork was conducted in warm (typically in the 20s C) and dry conditions in Saudi Arabia in two main fields seasons.
Location	The sites are located in the western Nefud Desert, in northern Saudi Arabia. Supplementary table S1 gives more information.
Access & import/export	Permission to conduct the research and relevant export permits were provided by the Saudi Ministry of Culture. Multiple permits were provided to M.D. Petraglia from 2010 onwards.
Disturbance	Excavations were backfilled

Reporting for specific materials, systems and methods

We require information from authors about some types of materials, experimental systems and methods used in many studies. Here, indicate whether each material, system or method listed is relevant to your study. If you are not sure if a list item applies to your research, read the appropriate section before selecting a response.

Materials & experimental systems

n/a	Involved in the study
☒	☐ Antibodies
☒	☐ Eukaryotic cell lines
☐	☒ Palaeontology and archaeology
☒	☐ Animals and other organisms
☒	☐ Human research participants
☒	☐ Clinical data
☒	☐ Dual use research of concern

Methods

n/a	Involved in the study
☒	☐ ChIP-seq
☒	☐ Flow cytometry
☒	☐ MRI-based neuroimaging

Palaeontology and Archaeology

Specimen provenance	Fieldwork was conducted in the western Nefud, with permits for research and export issued to M.D. Petraglia from 2010 onwards.
Specimen deposition	Archaeological samples are stored at the National Museum, Riyadh, Saudi Arabia.
Dating methods	New age estimates were generated by luminescence dating and U-series dating, as discussed above.

☒ Tick this box to confirm that the raw and calibrated dates are available in the paper or in Supplementary Information.

Ethics oversight	All fieldwork was permitted and overseen by the Saudi Ministry of Culture.

Note that full information on the approval of the study protocol must also be provided in the manuscript.
