# Supplementary material for: Author Correction: Multiple hominin dispersals into Southwest Asia over the past 400,000 years
Source: Nature. 2022 Jan 10;602(7895):E15. doi: 10.1038/s41586-021-04289-2 (PMC8810378; doi:10.1038/s41586-021-04289-2)
Supplement: Supplementary file 1 — This file contains a revised analysis with detailed description of the error and links to corrected and uncorrected data, scripts and replication instructions. [file 41586_2021_4289_MOESM1_ESM.pdf]

nature portfolio

https://doi.org/10.1038/s41586-021-04289-2

Supplementary information

Author Correction: Multiple hominin dispersals into Southwest Asia over the past 400,000 years

In the format provided by the authors and unedited

The original published version of this article contains an error in the Late Pleistocene (LP) lithic data and PCA reported in the Supplementary Information associated with the original published version of this article. During preparation of the LP lithic dataset, the flaking length measurement was accidently inserted into the original data file twice for 89 of 404 cases, being pasted over the midpoint width values which should have been in the second column in these cases.

We corrected the error and re-ran the PCA. The analyses were conducted in R and the relevant scripts, corrected and uncorrected data, and full replication instructions for the corrected analysis have been archived with Zenodo (https://zenodo.org/record/5797578#.YcLZliwo9hA) and have been assigned a permanent DOI (10.5281/zenodo.5797578).

The results of our re-analysis indicated that the duplicated measurements had no significant effect on our original results. This was because they constituted only 89 out of 3232 measurements (404 cases with 8 variables) used in our PCA and the variables concerned are highly correlated. Ultimately, the results are very similar to the original ones, and there is no impact on the interpretation from these data. Table 1 below shows the relevant cases with the duplicated and corrected variables shaded in dark gray and lighter gray respectively.

Table 1: Late Palaeolithic artifact measurements showing duplicated and corrected cells. The original (duplicated) "Width-at-Midpoint" measurements are shaded in darker gray and the corrected measurements in lighter grey. The "Flaking-Length" column from which the measurements were accidentally duplicated is also shaded in dark gray.

**Assemblage**	**ID**	**N.scars**	**Flaking.Length**	**Width.at.Midpoint**	**Width.at.Midpoint**	**Proximal.Width**	**Distal.Width**	**Thickness.at.midpoint**	**Platform.Width**	**Platform.Thickness**
ANW-3	1	7	48.22	48.22	34.56	24.56	24.13	9.26	20.48	6.98
ANW-3	2	8	45.69	45.69	26.62	25.77	6.32	6.17	25.69	7.54
ANW-3	3	8	44.15	44.15	26.97	41.89	20.59	12.74	39.68	13.11
ANW-3	4	7	51.55	51.55	27.48	48.08	32.03	11.17	47.61	11.54
ANW-3	5	8	59.69	59.69	36.88	34.62	27.49	13.7	31.32	13.29
ANW-3	6	8	46.1	46.1	23.52	42	30.58	7.85	38.65	7.55
ANW-3	7	7	45.6	45.6	43.16	26.58	12.5	7.88	23.28	8.5
ANW-3	9	8	31.54	31.54	37.61	26.83	32.34	9.71	25.4	9.73
ANW-3	10	5	33.7	33.7	43.87	26.94	17.44	7.86	22.08	7.31
ANW-3	11	12	75.06	75.06	42.02	60.34	40.33	17.76	59.99	20.18
ANW-3	35	9	46.1	46.1	32.15	29	32.34	6.76	28.81	9.21
ANW-3	52	5	46.32	46.32	32.43	24.9	28.26	8.59	23.47	9.64
ANW-3	41	10	32.42	32.42	40.61	29.55	18.96	6.62	26.15	7.14
ANW-3	60	11	50.25	50.25	37.89	36.95	20.88	8.6	31.11	9.89
ANW-3	46	10	31.99	31.99	32.52	23.85	23.31	6.06	18.48	5.44
ANW-3	100	4	38.29	38.29	57.11	33.16	21.7	9.31	34.68	10.83
ANW-3	6	10	64.07	64.07	28.02	37.18	27.01	7.61	34.08	9.78
ANW-3	27	7	40.11	40.11	27.72	25.2	17.82	8.59	24.23	8.44
ANW-3	13	7	40.36	40.36	20.59	20.89	12.76	6.69	18.87	7.55
ANW-3	40	9	58.58	58.58	30.56	26.41	22.27	6.06	21.03	6.7
ANW-3	39	6	51.17	51.17	32.15	36.78	34.77	9.03	33.35	11.22
ANW-3	47	6	35.25	35.25	32.33	27.79	9.08	5.7	29.67	7.07
ANW-3	100	7	46.9	46.9	48.5	28.45	24.67	8.61	25.71	7.73
ANW-3	12	5	35.62	35.62	29.38	28.09	12.93	8.3	24.15	8.26
ANW-3	57	6	37.03	37.03	28.53	28.76	26.24	6.28	19.16	8.24
ANW-3	65	11	34.46	34.46	17.16	28.27	12.77	5.37	27.04	6.59

ANW-3	19	9	26.26	26.26	35.16	20.03	7.51	6.07	19.38	6.7
ANW-3	61	10	50.41	50.41	25.33	17.75	7.26	6.74	17.23	5.17
ANW-3	59	5	41.44	41.44	30.26	23.55	24.75	8.04	19.78	6.17
ANW-3	57	9	43.09	43.09	31.89	30.27	24.16	9.6	33.86	8.95
ANW-3	41	7	48.96	48.96	29.88	37.01	24.19	7.66	36.1	9.25
ANW-3	56	8	41.86	41.86	32.24	34.18	23.19	8.68	35.64	8.51
ANW-3	59	6	41.99	41.99	26.38	34.84	27.95	10.5	35.65	8.13
ANW-3	61	9	33.35	33.35	28.56	22.93	25.56	8.21	15.95	5.42
ANW-3	41	6	48.25	48.25	24.65	22.06	21.96	8.4	20.19	10.36
ANW-3	9	8	48.37	48.37	32.4	25.5	22.79	6.51	22.37	9.98
ANW-3	2	6	32.49	32.49	39.16	25.67	17.34	4.97	21.84	4.75
ANW-3	5	6	45.79	45.79	25.11	32.21	23.33	6.06	22.22	5.84
ANW-3	43	4	35.8	35.8	35.6	30.71	22.15	7.03	30.72	9
ANW-3	43	8	29.56	29.56	42.11	20.97	20.91	5.78	22.94	6.51
ANW-3	100	7	34.18	34.18	37.28	15.09	9.8	4.58	16.26	5.72
ANW-3	40	7	22.58	22.58	40.88	24.94	20.68	8.44	28.84	8.06
ANW-3	4	8	33.78	33.78	33.26	26.12	12.53	9.6	26.42	8.21
ANW-3	41	5	29.69	29.69	28.9	22.9	20.25	3.83	20.39	6.62
ANW-3	65	7	28.29	28.29	31.14	18.13	10.59	4.23	17.75	5.25
ANW-3	36	6	30.13	30.13	20.65	20.4	18.19	5.08	19.06	6.39
ANW-3	47	7	30.21	30.21	25.24	26.75	21.86	9.37	26.61	9.94
ANW-3	1	8	53.33	53.33	19.05	26.75	18.38	6.63	29.97	8.93
ANW-3	41	8	37.45	37.45	38.51	17.32	13.99	2.66	17.19	4.15
ANW-3	56	5	38.2	38.2	32.25	35.95	15.87	11.69	36.59	11.9
KAM-4 A.D	175	6	39.99	39.99	41.69	41.69	38.2	11.1	37.92	13.78
KAM-4 A.D	176	3	47.63	47.63	28.47	28.47	18.72	7.95	22.7	5.13
KAM-4 A.D	195	6	76	76	47.32	47.32	21.2	10.7	28.32	6.7
KAM-4 A.D	199	4	32.93	32.93	28.12	28.12	14.53	6.04	19.88	5.56
KAM-4 A.D	203	6	34.05	34.05	33.34	33.34	29.42	8.02	23.18	7.34
KAM-4 A.D	204	6	48.97	48.97	40.44	40.44	26.24	9.94	21.43	7.47
KAM-4 A.D	210	6	43.78	43.78	26.38	26.38	20.06	9.53	19.38	8.32
KAM-4 A.D	215	5	41.71	41.71	38.52	38.52	31.93	9.36	15.58	6.24
KAM-4 A.D	216	4	48.06	48.06	39.58	39.58	30.15	9.89	30.31	8.53
KAM-4 A.D	221	6	65.42	65.42	32.32	32.32	17.17	7.86	15.49	4.69
KAM-4 A.D	233	6	75.4	75.4	35.01	35.01	13.64	10.46	32.18	10.92
KAM-4 A.D	241	5	70.09	70.09	58.36	58.36	39.77	13.88	22.98	10.71
KAM-4 A.D	245	5	52.37	52.37	41.71	41.71	12.22	9.69	48.12	7.47
KAM-4 A.D	260	3	32.92	32.92	20.99	20.99	7.85	5.27	22.31	9.26
KAM-4 A.D	262	4	38.74	38.74	28.93	28.93	21.44	7.28	12.25	19.13
KAM-4 A.D	268	8	59.84	59.84	46.88	46.88	35.35	10.45	17.04	6.13
KAM-4 A.D	270	5	53.47	53.47	55.94	55.94	33.43	11.73	30.09	12.92
KAM-4 A.D	287	5	44.55	44.55	46.22	46.22	44.64	10.34	33.12	6.69
KAM-4 A.D	296	3	35.71	35.71	29.04	29.04	20.39	11.97	28.24	11.44
KAM-4 A.D	315	5	48.95	48.95	41.9	41.9	28.51	8.9	15.09	6.96
KAM-4 A.D	342	4	34.53	34.53	22.6	22.6	13.67	4.64	13.44	4.85
KAM-4 A.D	33333	8	49.25	49.25	70.53	70.53	66.31	12.77	35.39	12.52
KAM-4 A.D	352	6	23.8	23.8	41.33	41.33	35.61	8.56	32.63	10.86
KAM-4 A.D	355	5	27.04	27.04	33.51	33.51	37.28	9.99	25.66	10.81
KAM-4 A.D	382	5	28.75	28.75	25.62	25.62	20.14	5.94	29.62	6.23
KAM-4 A.D	398	5	35.79	35.79	26.06	26.06	29.79	7.99	10.75	4.8

KAM-4 A.D	420	6	48.04	48.04	47.74	47.74	32.79	17.68	47.18	13.81
KAM-4 A.D	429	4	35.61	35.61	41.02	41.02	21.96	10.08	37.7	8.94
KAM-4 A.D	456	5	74.85	74.85	42.25	42.25	5.19	8.72	30.81	10.94
KAM-4 A.D	466	7	33.28	33.28	31.89	31.89	21.19	9.53	10.68	6.61
KAM-4 A.D	481	5	23.25	23.25	24.18	24.18	17.74	7.21	25.48	7.1
KAM-4 A.D	631	6	42.84	42.84	38.68	38.68	34.67	11.38	33.6	5.88
KAM-4 A.D	773	4	47.42	47.42	33.14	33.14	13.95	7.14	37.71	7.96
KAM-4 A.D	798	5	29.84	29.84	18.43	18.43	18.11	6.05	16.89	5.7
KAM-4 A.D	802	5	21.54	21.54	22.05	22.05	21.09	5.64	14.31	3.95
KAM-4 A.D	819	5	38.59	38.59	22.89	22.89	10.15	5.82	19.06	6.82
KAM-4 A.D	824	5	29.5	29.5	20.01	20.01	7.53	7.15	15.62	6.77
KAM-4 A.D	890	8	42.52	42.52	35.34	35.34	22.95	7.74	26.85	6.26
KAM-4 A.D	913	5	52.11	52.11	28.86	28.86	18.63	6.52	32.52	7.26

Despite the copying error having no qualitative effect on our interpretations or analyses, one table and two figures need to be updated to reflect minor quantitative changes in our results. These updates pertain only to the original published Supplementary Information and are as follows.

Table 26 was originally as it appears below (Table 2). It has now been updated to the subsequent Table 3. The revised loading scores are very similar, with no changes that would lead to reinterpretations of the meaning of the main principal components.

Table 2. Original PCA loading table.

	**PC1**	**PC2**	**PC3**	**PC4**	**PC5**	**PC6**	**PC7**	**PC8**
**N. Scars**	0.1590	0.6196	-0.2902	0.6746	-0.2215	0.0223	-0.0226	-0.0379
**Flaking Length**	0.3030	-0.2823	-0.7650	-0.0812	0.1986	-0.1234	-0.4186	0.0824
**Width at Midpoint**	0.4275	0.0910	-0.1993	-0.2527	-0.0800	-0.3469	0.7503	0.1251
**Proximal Width**	0.4209	-0.2980	0.0743	0.0289	-0.3964	0.1646	-0.0175	-0.7370
**Distal Width**	0.3027	0.4733	0.2014	-0.5330	-0.3343	-0.1027	-0.4639	0.1488
**Thickness at Midpoint**	0.4062	0.1593	0.0494	-0.0954	0.4295	0.7678	0.1133	0.1066
**Platform Width**	0.3526	-0.4331	0.2746	0.3538	-0.3326	0.0421	-0.0737	0.6080
**Platform Thickness**	0.3769	0.0484	0.4086	0.2350	0.5841	-0.4848	-0.1661	-0.1724

Table 3. Updated PCA loading table.

	PC1	PC2	PC3	PC4	PC5	PC6	PC7	PC8
**N. Scars**	0.1491	0.5867	-0.3603	0.6788	-0.2014	0.0053	-0.0439	0.0179
**Flaking Length**	0.2992	-0.2717	-0.7709	-0.1468	0.2567	-0.2800	0.2640	0.0822
**Width at Midpoint**	0.4414	0.1014	-0.0861	-0.3346	-0.1657	-0.0474	-0.7812	0.1888
**Proximal Width**	0.4165	-0.3300	0.0243	0.1160	-0.3760	0.1073	0.0342	-0.7413
**Distal Width**	0.3047	0.4909	0.1903	-0.4537	-0.3267	-0.2496	0.5035	0.0406
**Thickness at Midpoint**	0.4017	0.1772	0.0351	-0.0891	0.4400	0.7620	0.1515	0.0281
**Platform Width**	0.3606	-0.4323	0.2434	0.3305	-0.2985	0.0496	0.1899	0.6221
**Platform Thickness**	0.3685	0.0587	0.4137	0.2630	0.5790	-0.5123	-0.0668	-0.1353

Supplementary Figure 37 was originally as presented in Figure 1 below. This figure contains Scree plots that indicate the importance of each principal component in terms of the variance each explains. The top panel refers to the Late Pleistocene lithic data that contained the copying error. Compare this plot with the updated version presented in Figure 2. Again, the differences are very minor---only tenths of a percent in variance explained by the first three components.

Figure 1. Original PCA Scree plots.

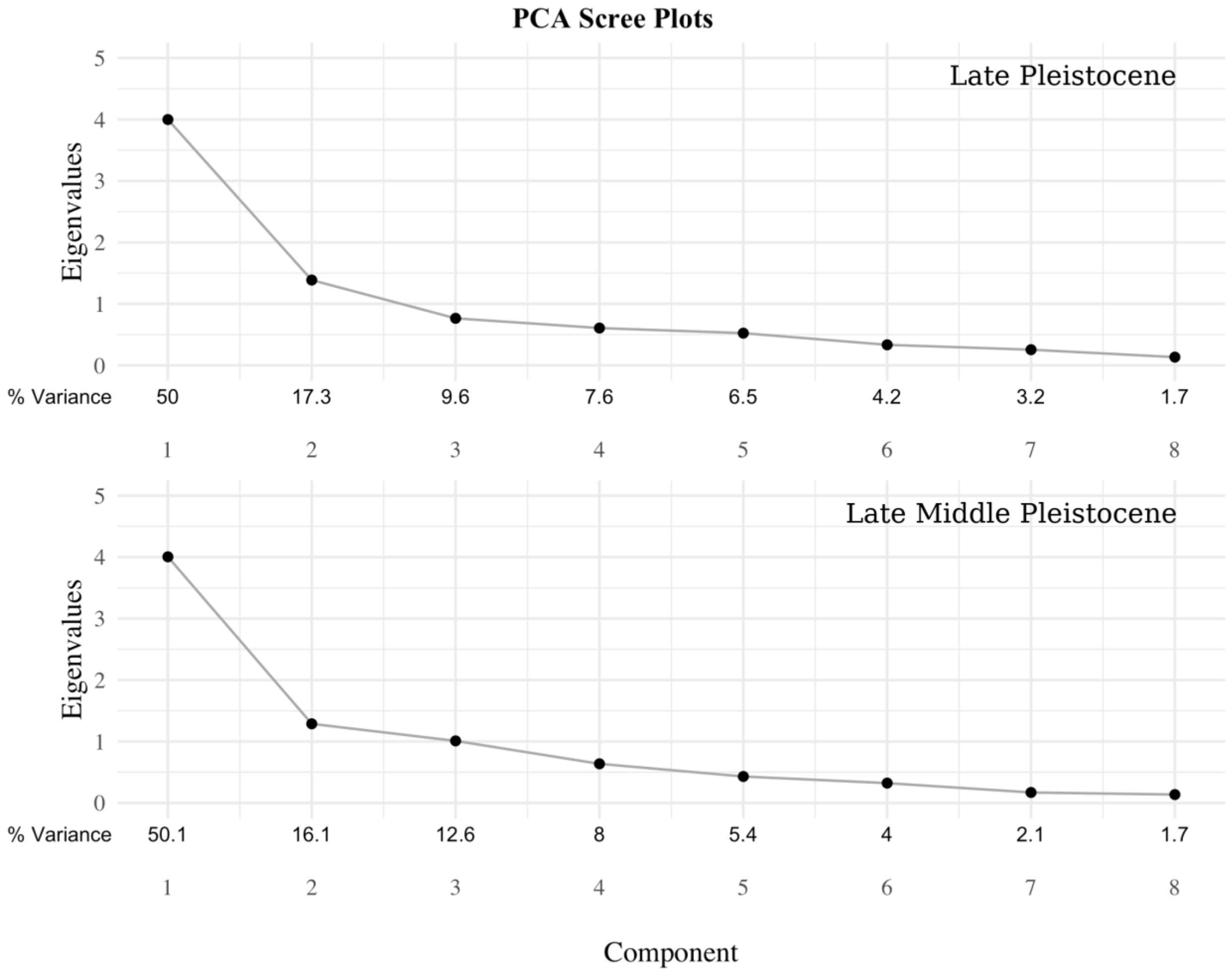

Figure 2. Revised PCA Scree Plot.

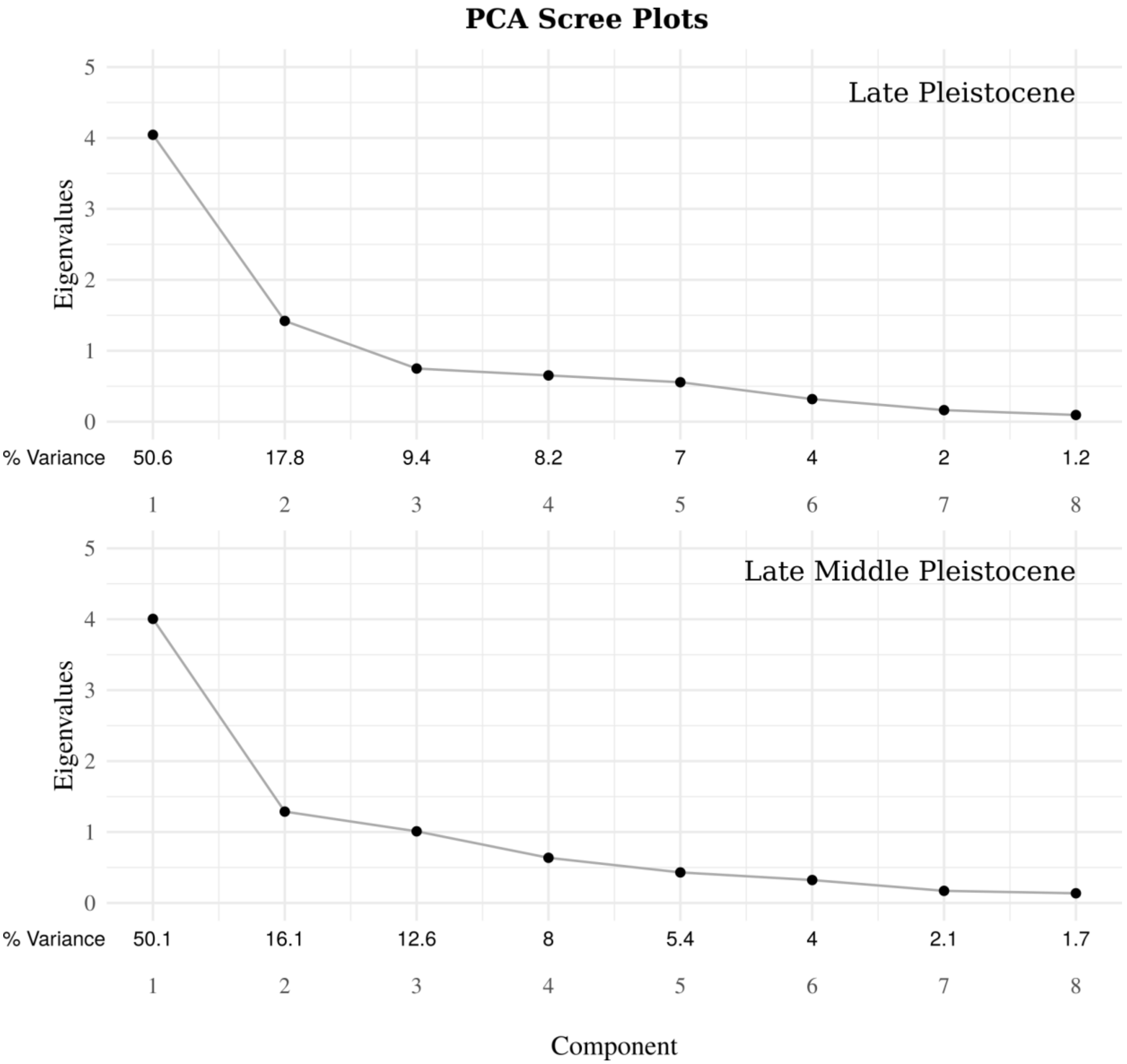

Lastly, Supplementary Figure 39 was also updated. The original appears below in Figure 3 followed by the revised version in Figure 4. The plots each contain three panels showing the projected coordinates for the lithic samples from a series of assemblages. The points each refer to the value of a given lithic observation (one tool) along a given principal component axis and the box plots summarize the distributions. Differences between the original and revised plots are very minor, but some small differences are visible. Look for example at the box plots for the assemblage from KAM-4 A.D. The distribution of that assemblage with respect to the first principal component is shifted down slightly, though the median remains above zero. The median with respect to the second principal component is shifted up slightly in the revised plot compared to the original. These differences are slight and the relative positions of the various

assemblages remained largely unchanged. Correcting the copying error, therefore, has had no significant effect on the interpretations and conclusions of the original published study.

Figure 3. Original PCA Score Boxplots.

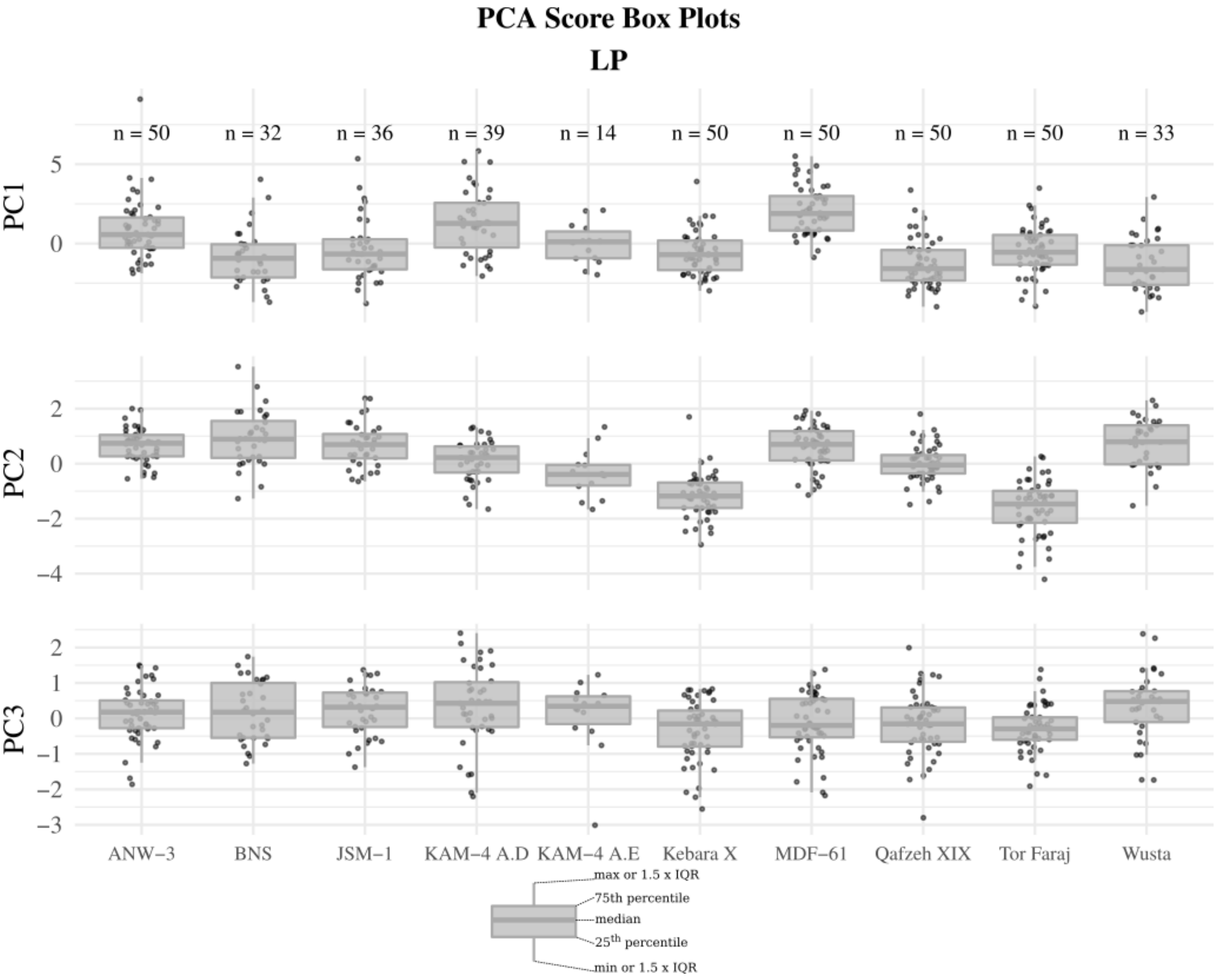

Figure 3. Revised PCA Score Boxplots.

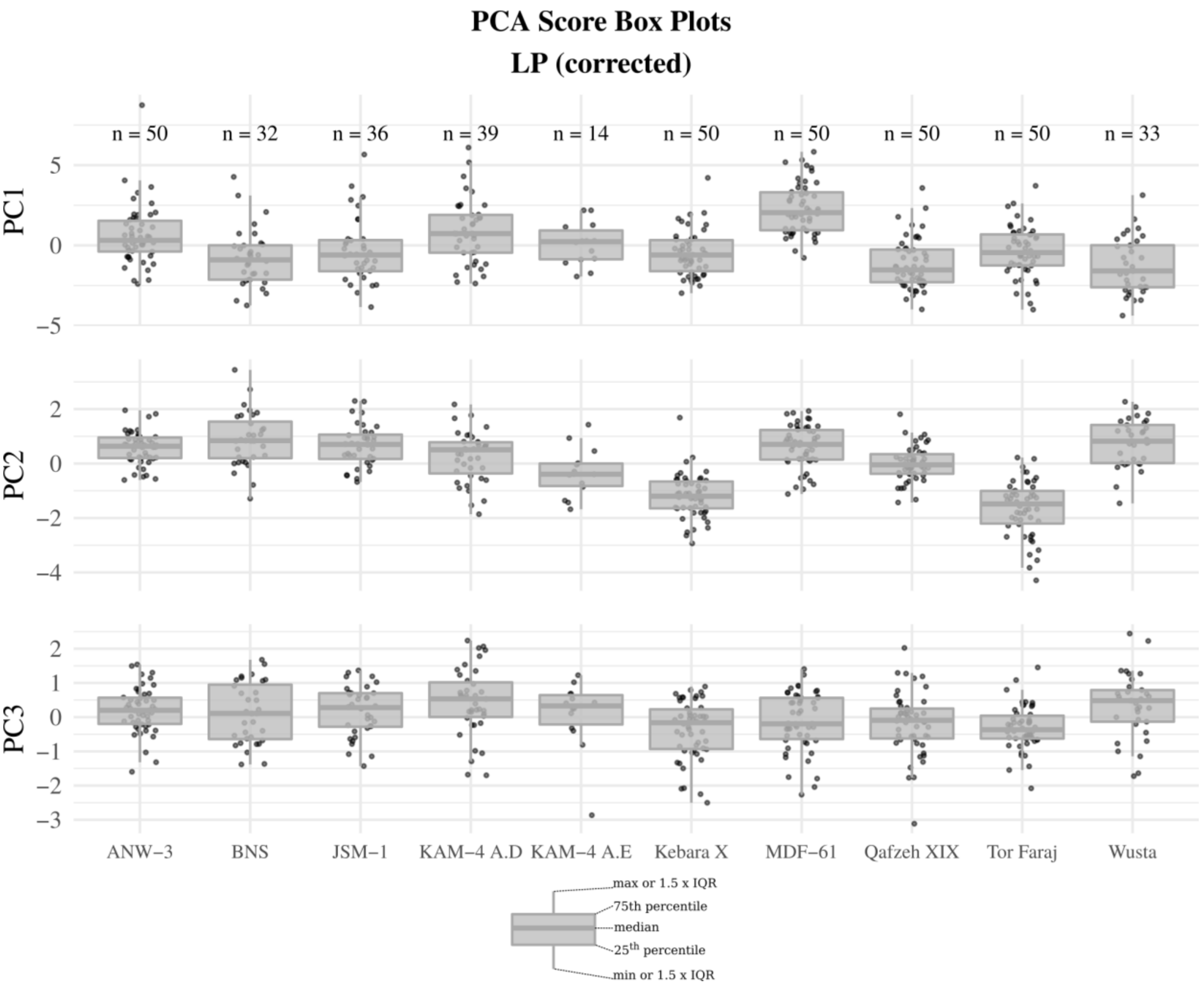

We would like to thank Kristjan Moore, a researcher at deCODE genetics (https://www.decode.com/), for bringing the duplication error to our attention.
